# Beverage consumption and energy intake among Canadians: analyses of 2004 and 2015 national dietary intake data

**DOI:** 10.1186/s12937-019-0488-5

**Published:** 2019-10-18

**Authors:** Amanda C. Jones, Sharon I. Kirkpatrick, David Hammond

**Affiliations:** 1 Department of Public Health, University of Otago Wellington, PO Box 7343, Wellington, Newtown 6242 New Zealand; 2 School of Public Health and Health Systems, University of Waterloo, 200 University Ave. W, Waterloo, ON N2L 3G1 Canada

**Keywords:** Beverage intake, Fluid intake, Nutrition surveillance, Sugar-sweetened beverages, Sugary drinks, Alcohol

## Abstract

**Background:**

Among adults and children consuming Western diets, beverages are significant sources of free sugars, saturated fats, excess calories, and alcohol, with relevance to chronic disease risk. The impact of recent healthy eating policies and beverage market evolutions on population-level consumption patterns in Canada is unknown. The current study examined trends in intake of a range of beverage types among a nationally-representative sample of Canadians, with stratification by socio-demographic characteristics.

**Methods:**

The 2004 (*n* = 34,775) and 2015 (*n* = 20,176) nutrition-focused cycles of the Canadian Community Health Surveys are cross-sectional surveys representative of the population of the 10 Canadian provinces. Based on a single multiple-pass 24-h dietary recall for each participant, fluids consumed as beverages were grouped into seven categories. Using linear regression, reported intake (volume, ml and energy, kcal) of each category was characterized over time and in relation to sex, age, ethnicity, income, body mass index (BMI), and province of residence.

**Results:**

In 2015, Canadians reported consuming an average of 1806 ml (275 kcal) fluids as beverages per day, including: plain water 867 ml (0 kcal); other unsweetened beverages, e.g. coffee, 364 ml (6 kcal); sugar-sweetened beverages (SSBs) 204 ml (99 kcal); plain milk 132 ml (64 kcal); alcoholic drinks 120 ml (71 kcal); 100% juice 74 ml (34 kcal); and diet or low calorie beverages 44 ml (2 kcal). Differential consumption was observed across socio-demographic groups, with high consumption of sugary drinks (i.e., SSBs and 100% juice) and alcohol across groups. From 2004 to 2015, the reported volumes of beverages consumed decreased by 10% (energy: − 24%). With adjustment for socio-demographic characteristics, there were significant changes (*p* < 0.001) over time in intake of: 100% juice − 40% (− 38%); plain milk − 37% (− 35%); SSBs − 26% (− 20%); diet or low calorie beverages (− 46%); and other unsweetened beverages − 11% (− 42%). The volume of plain water consumed increased by 10% (*p* < 0.0001). Intake of alcoholic (volume and energy) and diet or light beverages did not change significantly.

**Conclusions:**

Lower intake of beverages was reported by Canadians in 2015 versus 2004, with a shift towards plain water. Consumption of sugary drinks decreased, but these beverages continue to contribute substantially to Canadians' overall energy intake. The findings underscore the need for policies to further reduce the consumption of sugary and alcoholic beverages, as well as calories from beverages.

## Background

Diet-related diseases are a leading cause of death and disability worldwide [1]. Consistent with other countries, rates of diet-related diseases, including diabetes and obesity, have risen dramatically in Canada in recent years [2, 3]. Beverage intake, which is a component of diet, has an important influence on health and on risk for diet-related diseases. Although water is critical for life [4] and beverages are the primary source of water intake [5], beverages also contribute free sugars, saturated fats, excess calories, and alcohol [6–9], each of which has been linked to disease outcomes [10, 11]. In particular, consumption of sugary beverages has received substantial attention and been shown to be associated with increased risk of type 2 diabetes, obesity, obesity-related conditions including numerous cancers and cardiovascular disease, as well as dental caries [12–17]. Alcohol is similarly an important risk factor for chronic disease, as well as acute harms including injuries and interpersonal violence [18].

Evidence from high-income countries indicates recent decreases in consumption of traditional sugar-sweetened beverages, such as carbonated soft drinks and fruit drinks containing added sugar, alongside increased consumption of novel products, such as sweetened coffees, teas, energy drinks, and sports drinks [19–22]. Intake of 100% juice has also increased and consumers have switched from higher fat milks to reduced fat products [20, 22]. Among adults, alcohol remains a significant source of energy intake, though it is typically excluded from definitions of sugary drinks [22].

Despite the critical role of beverages in promoting or harming health, there is relatively little data on trends in beverage consumption for many countries, including Canada. As in most other countries, national nutrition surveys in Canada have been infrequent, with the two most recent surveys conducted in 2004 and 2015 [23]. Analyses of Canadian data from 2004 indicate that, among children and youth, beverages contributed 30% of daily energy; adults’ energy intake from beverages was lower and ranged from 11 to 20% depending on age and sex [24, 25]. Except among young children (1–8 years), water was the most consumed beverage by volume. Consumption was also characterized by high intake of sugary drinks, especially among children and youth, high alcohol intake among adults, and high milk intake among young children [24, 25].

Echoing global developments [26–28], since 2004, several policies have been implemented in Canada to promote healthy eating, including reduced sugar intake, such as provincial-level bans on the sale of sugar-sweetened beverages (SSBs) in schools [29]. The beverage industry has evolved over the same period to expand the diversity of beverages available, including products containing a wide range of sweeteners and beverages marketed for their ‘functional’ properties (e.g., caffeine, protein) [30]. The impact of evolutions in the market on population-level consumption patterns in Canada is unknown. The purpose of this study was thus to examine per capita daily beverage consumption among the Canadian population by 1) examining mean beverage intake (volume and energy) reported by Canadians in 2015; 2) identifying significant differences in reported beverage intake in 2015 according to sex, age, ethnicity, income, province, and body mass index (BMI) category; and 3) characterizing changes in reported beverage intake between 2004 and 2015, adjusted for socio-demographic variables.

## Methods

### Data sources

Beverage consumption was characterized using dietary intake data from the 2004 and 2015 Canadian Community Health Survey–Nutrition (CCHS-Nut) probability-based cross-sectional surveys, conducted by Statistics Canada and Health Canada [31–33]. Each CCHS-Nut consists of a General Health Survey and a standardized 24-h dietary recall (24HR), and provides nationally-representative estimates for Canadians residing in the 10 provinces (2004: ages ≥0 years, *N* = 35,107; 2015: ages ≥1 years, *N* = 20,487). The sampling frames cover 90–98% of the provincial populations. Excluded persons were those living on reserve and other Indigenous peoples’ settlements, full-time members of the Canadian Forces, and the institutionalized population. Respondents were limited to one person per household. Interviews were completed with parents or guardians for children under 6 years of age and proxy-assisted for those aged 6 to 11 years. The surveys’ methods are reported in detail elsewhere [31–33].

Using a computer-assisted interviewing tool, trained interviewers administered the General Health Survey and the multiple-pass 24HR to elicit details regarding foods and beverages consumed the previous day. A probabilistic sample of approximately 30% of respondents completed a second 24HR, 3 to 10 days later. Foods and beverages reported in the 24HR were then coded by Health Canada using a food composition database based on the Canadian Nutrient File. The current study drew upon data from the first dietary recall only and included all respondents with a valid recall as defined by Health Canada [31, 32].

To align the age ranges between the two survey cycles, infants (*n* = 355 respondents age < 1 year) were excluded from the CCHS-Nut 2004 dataset. The final samples (2004: *N* = 34,463; 2015: 20176) excluded respondents who exclusively consumed breastmilk, were pregnant, or were breastfeeding. The CCHS Master Files were accessed through the Statistics Canada South-Western Ontario Research Data Centre at the University of Waterloo. Ethics clearance from the University of Waterloo’s Office of Research Ethics was not required given the rigorous data protections in place within the Research Data Centre.

### Measures

#### Beverage intake

A range of beverage categories, distinguishing products based on the presence or absence of free sugars and including water and alcohol, were considered. The World Health Organization’s (WHO) definition of free sugars was adopted: monosaccharides and disaccharides added to foods and beverages, as well as honey, syrups, fruit juices, fruit concentrates, and other sugars that are naturally present in foods and beverages [34]. Given the health risks of free sugars, differentiating between beverages with or without free sugars is important [12, 13, 35, 36], though few studies make this distinction [21].

Based on food codes assigned by Health Canada, fluids consumed as beverages were first grouped into 37 mutually-exclusive sub-categories (Additional file 1: Appendix A) and then aggregated into seven mutually-exclusive categories (Table 1). Double-counting was eliminated by including only those beverages classified within the dataset as ‘basic’ or ‘recipe’ (i.e., an ‘as consumed’ format); beverages reported as ‘ingredients’ in recipes were excluded. For each beverage category, reported intake was analyzed by volume (ml) and energy (kcal). One gram of beverage was converted to 1 millilitre [25]. Non-consumers were assigned zero values for volume and energy variables, permitting the calculation of per capita estimates.

**Table 1:** Beverage category definitions

Beverage category	Beverages included
Plain water	Plain bottled, tap, or well water
Other unsweetened beverages	Club soda; unsweetened: coffee, tea, flavoured milk
Sugar-sweetened beverages (SSBs)	Regular, sweetened: carbonated soft drinks, fruit drinks, sports drinks, energy drinks, flavoured water, coffee, tea, hot chocolate, flavoured milk or substitutes, meal replacement beverages, protein drinks, smoothies, drinkable yogurt
Plain milk	Unsweetened, unflavoured: milk or substitutes
Alcoholic beverages	Beer, wine, spirits, liqueur, cocktails, coolers
100% juice	100% juice, including ‘baby juices’
Diet or light beverages	Diet or light: carbonated soft drinks, fruit drinks, sports drinks, energy drinks, flavoured water, coffee, tea, hot chocolate, flavoured milk or substitutes, meal replacement beverages, protein drinks

#### Socio-demographic measures

Previous research examining the CCHS-Nut 2004 dataset reported significant differences in reported beverage consumption by socio-demographic characteristics [24, 25, 37–40]. Socio-demographic variables for inclusion in this analysis were identified based on these known associations and their role as potential confounders in temporal trends. Variables of interest available for both survey cycles included sex (male, female), age (continuous), ethnicity (13 binary variables for Aboriginal, white, Chinese, South Asian, Black, Filipino, Latin American, Southeast Asian, Arab, West Asian, Japanese, Korean, Other), total household income (continuous), province, and BMI category (adults ≥17 years: underweight, normal weight, overweight, obese class I, obese class II, obese class III; school-aged children 5–17 years: thin, normal, overweight, obese; preschool-aged children < 5 years: thin, normal, at risk of overweight, overweight, obese) [41–43].

Based on observed frequencies, age was recoded into five categories (1–8, 9–18, 19–30, 31–50, 51+). Ethnicity was recoded into six categories (white only, Chinese only, South Asian only, Black only, Indigenous inclusive, mixed/other/not stated/missing). To calculate per capita income, each respondent’s total household income was divided by the square root of the respondent’s household size. Using the square root of household size as an equivalence scale accounts for economies of scale in consumption [44, 45]. With survey weights applied, per capita income was separated into quartiles ranging from 1 (low income) to 4 (high income); non-respondents were coded into a fifth ‘not reported’ category. BMI was recoded into four categories [underweight/normal weight (includes at risk of overweight), affected by overweight, affected by obesity, and don’t know/refusal/not stated].

### Analysis

Beverage intake in 2015 is reported using mean [95% confidence intervals, 95% CI)] volume (ml) and energy (kcal) for total beverage intake and each beverage category. To examine associations between intake and sociodemographic covariates, linear models using generalized least squares regression were constructed using each beverage category’s volume and energy as dependent variables (no model was constructed for energy intake contributed by plain water). All models included sex, age, ethnicity, income, BMI category, and province as covariates. Pairwise t-test comparisons tested for differences among categories for each variable (Additional file 1: Appendix B). For comparison purposes, beverage intake in 2004 by socio-demographic characteristics is reported in the Additional file 1: Appendix C. Also reported are intakes of beverage sub-categories for all respondents and by age-sex group (males 1–18, females 1–18, males 19+, females 19+; Additional file 1: Appendix D and Appendix E).

To examine differences in intake between 2004 and 2015, the relative changes (% difference) in volume and energy for total beverages and each beverage category were calculated. Generalized least squares regression was applied with volume and energy for each beverage category as dependent variables. An indicator variable for survey year was included in the model, along with the socio-demographic covariates.

A bootstrap resampling method was applied to account for variance resulting from the surveys’ stratified multi-cluster designs [31, 32]. The bootstrapped weights prepared by Statistics Canada and Health Canada were applied in the statistical software SAS (version 9.4; SAS Institute Inc., Cary, North Carolina, USA; 2016) using PROC SURVEYMEANS (means and 95% CI) or PROC SURVEYREG (linear regression) with the BRR option. Statistical significance was set at *p* < 0.05. The Benjamini-Hochberg procedure was applied with a false discovery rate of 0.05 as a post hoc adjustment to variables with multiple comparisons [46]. All reported sample sizes are weighted.

## Results

### Sample characteristics

Table 2 outlines the socio-demographic characteristics of the weighted samples for the 2004 and 2005 CCHS cycles.

**Table 2:** Sample socio-demographic characteristics, weighted

	2004 *N* = 34,463		2015 *N* = 20,176	
	%	n	%	n
Sex				
Male	50.3	17,330	50.0	10,096
Female	49.7	17,133	50.0	10,080
Age (years)				
1–8	9.2	3171	8.9	1800
9–18	13.6	4668	11.1	2230
19–30	16.1	5538	13.0	2622
31–50	31.6	10,882	30.5	6150
51+	29.5	10,145	36.5	7374
Ethnicity				
White only	82.5	28,383	71.6	14,452
Chinese only	3.1	1085	4.5	915
South Asian only	3.7	1263	4.9	994
Black only	2.1	710	3.5	706
Indigenous inclusive	1.8	628	3.0	606
Mixed/other/not stated/missing	6.8	2335	12.4	2504
Income				
1 (lowest income)	18.6	6427	18.7	3758
2	19.6	6750	19.1	3854
3	19.0	6541	18.0	3634
4 (highest income)	19.0	6550	18.8	3796
Not reported	23.8	8195	25.4	5135
Province				
Newfoundland and Labrador	1.7	571	1.5	300
Prince Edward Island	0.4	152	0.4	83
Nova Scotia	3.0	1021	2.6	531
New Brunswick	2.4	813	2.1	421
Quebec	23.8	8192	23.3	4704
Ontario	39.3	13,531	38.8	7834
Manitoba	3.5	1208	3.4	694
Saskatchewan	2.9	1020	3.1	613
Alberta	9.9	3400	11.7	2352
British Columbia	13.1	4497	13.1	2644
Body Mass Index category				
Underweight/normal	27.1	9319	29.7	6002
Overweight	19.1	6571	21.4	4308
Obese	12.2	4204	15.4	3115
Don’t know/refusal/not stated	41.6	14,309	33.5	6752

### 2015 Beverage consumption

**Fig. 1:**
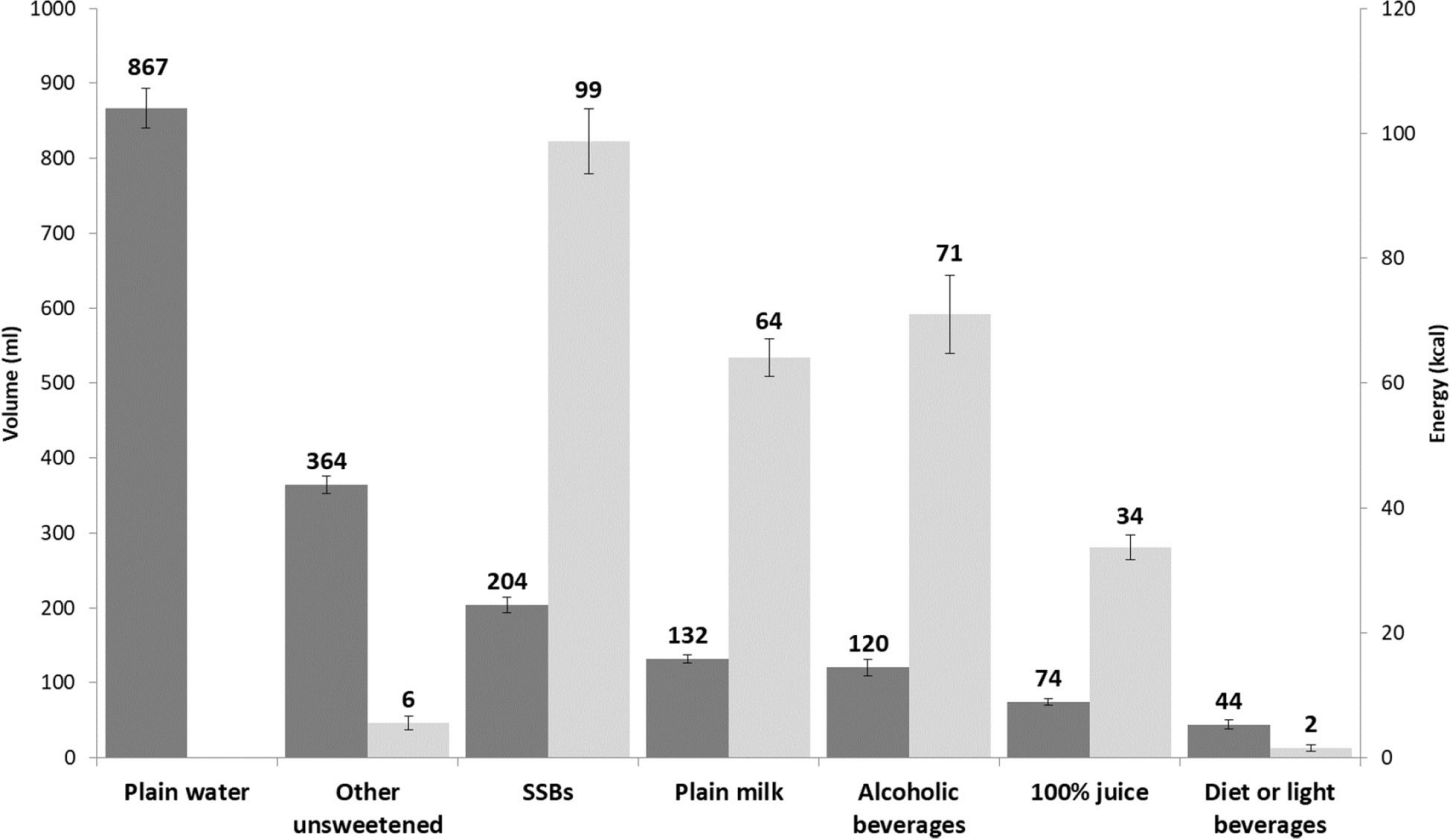
Daily per capita beverage volume and energy intakes in 2015 (*N* = 20,176). Dark shading indicates volume (ml), light shading indicates energy (kcal). Data source: 2015 Canadian Community Health Survey–Nutrition. Error bars indicate 95% confidence interval. Plain water contained no energy. Abbreviations: kcal, kilocalorie; ml, millilitre; SSBs, sugar-sweetened beverages

In 2015, the mean reported consumption of all beverages was 1806 ml (275 kcal) per capita, per day. As Fig. 1 indicates, plain water was consumed in the highest volume, whereas SSBs contributed the most to energy intake, followed by alcohol.

Tables 3 and 4 show beverage intake by socio-demographic group. The pairwise comparisons are reported in the Additional file 1: Appendix B.

**Table 3:** Daily per capita beverage volume intake (ml) from beverage categories in 2015, by socio-demographic characteristic (*N* = 20,176)

		Plain water		Other unsweetened		SSBs		Plain milk		Alcoholic beverages		100% juice		Diet or light beverages	
		Mean volume in ml (95% CI) Adjusted p-value		Mean volume in ml (95% CI) Adjusted *p*-value		Mean volume in ml (95% CI) Adjusted p-value		Mean volume in ml (95% CI) Adjusted p-value		Mean volume in ml (95% CI) Adjusted p-value		Mean volume in ml (95% CI) Adjusted p-value		Mean volume in ml (95% CI) Adjusted p-value	
Model statistics	n	F_(26,20,176)_ = 57.08		F_(26,20,176)_ = 170.71		F_(26,20,176)_ = 57.06		F_(26,20,176)_ = 73.54		F_(26,20,176)_ = 62.69		F_(26,20,176)_ = 43.04		F_(1,20,176)_ = 27.22	
Sex		*p* = 0.6492		*p* = 0.0052		*p* < .0001		*p* < .0001		*p* < .0001		*p* < .0001		*p* = 0.5069	
Male	10,096	879.0	(836.4, 921.6)	379.5	(360.9, 398.0)	246.8	(229.6, 264.1)	143.6	(134.9, 152.3)	177.8	(157.1, 198.4)	85.9	(78.6, 93.1)	47.7	(38.9, 56.6)
Female	10,080	855.5	(825.8, 885.1)	349.3	(334.6, 364.1)	160.2	(149.6, 170.9)	119.9	(112.8, 127.1)	62.7	(55.4, 69.9)	62.7	(58.1, 67.4)	40.6	(33.5, 47.7)
Age (years)		*p* < .0001		*p* < .0001		*p* < .0001		*p* < .0001		*p* < .0001		*p* < .0001		*p* < .0001	
1–8	1800	444.9	(418.8, 471.1)	4.9	(3.0, 6.7)	129.7	(116.8, 142.5)	285.7	(268.0, 303.4)	0.8	(0.0, 1.9)*	119.7	(109.4, 130.1)	5.6	(2.7, 8.6)
9–18	2230	806.9	(771.3, 842.6)	47.3	(40.2, 54.4)	302.7	(281.5, 323.8)	206.4	(194.1, 218.7)	7.9	(4.4, 11.4)	116.9	(107.1, 126.6)	17.3	(13.3, 21.3)
19–30	2622	1092.0	(980.0, 1204.1)	287.9	(252.0, 323.8)	327.1	(277.2, 377.0)	119.0	(91.6, 146.4)	170.4	(120.3, 220.5)	91.3	(70.9, 111.7)	23.4	(16.3, 30.6)
31–50	6150	1006.1	(950.9, 1061.3)	452.7	(427.8, 477.7)	214.4	(195.6, 233.2)	95.1	(85.8, 104.4)	154.7	(131.6, 177.8)	60.5	(52.0, 69.0)	58.3	(44.8, 71.9)
51+	7374	792.9	(760.0, 825.7)	501.7	(485.0, 518.3)	138.7	(127.8, 149.7)	106.8	(100.0, 113.5)	136.9	(121.8, 151.9)	55.8	(50.8, 60.8)	57.2	(47.6, 66.8)
Ethnicity		*p* = 0.0100		*p* < .0001		*p* < .0001		*p* < .0001		*p* < .0001		*p* = 0.1130		*p* < .0001	
White only	14,452	876.3	(848.2, 904.5)	398.8	(384.6, 413.0)	203.9	(190.9, 216.9)	136.9	(130.4, 143.4)	149.0	(135.1, 162.9)	74.2	(69.0, 79.4)	52.8	(45.1, 60.4)
Chinese only	915	713.3	(636.9, 789.7)	340.3	(274.1, 406.4)	116.4	(88.5, 144.3)	97.8	(80.7, 114.9)	13.9	(7.1, 20.6)	51.3	(36.8, 65.7)	4.7	(1.3, 8.1)
South Asian only	994	849.4	(767.6, 931.2)	214.3	(180.0, 248.5)	197.4	(158.1, 236.7)	188.2	(149.1, 227.3)	19.8	(10.0, 29.7)	64.0	(49.5, 78.6)	21.2	(9.2, 33.2)
Black only	706	777.3	(640.8, 913.8)	158.9	(122.8, 194.9)	221.7	(182.7, 260.7)	92.5	(61.3, 123.8)	30.3	(15.2, 45.4)	97.5	(72.1, 123.0)	12.8	(0.0, 29.7)*
Indigenous inclusive	606	896.4	(798.4, 994.4)	337.6	(282.5, 392.7)	303.4	(241.0, 365.9)	134.1	(107.4, 160.8)	72.9	(47.5, 98.3)	79.3	(52.4, 106.2)	61.1	(34.3, 87.9)
Mixed/other/not stated/ missing	2504	896.4	(807.6, 985.2)	299.0	(257.2, 340.9)	206.8	(178.8, 234.9)	102.8	(90.5, 115.0)	70.1	(34.6, 105.6)	79.5	(67.0, 92.1)	22.7	(13.5, 31.9)
Income quartile		*p* < .0001		*p* = 0.0213		*p* = 0.2543		*p* = 0.5967		*p* = 0.0003		*p* = 0.8946		*p* = 0.0288	
1 (lowest income)	3758	741.1	(684.4, 797.9)	363.1	(335.4, 390.8)	208.3	(185.5, 231.0)	141.0	(123.4, 158.7)	69.5	(54.5, 84.5)	74.4	(65.1, 83.6)	36.3	(27.0, 45.5)
2	3854	815.8	(758.8, 872.8)	323.0	(301.9, 344.1)	199.3	(178.0, 220.7)	132.1	(120.3, 143.9)	108.3	(89.6, 126.9)	79.2	(68.9, 89.4)	33.3	(26.5, 40.0)
3	3634	914.3	(852.9, 975.8)	379.0	(352.6, 405.4)	209.8	(189.8, 229.9)	135.5	(121.8, 149.2)	121.2	(99.9, 142.5)	73.2	(64.4, 82.0)	50.7	(37.5, 63.8)
4 (highest income)	3796	1048.2	(986.2, 1110.2)	424.0	(393.1, 455.0)	191.8	(167.4, 216.3)	124.6	(113.6, 135.5)	173.4	(142.6, 204.1)	65.4	(55.2, 75.6)	71.6	(52.6, 90.6)
Not reported	5135	831.1	(781.1, 881.0)	342.1	(316.6, 367.6)	207.6	(185.4, 229.8)	127.4	(117.8, 137.0)	126.5	(98.2, 154.9)	77.9	(67.8, 88.1)	33.2	(21.6, 44.7)
Province		*p* < .0001		*p* = 0.0054		*p* < .0001		*p* = 0.0002		*p* = 0.0135		*p* < .0001		*p* < .0001	
NL	300	651.4	(581.6, 721.1)	384.6	(337.9, 431.4)	233.5	(192.7, 274.3)	110.5	(96.5, 124.5)	104.5	(65.9, 143.1)	68.5	(55.4, 81.6)	83.5	(58.7, 108.2)
PEI	83	680.8	(613.8, 747.7)	387.1	(352.9, 421.4)	207.7	(181.6, 233.8)	153.9	(136.8, 171.0)	105.0	(67.8, 142.2)	75.7	(56.2, 95.2)	54.2	(34.1, 74.2)
Nova Scotia	531	839.8	(745.6, 933.9)	386.3	(353.5, 419.1)	208.2	(178.2, 238.2)	137.2	(121.2, 153.2)	134.8	(86.6, 182.9)	63.3	(52.1, 74.4)	52.0	(38.7, 65.3)
New Brunswick	421	826.1	(740.7, 911.5)	389.3	(348.2, 430.5)	234.3	(201.3, 267.2)	144.0	(124.8, 163.1)	124.7	(88.8, 160.6)	66.9	(54.8, 78.9)	55.3	(38.2, 72.4)
Quebec	4704	809.9	(756.6, 863.2)	336.8	(310.1, 363.6)	192.9	(171.0, 214.7)	146.1	(130.6, 161.7)	146.9	(119.8, 174.1)	116.9	(103.5, 130.2)	50.6	(35.6, 65.7)
Ontario	7834	827.4	(779.2, 875.6)	360.1	(340.0, 380.2)	208.5	(189.3, 227.8)	124.8	(114.4, 135.1)	101.0	(84.8, 117.2)	64.1	(57.7, 70.5)	38.7	(29.4, 48.0)
Manitoba	694	905.5	(816.1, 995.0)	371.8	(336.0, 407.5)	249.5	(217.5, 281.5)	147.4	(131.3, 163.6)	79.0	(58.0, 100.1)	61.5	(49.8, 73.3)	68.9	(40.0, 97.8)
Saskatchewan	613	882.3	(799.1, 965.6)	440.1	(391.7, 488.4)	204.7	(172.3, 237.1)	160.7	(139.7, 181.8)	103.0	(55.4, 150.7)	61.5	(49.1, 73.9)	45.9	(29.1, 62.6)
Alberta	2352	1028.4	(946.4, 1110.5)	358.6	(322.4, 394.7)	246.6	(219.6, 273.6)	133.5	(121.0, 146.1)	137.5	(98.8, 176.1)	54.9	(45.4, 64.5)	58.3	(41.2, 75.4)
British Columbia	2644	972.8	(891.6, 1053.9)	400.7	(371.4, 430.1)	148.1	(131.5, 164.6)	113.3	(102.1, 124.5)	128.1	(105.3, 150.9)	56.3	(47.0, 65.6)	21.0	(14.4, 27.6)
BMI category		*p* < .0001		*p* = 0.7782		*p* = 0.6788		*p* = 0.4117		*p* = 0.2204		*p* = 0.0142		*p* < .0001	
Underweight/normal	6002	774.2	(738.0, 810.3)	288.2	(268.4, 308.0)	213.5	(196.3, 230.8)	143.0	(132.4, 153.6)	102.5	(86.6, 118.3)	92.6	(84.4, 100.9)	17.9	(13.5, 22.4)
Overweight	4308	915.2	(858.1, 972.2)	387.7	(362.2, 413.2)	194.2	(178.0, 210.3)	126.9	(114.4, 139.3)	120.1	(98.0, 142.3)	67.3	(59.1, 75.6)	52.2	(36.8, 67.5)
Obese	3115	1025.6	(951.9, 1099.3)	419.8	(389.6, 450.0)	217.9	(187.5, 248.3)	127.3	(114.2, 140.3)	144.8	(115.7, 173.9)	61.3	(52.0, 70.6)	89.0	(67.9, 110.0)
Don’t know/refusal/not stated	6752	846.3	(796.6, 896.1)	391.8	(368.9, 414.8)	194.2	(175.3, 213.0)	127.0	(117.3, 136.7)	124.8	(103.8, 145.8)	68.4	(60.7, 76.2)	41.6	(33.6, 49.7)

All means are weighted ‘arithmetic means’ (also referred to as ‘observed means’), and not linear regression adjusted least squares means. Models statistics are for separate linear models using generalized least squares regression for each beverage category, with covariates sex, age, ethnicity, income, province, and BMI category; α = 0.05

*Abbreviations*: *95% CI* 95% confidence interval, *BMI* Body-mass index, *ml* millilitre, *NF* Newfoundland and Labrador, *PEI* Prince Edward Island, *SSBs* Sugar-sweetened beverages

*Estimates for these 95% confidence intervals contained values less than zero. For reporting, these values were replaced with ‘0’ as they were a result of the bootstrap resampling method and not an indication of negative consumption

**Table 4:** Daily per capita energy intake (kcal) from beverage categories in 2015, by socio-demographic variable (*N* = 20,176)

		Other unsweetened		SSBs		Plain milk		Alcoholic beverages		100% juice		Diet or light beverages	
		Mean energy in kcal (95% CI) Adjusted p-value		Mean energy in kcal (95% CI) Adjusted p-value		Mean energy in kcal (95% CI) Adjusted p-value		Mean energy in kcal (95% CI) Adjusted p-value		Mean energy in kcal (95% CI) Adjusted p-value		Mean energy in kcal (95% CI) Adjusted p-value	
Model statistics	n	F_(26,20,176)_ = 15.46		F_(26,20,176)_ = 55.35		F_(26,20,176)_ = 85.49		F_(26,20,176)_ = 51.42		F_(26,20,176)_ = 43.51		F_(1,20,176)_ = 16.30	
Sex		*p* = 0.4423		*p* < .0001		*p* < .0001		*p* < .0001		*p* < .0001		*p* = 0.3181	
Male	10,096	5.1	(3.2, 7.0)	119.9	(111.1, 128.7)	70.3	(65.7, 74.9)	96.5	(85.5, 107.4)	38.7	(35.3, 42.0)	1.4	(1.1, 1.7)
Female	10,080	6.0	(5.0, 6.9)	77.5	(72.1, 82.9)	57.9	(54.2, 61.5)	45.4	(39.8, 51.1)	28.7	(26.6, 30.9)	1.7	(0.8, 2.6)
Age (years)		*p* < .0001		*p* < .0001		*p* < .0001		*p* < .0001		*p* < .0001		*p* < .0001	
1–8	1800	0.2	(0.0, 0.4)	74.0	(66.4, 81.6)	149.8	(140.5, 159.1)	0.6	(0.0, 1.6)*	56.3	(51.4, 61.3)	0.3	(0.1, 0.5)
9–18	2230	1.1	(0.3, 1.8)	152.0	(141.0, 163.0)	98.9	(93.0, 104.7)	5.0	(2.5, 7.4)	54.5	(49.9, 59.1)	0.8	(0.5, 1.2)
19–30	2622	5.1	(3.0, 7.2)	165.6	(139.0, 192.2)	61.0	(45.3, 76.6)	91.0	(66.1, 115.9)	41.7	(32.1, 51.2)	0.8	(0.5, 1.2)
31–50	6150	8.8	(5.7, 12)	101.1	(90.3, 111.8)	45.1	(40.7, 49.6)	86.2	(73.1, 99.3)	27.1	(23.2, 31.0)	1.5	(1.1, 1.9)
51+	7374	5.6	(4.9, 6.3)	62.9	(57.4, 68.3)	49.6	(46.4, 52.8)	88.3	(79.1, 97.4)	24.6	(22.3, 26.8)	2.4	(1.1, 3.7)
Ethnicity		*p* = 0.0975		*p* < .0001		*p* < .0001		*p* < .0001		*p* = 0.1870		*p* = 0.0015	
White only	14,452	5.5	(4.6, 6.3)	97.7	(91.4, 104.0)	65.3	(62.0, 68.6)	86.5	(78.9, 94.1)	33.3	(30.9, 35.7)	1.9	(1.2, 2.6)
Chinese only	915	5.4	(0.4, 10.3)	53.2	(40.8, 65.6)	48.8	(40.1, 57.5)	8.8	(4.8, 12.8)	23.9	(17.2, 30.7)	0.2	(0.0, 0.5)*
South Asian only	994	3.2	(2.0, 4.4)	103.5	(78.4, 128.7)	98.4	(76.1, 120.6)	17.5	(3.9, 31.1)	30.5	(23.4, 37.5)	0.7	(0.2, 1.2)
Black only	706	2.7	(0.6, 4.7)	107.0	(85.4, 128.6)	48.4	(31.7, 65.0)	23.2	(10.8, 35.6)	46.0	(33.6, 58.4)	0.7	(0.0, 1.6)*
Indigenous inclusive	606	2.0	(0.8, 3.3)	141.6	(112.6, 170.6)	67.3	(53.5, 81.1)	45.5	(28.0, 63.1)	36.9	(23.9, 49.9)	2.5	(0.8, 4.2)
Mixed/other/not stated/missing	2504	8.7	(2.6, 14.9)	106.5	(82.2, 130.8)	52.9	(46.4, 59.4)	44.8	(24.0, 65.6)	36.6	(30.9, 42.4)	0.6	(0.3, 0.8)
Income quartile		*p* = 0.3040		*p* = 0.4077		*p* = 0.3396		*p* < .0001		*p* = 0.8863		*p* = 0.2099	
1 (lowest income)	3758	7.1	(2.9, 11.3)	97.0	(85.7, 108.3)	71.7	(61.5, 82.0)	39.5	(31.5, 47.5)	34.6	(29.9, 39.3)	0.9	(0.7, 1.2)
2	3854	4.1	(3.2, 5.0)	92.4	(83.6, 101.1)	64.5	(58.6, 70.4)	68.0	(55.8, 80.2)	35.9	(31.2, 40.7)	1.0	(0.7, 1.2)
3	3634	5.3	(3.3, 7.3)	97.3	(87.1, 107.6)	65.2	(58.3, 72.0)	73.8	(61.6, 85.9)	33.1	(29.1, 37.1)	1.2	(0.9, 1.5)
4 (highest income)	3796	6.2	(4.6, 7.8)	98.8	(84.0, 113.6)	57.5	(52.5, 62.6)	99.0	(83.5, 114.4)	29.0	(24.3, 33.6)	3.8	(1.2, 6.3)
Not reported	5135	5.2	(4.1, 6.4)	105.6	(92.2, 119.1)	62.3	(57.5, 67.1)	73.5	(57.3, 89.7)	35.3	(30.7, 39.8)	1.1	(0.8, 1.5)
Province		*p* < .0001		*p* < .0001		*p* = 0.0001		*p* = 0.0029		*p* < .0001		*p* < .0001	
NL	300	3.4	(2.5, 4.3)	110.2	(90.6, 129.8)	52.4	(45.5, 59.3)	60.2	(41.4, 78.9)	32.2	(25.9, 38.4)	2.3	(1.5, 3.0)
PEI	83	3.1	(1.6, 4.6)	104.1	(90.9, 117.3)	72.3	(64.0, 80.6)	55.7	(38.9, 72.4)	35.1	(26.0, 44.2)	1.4	(0.9, 1.9)
Nova Scotia	531	3.1	(2.3, 3.9)	99.4	(85.4, 113.4)	63.8	(56.7, 70.9)	71.9	(51.9, 91.9)	28.9	(23.8, 34.1)	1.8	(1.0, 2.6)
New Brunswick	421	3.2	(1.7, 4.8)	114.9	(97.7, 132.2)	67.5	(58.7, 76.3)	73.2	(52.6, 93.8)	30.6	(25.1, 36.0)	2.4	(1.3, 3.5)
Quebec	4704	9.6	(5.6, 13.6)	90.5	(80.0, 101.0)	74.5	(65.8, 83.2)	89.7	(72.7, 106.6)	52.5	(46.2, 58.8)	1.2	(0.9, 1.6)
Ontario	7834	4.0	(2.9, 5.0)	102.8	(92.2, 113.5)	59.8	(54.8, 64.8)	56.0	(47.8, 64.2)	29.4	(26.4, 32.4)	1.8	(0.7, 3.0)
Manitoba	694	3.4	(1.9, 4.9)	112.9	(97.6, 128.2)	70.5	(62.9, 78.1)	55.5	(40.1, 71.0)	28.3	(23.1, 33.4)	2.8	(1.2, 4.3)
Saskatchewan	613	2.0	(1.2, 2.7)	96.0	(81.5, 110.6)	76.7	(66.6, 86.8)	59.7	(36.4, 83.0)	28.7	(22.8, 34.6)	1.4	(0.9, 1.9)
Alberta	2352	4.7	(2.5, 6.9)	123.0	(106.1, 139.9)	64.2	(58.1, 70.3)	83.1	(61.1, 105.1)	24.7	(20.4, 28.9)	1.9	(1.0, 2.8)
British Columbia	2644	6.4	(4.1, 8.6)	72.2	(63.5, 80.9)	54.4	(49.0, 59.8)	79.0	(66.1, 91.9)	25.2	(20.8, 29.5)	0.6	(0.3, 0.8)
BMI category		*p* = 0.2642		*p* = 0.2394		*p* = 0.5178		*p* = 0.1439		*p* = 0.0268		*p* = 0.0009	
Underweight/normal	6002	5.6	(4.2, 7.0)	109.0	(99.2, 118.8)	70.3	(64.7, 75.8)	63.3	(53.8, 72.8)	41.9	(38.1, 45.7)	0.6	(0.4, 0.8)
Overweight	4308	5.7	(4.2, 7.2)	89.7	(82.2, 97.2)	61.3	(54.3, 68.3)	73.0	(61.1, 84.9)	30.9	(26.8, 35.0)	1.5	(1.1, 1.9)
Obese	3115	4.8	(3.5, 6.1)	97.9	(84.2, 111.7)	59.3	(53.2, 65.4)	84.3	(69.1, 99.5)	27.1	(23.0, 31.2)	4.0	(1.1, 6.9)
Don’t know/refusal/not stated	6752	5.7	(2.9, 8.5)	95.6	(84.3, 106.9)	62.6	(57.8, 67.4)	70.4	(58.1, 82.7)	31.3	(27.7, 34.8)	1.3	(0.9, 1.7)

No model was constructed for plain water as this beverage category contained no energy. All means are weighted ‘arithmetic means’ (also referred to as ‘observed means’), and not linear regression adjusted least squares means. Models statistics are for separate linear models using generalized least squares regression for each beverage category, with covariates sex, age, ethnicity, income, province, and BMI category; α = 0.05

*Abbreviations*: *95% CI* 95% confidence interval, *BMI* Body mass index, *kcal* kilocalorie, *NF* Newfoundland and Labrador, *PEI* Prince Edward Island, *SSBs* Sugar-sweetened beverages

*Estimates for these 95% confidence intervals contained values less than zero. For reporting, these values were replaced with ‘0’ as they were a result of the bootstrap resampling method and not an indication of negative consumption

#### Sex

For SSBs, alcohol, 100% juice, and milk, males consumed significantly more, both in terms of volume and calories, compared with females. Males also consumed significantly more volume of other unsweetened beverages. For SSBs, males reported consuming more than double the volume compared with females, with the contribution to energy from this category also double compared with females. For alcohol, males consumed almost triple the volume and double the calories consumed by females. For water and diet beverages, there were no significant differences between males’ and females’ reported intakes by volume or energy contribution.

#### Age

Age was associated with intake of all beverage categories, and numerous pairwise comparisons were significant. For 100% juice and milk, reported mean intake was highest among young children (1–8 years) and was lower among older age groups. Adults aged 19–30 years reported higher SSB consumption than all other age groups except 9–18 years. Compared with children, adults’ alcohol consumption was significantly higher. Water consumption was significantly different among almost all age groups; the highest reported intake was among adults aged 19–30 years. Intake of other unsweetened beverages was significantly higher with increasing age. Intake of diet beverages was also significantly higher with increasing age, then held constant from 31 to 50 years.

#### Ethnicity

Ethnicity was significantly associated with intake of all beverage categories, except 100% juice and energy intake of ‘other unsweetened beverages’. Respondents indicating Indigenous ethnicity consumed the highest reported volume of SSBs compared to all other ethnicities. Those of Indigenous ethnicity had the highest intakes of energy from SSB, diet beverage energy and volume, and water volume; the differences were significant compared to only some ethnicity groups.

Among those of white ethnicity, intake of alcohol was higher compared to all other ethnicity groups. Those of white ethnicity reported the highest volume of ‘other unsweetened beverages’, with some significant differences compared with other ethnicities. Milk intake was highest among those of South Asian ethnicity compared to all groups except white. Compared with all other groups, respondents of Chinese ethnicity reported the lowest intake of SSBs. Compared to most other groups, those of Chinese ethnicity also reported the lowest consumption of diet beverages, water, and alcohol. Those of Black ethnicity reported the lowest intake of milk, which was significantly lower compared with that reported among those of South Asian or white ethnicity, and lower intake of other unsweetened beverages, which was significantly lower compared with all other ethnicities.

#### Income

Compared with other income groups, respondents in Quartile 4 (highest income) reported significantly higher consumption of water whereas those in Quartile 1 (lowest income) reported significantly lower consumption of water (Table 3). For Quartile 1, alcohol intake was lowest but the difference was significant compared only with Quartile 4 and ‘income not reported’. Alcohol intake (volume and energy) was highest among those in Quartile 4, but was not significantly different compared with other groups. Other unsweetened beverage volume was lowest among those in Quartile 2 compared with those in Quartile 3 and Quartile 4. Respondents in Quartile 4 consumed significantly more diet beverages volume than those in Quartile 2.

#### BMI category

BMI category was significantly associated with intake of 100% juice, diet beverages, and water. Intake of 100% juice was highest and intake of diet beverages was lowest among those characterized as underweight/normal compared to those affected by overweight and by obesity. Intake of 100% juice intake was lowest and diet beverage intake was highest among those affected by obesity, with some significant differences compared to other body weight groups. For water, there appeared to be a gradient whereby intake was significantly higher with increasing BMI category.

#### Province

Province was significantly associated with intake of all beverage categories, and some pairwise comparisons were significant. Table 5 and Additional file 1: Appendix B report details of all provincial-level comparisons. In brief, respondents from British Columbia had the lowest mean reported intake of SSBs and diet beverages. Respondents from Alberta reported the highest intakes of SSBs energy and water, and the lowest intake of 100% juice. Those from Manitoba had the highest intake of SSBs volume and lowest intake of alcohol. Respondents from Saskatchewan reported consuming the most milk and volume of unsweetened beverages. Respondents from Quebec had the highest 100% juice and alcohol intakes, and the lowest unsweetened beverage intake. Among respondents from NL, the reported intake of diet beverage volume was highest; milk and water intakes were the lowest.

**Table 5:** Change between 2004 (*N* = 34,463) and 2015 (*N* = 20,176) average per capita daily beverage consumption (*N* = 54,579)

	Change between 2004 and 2015		Adjusted *p*-value
	Relative change (% difference)	Absolute change ml kcal	
Plain water			
Volume	+ 10%	77.5	*p* < 0.0001
Other unsweetened beverages			
Volume	−11%	−45.6	*p* < 0.0001
Energy	−42%	−4.1	*p* < 0.0001
SSBs			
Volume	−26%	−70.2	*p* < 0.0001
Energy	−20%	−24.7	*p* < 0.0001
Plain milk			
Volume	−37%	−76.0	*p* < 0.0001
Energy	−35%	− 35.3	*p* < 0.0001
Alcoholic beverages			
Volume	−13%	−18.1	*p* = 0.1928
Energy	−3%	−1.8	*p* = 0.7314
100% juice			
Volume	−40%	−50.6	*p* < 0.0001
Energy	−38%	−20.5	*p* < 0.0001
Diet or light beverages			
Volume	−15%	−8.0	*p* = 0.0557
Energy	−46%	−1.3	*p* < 0.001

Model statistics are for separate linear models using generalized least squares regression for each beverage category, with survey year as the independent variable and the covariates sex, age, ethnicity, income, province, and BMI category; α = 0.05. No model was constructed for plain water as this beverage category contained no energy. *Abbreviations*: *kcal* kilocalorie, *ml* millilitre, *Non. sig.* statistically non-significant, *SSBs* Sugar-sweetened beverages

### Changes in consumption between 2004 and 2015

Compared with 2004, reported per capita daily consumption of all beverages decreased by 10% for volume and 24% for energy in 2015. After adjustment for covariates (Table 5), water intake significantly increased from 2004 to 2015, while intake of other beverages (except alcohol and volume of diet beverages) significantly decreased.

The Additional file contains additional results reporting 2004 intake by socio-demographic variable (Additional file 1: Appendix C), as well as 2004 and 2015 intakes of all beverage categories and sub-categories for all respondents and by age-sex group (Additional file 1: Appendix D, Appendix E).

## Discussion

In 2015, Canadians’ reported beverage consumption averaged 1806 ml (275 kcal) per person per day, with significant variations by socio-demographic characteristics. For all beverages combined, significant declines of − 10% by volume and − 24% by energy were observed between 2004 and 2015, equating with meaningful reductions in the volume and energy consumed from this source. Reductions in energy intake over time were mainly due to lower consumption of plain milk, SSBs, and 100% juice.

In 2015, SSBs were the leading source of energy intake from beverages (36% of calories), while juice accounted for 10% of beverage calories. The free sugars in these products increase risk for excess weight gain, type 2 diabetes, and cardiovascular disease [12, 13, 35, 36], warranting continued efforts to reduce Canadians’ exposure. As an approximation, 132 kcal from these sugary drinks equates with 7% of a 2000 kcal diet [47]. This suggests that Canadians’ average free sugars intake from sugary drinks alone may exceed the WHO’s recommendation to limit the consumption of free sugars to no more than 10% of total energy intake, with further benefits from reducing to less than 5% [34]. Although this study did not examine the proportions of Canadians who exceeded these thresholds, given observed mean consumption, they are unlikely to be trivial. Within the SSB category, reductions in intake between 2004 and 2015 were primarily driven by reduced intake of caloric carbonated soft drinks and fruit drinks. These reductions canceled out increases in intake of other SSB types, such as energy drinks, flavoured waters, and sweetened flavoured milk. Milk products containing free sugars (e.g. chocolate milk) are commonplace in the Canadian food supply [6]. Based on analysis of proprietary data sources [48] for Canada and trends in the US and UK [19–22], the declines in Canadians’ sugary drink intake were expected and may be due to shifting consumer preferences, increased public concern related to the health implications of SSBs, as well as public health interventions that have discouraged consumption of sugary drinks [49]. Decreases observed between 2004 and 2015 were preceded by decades of increasing soft drink and fruit juice consumption, as reported using data from Canadian household budget surveys between 1938 and 2011 [50]. There remains substantial scope for reducing Canadians’ sugary drink consumption through complementary measures such as reformulation, sugary drink taxation, and polices to reduce their availability [26]. Indeed, Canada’s Dietary Guidelines emphasize that sugary drinks and foods should not be consumed regularly and indicate that foods and beverages offered in publicly-funded institutions should align with this guidance, providing an actionable step to begin shifting food environments [51].

As in many other Western countries [5], alcohol consumption represents a significant source of energy intake among Canadians. Alcohol contributed one in four calories from beverages, the second highest proportion. The contribution of beverage calories from alcohol was even higher among adults. Males consumed alcohol at a higher volume, whereas based on calories per millilitre, females consumed comparatively more energy-dense alcoholic beverages. Unlike sugary drink categories, alcohol consumption did not decrease from 2004 to 2015, which is concerning given the growing body of evidence on both the acute harms (e.g. risk of physical injury and criminal offences) and longer-term impacts (e.g. alcohol dependence and chronic diseases) of even modest levels of intake [52].

Plain milk (which includes plant-based substitutes) was the third leading contributor of beverage calories (23%). Unlike other analyses of 2004 CCHS-Nut [24, 25], the plain milk category used in this study excluded sugar-sweetened milks containing free sugars (e.g. chocolate milk), which were instead considered within SSBs. Plain milk is less energy dense but consumed in much greater volume (7 times greater) compared with flavoured milks. Compared to all other beverage categories, the greatest reduction from 2004 to 2015, both by volume and calories, was observed for plain milk, consistent with other findings [50].

Although sugary drinks, alcohol, and plain milk were the main sources of calories from beverages, plain water and other unsweetened beverages were the leading contributors by volume (48 and 20%, respectively). Neither beverage category contained products with free sugars. Due to the health benefits of consuming water rather than other beverage types, Canada’s Food Guide and other nutrition guidance recommend water as the best choice for hydration [53, 54]. In contrast to most other beverage categories, intake of plain water increased from 2004 to 2015. Consumers may be using water to compensate for reduced intake of plain milk, SSB, and juice consumption; this substitution may yield health benefits. Diet or light beverages, which may also be considered a possible substitute for higher calorie beverages, continued to represent the smallest volume of beverages consumed in 2015 at only 2%. The low consumption of these products may reflect Canadians’ discomfort with non-nutritive sweeteners, including artificial sweeteners [55], and their lesser presence in the Western food supply compared to products with added sugars [56]. Despite the reported introduction of a wider range of diet and low calorie beverages to Canadian consumers [57], sampled Canadians reported consuming a smaller volume of these products in 2015 than in 2004. This is contrary to trends in the US that show increased consumption [19, 58].

Similar to previous studies in Canada [24, 25], there were differences observed in 2015 beverage consumption according to sex and age. Some differences may not be sufficient to be of importance for nutrition and health, but the overall patterns suggest some important disparities. In 2015, males reported significantly higher consumption compared with females for all beverage categories except water (though males generally have higher consumption of energy overall). The highest sugary drink consumption continues to be seen among children and young people. Young children and young adults were the highest consumers of 100% juice and SSBs, respectively, while the second highest consumption of these categories was among older children. Those of Indigenous ethnicity consumed the highest level of SSBs. Those of white ethnicity, who made up 72% of the weighted sample, consumed the most alcohol. As reported for the US and Australia [59, 60], alcohol consumption was greatest among the highest income quartile. Across the provinces, consumption varied and it is unclear whether differences may be attributable to provincial-level policies. For example, higher alcohol consumption was observed in Quebec, which has a less restrictive alcohol control system compared with other provinces [61]. Future comparisons of this nature may be helpful for identifying the implications of policy interventions that may differ by jurisdiction. Few differences were observed in beverage consumption by income quartile, similar to previous research [37, 38, 62, 63].

Previous studies show few associations between BMI and beverage intake [39, 40]. In this study, the 2015 intake of 100% juice was lowest and, as reported in the US [64], consumption of diet beverages was highest among persons with obesity compared with other BMI groups. This is possibly due to misreporting of dietary intake that is associated with body weight status [65] and that complicates comparisons in relation to BMI. Additionally, it is possible that any given individual with overweight or obesity may be attempting to lose weight on a given day, potentially leading to the consumption of water or diet beverages instead of sugary drinks. The association between higher 100% juice consumption and lower BMI may also be due to confounding with other healthy behaviours. Despite the presence of free sugars in 100% juice, there are widespread misperceptions that it is health-promoting [65].

This study’s findings should be interpreted in light of multiple considerations. Dietary intake data are affected by measurement error, both random and systematic. The study’s research questions focused on population-level means, which did not require adjustments for within-person random error (i.e., day-to-day variation in intake) [31, 32, 66]. Systematic error has been shown to be less substantial in data from 24-h dietary recalls compared to that from other dietary assessment tools, such as food frequency questionnaires [67]. Nonetheless, evidence indicates that underestimation of energy intake occurred in both survey years [68, 69]; thus, the beverage intake reported may underestimate true intake levels. Additionally, underreporting appears to be higher in the 2015 survey [69]. This differential misreporting by survey year, which has been reported for other jurisdictions [70–72], may have contributed to observed declines in intake and suggests that the findings of the trend analyses should be cautiously interpreted. No standard adjustment currently exists for correcting underreporting [73]. CCHS is not representative of the entire Canadian population: the sampling frame did not include Canada’s three territories and persons living on reserve and other Indigenous peoples’ settlements. Despite these limitations, the CCHS data represent the most robust estimates of beverage intake in Canada. Socio-economic status is a complex construct and is not adequately represented using income alone [74]. Finally, when examining alcohol consumption, the current study did not report consumption according to the ‘standard drinks’ format used in Canada’s alcohol guidelines [75].

## Conclusions

Canadians reported consuming less 100% juice, SSBs, plain milk, and other beverages in 2015 compared to 2004, with an apparent shift toward plain water. Differential consumption was seen across socio-demographic groups; however, at the population level, the consumption of sugary drinks and alcohol remains high. Given misreporting of dietary intake, the estimated consumption may be underestimated, with possible increases in underestimation over time contributing to observed declines in consumption [69]. The findings underscore the need for policies to further reduce the consumption of sugary and alcoholic beverages, as well as calories from beverages.

## Additional file

**Additional file 1.** Beverage sub-categories and further results.

## Data Availability

The authors do not own the data underlying this study. CCHS-Nut data is available through Statistics Canada’s Research Data Centres once researchers have met the required criteria.

## Abbreviations

95% CI: 95% confidence interval
BMI: Body-mass index
CCHS-Nut: Canadian Community Health Survey–Nutrition
DK: Don’t know
kcal: kilocalorie
ml: millilitre
NF: Newfoundland and Labrador
non. sig.: Statistically non-significant
NS: Not stated
PEI: Prince Edward Island
SSB: Sugar-sweetened beverage
WHO: World Health Organization
