# Supplementary material for: Beverage consumption and energy intake among Canadians: analyses of 2004 and 2015 national dietary intake data
Source: Nutr J. 2019 Oct 18;18:60. doi: 10.1186/s12937-019-0488-5 (PMC6800499; doi:10.1186/s12937-019-0488-5)
Supplement: Supplementary file 1 — Additional file 1. Beverage sub-categories and further results. [file 12937_2019_488_MOESM1_ESM.docx]

**SUPPLEMENTARY FILE**

Supplementary file for “Beverage consumption and energy intake among Canadians: analyses of 2004 and 2015 national dietary intake data” by Amanda C. Jones, Sharon I. Kirkpatrick, David Hammond

Appendix A: Beverage Sub-Categories 2

Supplementary File Table 1. Beverage sub-categories 2

Appendix B: 2015 Pairwise comparisons 3

Supplementary File Table 2. Pairwise comparisons of socio-demographic variables for daily per capita plain water consumption in 2015 (N=20176) 3

Supplementary File Table 3. Pairwise comparisons of socio-demographic variables for daily per capita ‘other unsweetened beverages’ consumption in 2015 (N=20176) 6

Supplementary File Table 4. Pairwise comparisons of socio-demographic variables for daily per capita SSB consumption consumption in 2015 (N=20176) 9

Supplementary File Table 5. Pairwise comparisons of socio-demographic variables for daily per capita plain milk consumption in 2015 (N=20176) 12

Supplementary File Table 6. Pairwise comparisons of socio-demographic variables for daily per capita alcoholic beverage consumption in 2015 (N=20176) 15

Supplementary File Table 7. Pairwise comparisons of socio-demographic variables for daily per capita 100% juice consumption in 2015 (N=20176) 18

Supplementary File Table 8. Pairwise comparisons of socio-demographic variables for daily per capita diet or light beverage consumption in 2015 (N=20176) 21

Appendix C: 2004 Beverage consumption 24

Supplementary File Table 9. Daily per capita beverage volume intake (ml) from beverage categories in 2004, by socio-demographic variable (N=33463) 24

Supplementary File Table 10. Daily per capita energy intake (kcal) from beverage categories in 2004, by socio-demographic variable (N=34,463) 26

Appendix D: 2004 Additional analyses of national and age-sex consumption 28

Supplementary File Table 11. Daily per capita volume intake (ml) from beverage categories and sub-categories in 2004, by all and age-sex group 28

Supplementary File Table 12. Daily per capita energy intake (kcal) from beverage categories and sub-categories in 2004, by all and age-sex group 30

Appendix E: 2015 Additional analyses of national and age-sex consumption 32

Supplementary File Table 13. Daily per capita volume intake (ml) from beverage categories and sub-categories in 2015, by all and age-sex group 32

Supplementary File Table 14. Daily per capita energy intake (kcal) from beverage categories and sub-categories in 2015, by all and age-sex group 34

# Appendix A: Beverage Sub-Categories

## Supplementary File Table 1. Beverage sub-categories

| **Beverage category** | **Beverage sub-category** |
| --- | --- |
| **Plain water** | Other water (tap, well) |
|  | Bottled water |
| **Other unsweetened beverages** | Unsweetened coffee |
|  | Unsweetened tea |
|  | Club soda and other (unsweetened hot chocolate, unsweetened flavoured milk) |
| **Sugar-sweetened beverages (SSBs)** | Regular carbonated soft drinks |
|  | Regular fruit drinks |
|  | Sugar-sweetened milk |
|  | Tea pre-sweetened with sugar |
|  | Regular sports drinks |
|  | Hot chocolate pre-sweetened with sugar |
|  | Smoothies |
|  | Coffee pre-sweetened with sugar |
|  | Regular meal replacement beverages |
|  | Regular energy drinks |
|  | Flavoured drinkable yogurt |
|  | Regular flavoured water |
|  | Regular protein drinks |
|  | Other SSBs (coffee or tea sweetened by the consumer, homemade hot chocolate prepared from scratch) |
| **Plain milk** | Plain milk (unsweetened, unflavoured milk or substitutes) |
| **Alcoholic beverages** | Beer |
|  | Wine |
|  | Cocktails |
|  | Spirits |
|  | Coolers |
|  | Liqueur |
| **100% juice** | 100% juice |
| **Diet or light beverages** | Diet or light carbonated soft drinks |
|  | Diet or light fruit drinks |
|  | Diet or light meal replacement beverages |
|  | Diet or light hot chocolate |
|  | Diet or light coffee |
|  | Diet or light sports drinks |
|  | Diet or light energy drinks |
|  | Diet or light flavoured water |
|  | Diet or light tea |
|  | Diet or light protein drinks |

#

# Appendix B: 2015 Pairwise comparisons

## Supplementary File Table 2. Pairwise comparisons of socio-demographic variables for daily per capita plain water consumption in 2015 (N=20176)

|  | **Plain water** | | |
| --- | --- | --- | --- |
|  | **Mean volume in ml** | | |
|  | **Estimate** | **(95% CI)** | **Adjusted  p-value** |
| **Model statistics** | F_(26,20176)_=57.08 | | |
| **Sex** | p=0.6492 | | |
| **Age (years)** | **p<.0001** | | |
| 1-8 vs 9-18 | **-365.3** | **(-411.8, -318.9)** | **<.0001** |
| 1-8 vs 19-30 | **-631.0** | **(-750.0, -512.1)** | **<.0001** |
| 1-8 vs 31-50 | **-501.9** | **(-560.2, -443.6)** | **<.0001** |
| 1-8 vs 51+ | **-303.6** | **(-348.4, -258.7)** | **<.0001** |
| 9-18 vs 19-30 | **-265.7** | **(-382.0, -149.4)** | **<.0001** |
| 9-18 vs 31-50 | **-136.5** | **(-197.7, -75.4)** | **<.0001** |
| 9-18 vs 51+ | **61.8** | **(13.7, 109.8)** | **0.0118** |
| 19-30 vs 31-50 | 129.2 | (2.4, 255.9) | 0.0458 |
| 19-30 vs 51+ | **327.5** | **(206.6, 448.3)** | **<.0001** |
| 31-50 vs 51+ | **198.3** | **(139.4, 257.2)** | **<.0001** |
| **Ethnicity** | **p=0.0100** | | |
| White only vs Chinese only | **150.0** | **(68.1, 231.9)** | **0.0004** |
| White only vs South Asian only | 7.1 | (-80.8, 95.1) | 0.8736 |
| White only vs Black only | 78.8 | (-54.8, 212.5) | 0.2471 |
| White only vs Indigenous inclusive | 15.5 | (-88.5, 119.5) | 0.7697 |
| White only vs Mixed/other/NS/missing | -25.1 | (-117.1, 66.8) | 0.5917 |
| Chinese only vs South Asian only | **-142.9** | **(-250.9, -34.9)** | **0.0096** |
| Chinese only vs Black only | -71.2 | (-220.7, 78.4) | 0.3503 |
| Chinese only vs Indigenous inclusive | -134.5 | (-259.7, -9.3) | 0.0354 |
| Chinese only vs Mixed/other/NS/missing | **-175.1** | **(-288.7, -61.5)** | **0.0026** |
| South Asian only vs Black only | 71.7 | (-72.8, 216.2) | 0.3301 |
| South Asian only vs Indigenous inclusive | 8.4 | (-119.3, 136.1) | 0.8975 |
| South Asian only vs Mixed/other/NS/missing | -32.2 | (-146.5, 82.0) | 0.5795 |
| Black only vs Indigenous inclusive | -63.3 | (-230.1, 103.4) | 0.4560 |
| Black only vs Mixed/other/NS/missing | -103.9 | (-256.5, 48.6) | 0.1813 |
| Indigenous inclusive vs Mixed/other/NS/missing | -40.6 | (-178.6, 97.4) | 0.5633 |
| **Income (1=lowest income; 4=highest income)** | **p<.0001** | | |
| Quartile 1 vs Quartile 2 | -72.6 | (-150.8, 5.5) | 0.0683 |
| Quartile 1 vs Quartile 3 | **-144.5** | **(-224.1, -64.9)** | **0.0004** |
| Quartile 1 vs Quartile 4 | **-250.5** | **(-331.6, -169.4)** | **<.0001** |
| Quartile 1 vs Not reported | -84.2 | (-160.1, -8.4) | 0.0295 |
| Quartile 2 vs Quartile 3 | -71.9 | (-150.3, 6.5) | 0.0724 |
| Quartile 2 vs Quartile 4 | **-177.9** | **(-265.0, -90.7)** | **<.0001** |
| Quartile 2 vs Not reported | -11.6 | (-92.8, 69.6) | 0.7795 |
| Quartile 3 vs Quartile 4 | **-106.0** | **(-191.3, -20.7)** | **0.0150** |
| Quartile 3 vs Not reported | 60.3 | (-19.5, 140.1) | 0.1382 |
| Quartile 4 vs Not reported | **166.3** | **(82.7, 249.8)** | **0.0001** |
| **Province** | **p<.0001** | | |
| NL vs PEI | -54.3 | (-156.8, 48.1) | 0.2978 |
| NL vs Nova Scotia | **-199.8** | **(-315.8, -83.8)** | **0.0008** |
| NL vs New Brunswick | **-196.5** | **(-313.0, -80.0)** | **0.0010** |
| NL vs Quebec | **-185.2** | **(-271.1, -99.4)** | **<.0001** |
| NL vs Ontario | **-207.7** | **(-291.6, -123.9)** | **<.0001** |
| NL vs Manitoba | **-263.5** | **(-375.2, -151.9)** | **<.0001** |
| NL vs Saskatchewan | **-221.9** | **(-333.1, -110.7)** | **0.0001** |
| NL vs Alberta | **-355.5** | **(-462.2, -248.9)** | **<.0001** |
| NL vs British Columbia | **-353.4** | **(-463.6, -243.1)** | **<.0001** |
| PEI vs Nova Scotia | **-145.5** | **(-259.8, -31.2)** | **0.0127** |
| PEI vs New Brunswick | **-142.2** | **(-248.4, -36.0)** | **0.0088** |
| PEI vs Quebec | **-130.9** | **(-217.0, -44.8)** | **0.0029** |
| PEI vs Ontario | **-153.4** | **(-234.7, -72.1)** | **0.0002** |
| PEI vs Manitoba | **-209.2** | **(-324.3, -94.1)** | **0.0004** |
| PEI vs Saskatchewan | **-167.6** | **(-274.6, -60.6)** | **0.0022** |
| PEI vs Alberta | **-301.2** | **(-410.4, -192.0)** | **<.0001** |
| PEI vs British Columbia | **-299.0** | **(-411.4, -186.7)** | **<.0001** |
| Nova Scotia vs New Brunswick | 3.3 | (-124.5, 131.1) | 0.9598 |
| Nova Scotia vs Quebec | 14.6 | (-88.4, 117.6) | 0.7811 |
| Nova Scotia vs Ontario | -7.9 | (-112.0, 96.2) | 0.8810 |
| Nova Scotia vs Manitoba | -63.7 | (-199.7, 72.2) | 0.3575 |
| Nova Scotia vs Saskatchewan | -22.1 | (-147.0, 102.8) | 0.7285 |
| Nova Scotia vs Alberta | **-155.7** | **(-281.3, -30.2)** | **0.0152** |
| Nova Scotia vs British Columbia | **-153.6** | **(-279.6, -27.5)** | **0.0170** |
| New Brunswick vs Quebec | 11.3 | (-89.6, 112.2) | 0.8261 |
| New Brunswick vs Ontario | -11.2 | (-110.5, 88.1) | 0.8245 |
| New Brunswick vs Manitoba | -67.0 | (-192.7, 58.7) | 0.2955 |
| New Brunswick vs Saskatchewan | -25.4 | (-144.0, 93.2) | 0.6745 |
| New Brunswick vs Alberta | **-159.0** | **(-278.0, -40.0)** | **0.0089** |
| New Brunswick vs British Columbia | **-156.8** | **(-272.7, -41.0)** | **0.0081** |
| Quebec vs Ontario | -22.5 | (-93.8, 48.8) | 0.5352 |
| Quebec vs Manitoba | -78.3 | (-184.0, 27.4) | 0.1462 |
| Quebec vs Saskatchewan | -36.7 | (-134.9, 61.6) | 0.4639 |
| Quebec vs Alberta | **-170.3** | **(-268.1, -72.5)** | **0.0007** |
| Quebec vs British Columbia | **-168.1** | **(-268.9, -67.4)** | **0.0011** |
| Ontario vs Manitoba | -55.8 | (-154.6, 43.0) | 0.2676 |
| Ontario vs Saskatchewan | -14.1 | (-112.3, 84.0) | 0.7771 |
| Ontario vs Alberta | **-147.8** | **(-243.6, -51.9)** | **0.0026** |
| Ontario vs British Columbia | **-145.6** | **(-243.2, -48.1)** | **0.0035** |
| Manitoba vs Saskatchewan | 41.7 | (-78.8, 162.1) | 0.4971 |
| Manitoba vs Alberta | -92.0 | (-207.8, 23.8) | 0.1192 |
| Manitoba vs British Columbia | -89.8 | (-212.4, 32.8) | 0.1507 |
| Saskatchewan vs Alberta | **-133.6** | **(-251.3, -16.0)** | **0.0261** |
| Saskatchewan vs British Columbia | -131.5 | (-249.9, -13.1) | 0.0296 |
| Alberta vs British Columbia | 2.2 | (-113.0, 117.4) | 0.9706 |
| **BMI category** | **p<.0001** | | |
| Underweight/normal vs Overweight | -124.1 | **(-190.1, -58.1)** | **0.0002** |
| Underweight/normal vs Obese | -220.5 | **(-299.9, -141.1)** | **<.0001** |
| Underweight/normal vs DK/Refusal/NS | -86.2 | **(-149.9, -22.6)** | **0.0080** |
| Overweight vs Obese | -96.4 | (-188.6, -4.2) | 0.0405 |
| Overweight vs DK/Refusal/NS | 37.8 | (-35.5, 111.2) | 0.3111 |
| Obese vs DK/Refusal/NS | **134.3** | **(46.5, 222.0)** | **0.0028** |

No model was constructed for plain water as this beverage category contained no energy. Model statistics are for separate linear models using generalized least squares regression with F-Wald test of beverage volume and energy, with covariates sex, age, ethnicity, income, province, and BMI category, followed by Student t-test for pairwise comparisons. Pairwise contrasts not shown for covariates that did not have a significant overall effect. Values in bold font represent findings significant after post hoc adjustment using Benjamini-Hochberg procedure.

Abbreviations: 95% CI, 95% confidence interval; BMI, body-mass index; DK, don’t know; kcal, kilocalorie; NF, Newfoundland and Labrador; NS, not stated; PEI, Prince Edward Island

## Supplementary File Table 3. Pairwise comparisons of socio-demographic variables for daily per capita ‘other unsweetened beverages’ consumption in 2015 (N=20176)

|  | **Other unsweetened beverages** | | | | | |
| --- | --- | --- | --- | --- | --- | --- |
|  | **Mean volume in ml** | | | **Mean energy in kcal** | | |
|  | **Estimate** | **(95% CI)** | **Adjusted  p-value** | **Estimate** | **(95% CI)** | **Adjusted  p-value** |
| **Model statistics** | F_(26,20176)_=170.71 | | | F_(26,20176)_=15.46 | | |
| **Sex** | **p=0.0052** | | | p=0.4423 | | |
| Male vs female | **33.9** | **(10.2, 57.7)** | **0.0052** | - | - | - |
| **Age (years)** | **p<.0001** | | | **p<.0001** | | |
| 1-8 vs 9-18 | **-46.1** | **(-55.8, -36.5)** | **<.0001** | -1.2 | (-2.2, -0.1) | 0.0308 |
| 1-8 vs 19-30 | **-284.9** | **(-322.0, -247.8)** | **<.0001** | **-5.2** | **(-7.4, -2.9)** | **<.0001** |
| 1-8 vs 31-50 | **-443.1** | **(-468.7, -417.5)** | **<.0001** | **-9.0** | **(-12.4, -5.5)** | **<.0001** |
| 1-8 vs 51+ | **-484.6** | **(-503.5, -465.8)** | **<.0001** | **-5.9** | **(-7.0, -4.8)** | **<.0001** |
| 9-18 vs 19-30 | **-238.8** | **(-275.6, -202.0)** | **<.0001** | **-4.0** | **(-6.3, -1.8)** | **0.0004** |
| 9-18 vs 31-50 | **-397.0** | **(-422.7, -371.3)** | **<.0001** | **-7.8** | **(-10.8, -4.8)** | **<.0001** |
| 9-18 vs 51+ | **-438.5** | **(-459.2, -417.9)** | **<.0001** | **-4.7** | **(-5.9, -3.6)** | **<.0001** |
| 19-30 vs 31-50 | **-158.2** | **(-201.3, -115.1)** | **<.0001** | -3.8 | (-7.3, -0.4) | 0.0298 |
| 19-30 vs 51+ | **-199.7** | **(-239.7, -159.7)** | **<.0001** | -0.7 | (-2.9, 1.5) | 0.5162 |
| 31-50 vs 51+ | **-41.5** | **(-71.9, -11.1)** | **0.0075** | 3.1 | (0.1, 6.1) | 0.0403 |
| **Ethnicity** | **p<.0001** | | | p=0.0975 | | |
| White only vs Chinese only | 40.3 | (-22.8, 103.3) | 0.2101 | - | - | - |
| White only vs South Asian only | **124.3** | **(89.3, 159.3)** | **<.0001** | - | - | - |
| White only vs Black only | **186.4** | **(148.0, 224.8)** | **<.0001** | - | - | - |
| White only vs Indigenous inclusive | 24.8 | (-28.8, 78.5) | 0.3638 | - | - | - |
| White only vs Mixed/other/NS/missing | **51.3** | **(9.4, 93.2)** | **0.0166** | - | - | - |
| Chinese only vs South Asian only | **84.0** | **(16.5, 151.6)** | **0.0149** | - | - | - |
| Chinese only vs Black only | **146.1** | **(75.2, 217.0)** | **<.0001** | - | - | - |
| Chinese only vs Indigenous inclusive | -15.4 | (-95.3, 64.5) | 0.7042 | - | - | - |
| Chinese only vs Mixed/other/NS/missing | 11.0 | (-62.6, 84.6) | 0.7693 | - | - | - |
| South Asian only vs Black only | **62.1** | **(20.5, 103.6)** | **0.0035** | - | - | - |
| South Asian only vs Indigenous inclusive | **-99.5** | **(-159.5, -39.5)** | **0.0012** | - | - | - |
| South Asian only vs Mixed/other/NS/missing | **-73.0** | **(-120.5, -25.6)** | **0.0026** | - | - | - |
| Black only vs Indigenous inclusive | **-161.5** | **(-221.0, -102.0)** | **<.0001** | - | - | - |
| Black only vs Mixed/other/NS/missing | **-135.1** | **(-186.8, -83.4)** | **<.0001** | - | - | - |
| Indigenous inclusive vs Mixed/other/NS/missing | 26.4 | (-38.9, 91.8) | 0.4269 | - | - | - |
| **Income (1=lowest income; 4=highest income)** | **p=0.0213** | | | p=0.3040 | | |
| Quartile 1 vs Quartile 2 | 37.6 | (5.6, 69.6) | 0.0215 | 2.7 | (-1.4, 6.8) | 0.1926 |
| Quartile 1 vs Quartile 3 | -7.1 | (-43.4, 29.2) | 0.7000 | 1.8 | (-2.6, 6.1) | 0.4327 |
| Quartile 1 vs Quartile 4 | -9.0 | (-50.8, 32.7) | 0.6710 | 1.0 | (-2.7, 4.7) | 0.5835 |
| Quartile 1 vs Not reported | 17.9 | (-16.8, 52.5) | 0.3115 | 1.8 | (-2.8, 6.3) | 0.4437 |
| Quartile 2 vs Quartile 3 | **-44.7** | **(-76.4, -13.0)** | **0.0057** | -0.9 | (-3.0, 1.1) | 0.3652 |
| Quartile 2 vs Quartile 4 | **-46.6** | **(-83.4, -9.8)** | **0.0131** | -1.7 | (-3.6, 0.3) | 0.0884 |
| Quartile 2 vs Not reported | -19.7 | (-49.9, 10.4) | 0.1996 | -0.9 | (-2.4, 0.6) | 0.2333 |
| Quartile 3 vs Quartile 4 | -1.9 | (-40.4, 36.6) | 0.9226 | -0.7 | (-3.4, 1.9) | 0.5917 |
| Quartile 3 vs Not reported | 25.0 | (-9.9, 59.8) | 0.1596 | 0.02 | (-2.40, 2.44) | 0.9864 |
| Quartile 4 vs Not reported | 26.9 | (-13.9, 67.6) | 0.1956 | 0.7 | (-1.5, 3.0) | 0.5126 |
| **Province** | **p=0.0054** | | | **p<.0001** | | |
| NL vs PEI | -25.6 | (-82.4, 31.2) | 0.3765 | -0.001 | (-1.671, 1.668) | 0.9988 |
| NL vs Nova Scotia | -10.2 | (-67.2, 46.7) | 0.7246 | 0.4 | (-0.9, 1.6) | 0.5862 |
| NL vs New Brunswick | -11.5 | (-74.1, 51.2) | 0.7192 | 0.2 | (-1.6, 2.0) | 0.8282 |
| NL vs Quebec | 19.2 | (-35.1, 73.5) | 0.4881 | **-6.1** | **(-9.9, -2.2)** | **0.0022** |
| NL vs Ontario | -20.6 | (-72.9, 31.6) | 0.4389 | -0.2 | (-2.2, 1.8) | 0.8667 |
| NL vs Manitoba | -39.1 | (-95.2, 17.1) | 0.1724 | -0.3 | (-2.1, 1.5) | 0.7552 |
| NL vs Saskatchewan | **-92.8** | **(-158.0, -27.5)** | **0.0054** | 1.1 | (-0.2, 2.3) | 0.0864 |
| NL vs Alberta | -26.2 | (-85.1, 32.7) | 0.3825 | -1.2 | (-3.7, 1.3) | 0.3379 |
| NL vs British Columbia | -41.3 | (-94.0, 11.5) | 0.1251 | -2.2 | (-4.6, 0.1) | 0.0645 |
| PEI vs Nova Scotia | 15.4 | (-31.2, 62.0) | 0.5169 | 0.4 | (-1.4, 2.1) | 0.6905 |
| PEI vs New Brunswick | 14.1 | (-41.9, 70.2) | 0.6203 | 0.2 | (-2.0, 2.4) | 0.8586 |
| PEI vs Quebec | 44.8 | (1.7, 87.9) | 0.0416 | **-6.1** | **(-10.0, -2.1)** | **0.0029** |
| PEI vs Ontario | 5.0 | (-35.9, 45.9) | 0.8103 | -0.2 | (-2.5, 2.2) | 0.8869 |
| PEI vs Manitoba | -13.5 | (-61.7, 34.7) | 0.5835 | -0.3 | (-2.7, 2.1) | 0.8099 |
| PEI vs Saskatchewan | -67.2 | (-128.0, -6.4) | 0.0304 | 1.1 | (-0.7, 2.8) | 0.2324 |
| PEI vs Alberta | -0.6 | (-50.4, 49.1) | 0.9808 | -1.2 | (-3.9, 1.5) | 0.3881 |
| PEI vs British Columbia | -15.6 | (-61.2, 29.9) | 0.5003 | -2.2 | (-5.0, 0.5) | 0.1091 |
| Nova Scotia vs New Brunswick | -1.3 | (-53.9, 51.4) | 0.9628 | -0.2 | (-2.0, 1.7) | 0.8643 |
| Nova Scotia vs Quebec | 29.4 | (-13.1, 71.9) | 0.1743 | **-6.4** | **(-10.4, -2.5)** | **0.0015** |
| Nova Scotia vs Ontario | -10.4 | (-50.6, 29.8) | 0.6115 | -0.5 | (-2.4, 1.4) | 0.5866 |
| Nova Scotia vs Manitoba | -28.9 | (-77.0, 19.3) | 0.2395 | -0.7 | (-2.5, 1.1) | 0.4782 |
| Nova Scotia vs Saskatchewan | -82.6 | (-139, -26.1) | 0.0042 | 0.7 | (-0.5, 2.0) | 0.2613 |
| Nova Scotia vs Alberta | -16.0 | (-64.5, 32.5) | 0.5173 | -1.6 | (-4.0, 0.9) | 0.2070 |
| Nova Scotia vs British Columbia | -31.0 | (-74.8, 12.8) | 0.1644 | -2.6 | (-4.9, -0.3) | 0.0270 |
| New Brunswick vs Quebec | 30.7 | (-18.2, 79.5) | 0.2183 | **-6.3** | **(-10.7, -1.9)** | **0.0054** |
| New Brunswick vs Ontario | -9.1 | (-55.5, 37.2) | 0.6987 | -0.4 | (-2.6, 1.8) | 0.7399 |
| New Brunswick vs Manitoba | -27.6 | (-85.5, 30.3) | 0.3491 | -0.5 | (-2.7, 1.7) | 0.6627 |
| New Brunswick vs Saskatchewan | **-81.3** | **(-145.0, -17.6)** | **0.0125** | 0.9 | (-0.9, 2.6) | 0.3343 |
| New Brunswick vs Alberta | -14.7 | (-71.1, 41.6) | 0.6073 | -1.4 | (-4.1, 1.3) | 0.3066 |
| New Brunswick vs British Columbia | -29.8 | (-80.0, 20.4) | 0.2442 | -2.4 | (-5.0, 0.1) | 0.0639 |
| Quebec vs Ontario | -39.8 | (-74.1, -5.4) | 0.0233 | 5.9 | (0.9, 10.9) | 0.0210 |
| Quebec vs Manitoba | **-58.3** | **(-102.2, -14.3)** | **0.0094** | 5.8 | (1.3, 10.3) | 0.0120 |
| Quebec vs Saskatchewan | **-112.0** | **(-166.5, -57.4)** | **<.0001** | **7.1** | **(3.0, 11.3)** | **0.0008** |
| Quebec vs Alberta | -45.4 | (-89.6, -1.2) | 0.0441 | 4.9 | (0.1, 9.6) | 0.0469 |
| Quebec vs British Columbia | **-60.4** | **(-100.4, -20.5)** | **0.0031** | 3.8 | (-1.3, 8.9) | 0.1431 |
| Ontario vs Manitoba | -18.5 | (-59.1, 22.2) | 0.3729 | -0.1 | (-2.3, 2.0) | 0.9106 |
| Ontario vs Saskatchewan | **-72.2** | **(-124.0, -20.4)** | **0.0064** | 1.2 | (-0.5, 3.0) | 0.1631 |
| Ontario vs Alberta | -5.6 | (-47.8, 36.6) | 0.7939 | -1.0 | (-3.6, 1.5) | 0.4294 |
| Ontario vs British Columbia | -20.6 | (-56.7, 15.4) | 0.2606 | -2.1 | (-4.5, 0.4) | 0.0967 |
| Manitoba vs Saskatchewan | -53.7 | (-113.1, 5.7) | 0.0762 | 1.4 | (-0.4, 3.1) | 0.1189 |
| Manitoba vs Alberta | 12.9 | (-38.0, 63.7) | 0.6197 | -0.9 | (-3.7, 1.9) | 0.5221 |
| Manitoba vs British Columbia | -2.2 | (-48.6, 44.2) | 0.9264 | -2.0 | (-4.4, 0.4) | 0.1107 |
| Saskatchewan vs Alberta | 66.6 | (7.1, 126.0) | 0.0284 | -2.3 | (-4.6, 0.1) | 0.0580 |
| Saskatchewan vs British Columbia | 51.5 | (-5.2, 108.2) | 0.0747 | **-3.3** | **(-5.5, -1.1)** | **0.0033** |
| Alberta vs British Columbia | -15.0 | (-59.6, 29.5) | 0.5071 | -1.0 | (-4.0, 1.9) | 0.4907 |
| **BMI category** | p=0.7782 | | | p=0.2642 | | |

Model statistics are for separate linear models using generalized least squares regression with F-Wald test of beverage volume and energy, with covariates sex, age, ethnicity, income, province, and BMI category, followed by Student t-test for pairwise comparisons. Pairwise contrasts not shown for covariates that did not have a significant overall effect. Values in bold font represent findings significant after post hoc adjustment using Benjamini-Hochberg procedure.

Abbreviations: 95% CI, 95% confidence interval; BMI, body-mass index; DK, don’t know; kcal, kilocalorie; NF, Newfoundland and Labrador; NS, not stated; PEI, Prince Edward Island

## Supplementary File Table 4. Pairwise comparisons of socio-demographic variables for daily per capita SSB consumption consumption in 2015 (N=20176)

|  | **SSBs** | | | | | |
| --- | --- | --- | --- | --- | --- | --- |
|  | **Mean volume in ml** | | | **Mean energy in kcal** | | |
|  | **Estimate** | **(95% CI)** | **Adjusted  p-value** | **Estimate** | **(95% CI)** | **Adjusted  p-value** |
| **Model statistics** | F_(26,20176)_=57.06 | | | F_(26,20176)_=55.35 | | |
| **Sex** | **p<.0001** | | | **p<.0001** | | |
| Male vs female | **82.1** | **(62.8, 101.4)** | **<.0001** | **40.2** | **(30.2, 50.3)** | **<.0001** |
| **Age (years)** | **p<.0001** | | | **p<.0001** | | |
| 1-8 vs 9-18 | **-172.3** | **(-196.2, -148.3)** | **<.0001** | **-77.1** | **(-89.9, -64.4)** | **<.0001** |
| 1-8 vs 19-30 | **-192.8** | **(-243.4, -142.1)** | **<.0001** | **-89.4** | **(-116.6, -62.3)** | **<.0001** |
| 1-8 vs 31-50 | **-88.8** | **(-111.8, -65.9)** | **<.0001** | **-29.9** | **(-43.6, -16.3)** | **<.0001** |
| 1-8 vs 51+ | -9.5 | (-28.0, 9.1) | 0.3163 | 8.9 | (-1.8, 19.5) | 0.1023 |
| 9-18 vs 19-30 | -20.5 | (-74.6, 33.6) | 0.4572 | -12.3 | (-41.2, 16.7) | 0.4048 |
| 9-18 vs 31-50 | **83.4** | **(57.1, 109.7)** | **<.0001** | **47.2** | **(33.1, 61.4)** | **<.0001** |
| 9-18 vs 51+ | **162.8** | **(137.8, 187.7)** | **<.0001** | **86.0** | **(72.8, 99.2)** | **<.0001** |
| 19-30 vs 31-50 | **103.9** | **(50.9, 156.9)** | **0.0001** | **59.5** | **(29.8, 89.2)** | **<.0001** |
| 19-30 vs 51+ | **183.3** | **(132.2, 234.4)** | **<.0001** | **98.3** | **(70.6, 126.0)** | **<.0001** |
| 31-50 vs 51+ | **79.4** | **(56.1, 102.6)** | **<.0001** | **38.8** | **(26.8, 50.8)** | **<.0001** |
| **Ethnicity** | **p<.0001** | | | **p<.0001** | | |
| White only vs Chinese only | **100.6** | **(69.9, 131.2)** | **<.0001** | **53.6** | **(38.9, 68.3)** | **<.0001** |
| White only vs South Asian only | 43.7 | (-0.6, 87.9) | 0.0531 | 15.1 | (-12.4, 42.5) | 0.2808 |
| White only vs Black only | 14.6 | (-28.2, 57.5) | 0.5023 | 6.9 | (-15.4, 29.3) | 0.5413 |
| White only vs Indigenous inclusive | -69.1 | (-133.5, -4.7) | 0.0356 | -29.0 | (-59.8, 1.7) | 0.0639 |
| White only vs Mixed/other/NS/missing | 21.7 | (-9.6, 52.9) | 0.1734 | 4.8 | (-19.6, 29.1) | 0.6993 |
| Chinese only vs South Asian only | **-56.9** | **(-104.5, -9.4)** | **0.0189** | **-38.5** | **(-65.3, -11.8)** | **0.0048** |
| Chinese only vs Black only | **-86.0** | **(-135.1, -36.8)** | **0.0006** | **-46.7** | **(-70.5, -22.8)** | **0.0001** |
| Chinese only vs Indigenous inclusive | **-169.7** | **(-238.8, -100.6)** | **<.0001** | **-82.6** | **(-115.3, -50.0)** | **<.0001** |
| Chinese only vs Mixed/other/NS/missing | **-78.9** | **(-117.4, -40.4)** | **<.0001** | **-48.8** | **(-75.7, -22.0)** | **0.0004** |
| South Asian only vs Black only | -29.0 | (-83.9, 25.9) | 0.2994 | -8.1 | (-39.4, 23.1) | 0.6098 |
| South Asian only vs Indigenous inclusive | **-112.7** | **(-190.3, -35.1)** | **0.0045** | -44.1 | (-83.8, -4.4) | 0.0294 |
| South Asian only vs Mixed/other/NS/missing | -22.0 | (-65.5, 21.6) | 0.3220 | -10.3 | (-41.7, 21.2) | 0.5208 |
| Black only vs Indigenous inclusive | -83.7 | (-162.7, -4.7) | 0.0380 | -36.0 | (-73.9, 1.9) | 0.0624 |
| Black only vs Mixed/other/NS/missing | 7.1 | (-42.8, 57.0) | 0.7812 | -2.2 | (-34.0, 29.7) | 0.8941 |
| Indigenous inclusive vs Mixed/other/NS/missing | **90.7** | **(21.4, 160.1)** | **0.0105** | 33.8 | (-3.9, 71.6) | 0.0787 |
| **Income (1=lowest income; 4=highest income)** | p=0.2543 | | | p=0.4077 | | |
| **Province** | **p<.0001** | | | **p<.0001** | | |
| NL vs PEI | 30.3 | (-17.2, 77.9) | 0.2110 | 8.6 | (-14.5, 31.7) | 0.4657 |
| NL vs Nova Scotia | 29.7 | (-18.2, 77.5) | 0.2242 | 12.9 | (-10.4, 36.2) | 0.2782 |
| NL vs New Brunswick | 1.8 | (-51.9, 55.4) | 0.9481 | -3.7 | (-30.4, 23.0) | 0.7839 |
| NL vs Quebec | 46.0 | (3.0, 89.0) | 0.0362 | 23.9 | (2.9, 45.0) | 0.0259 |
| NL vs Ontario | 19.3 | (-27.5, 66.1) | 0.4180 | 7.4 | (-15.7, 30.5) | 0.5289 |
| NL vs Manitoba | -0.5 | (-48.5, 47.5) | 0.9829 | 6.2 | (-17.0, 29.3) | 0.6015 |
| NL vs Saskatchewan | 48.7 | (-0.9, 98.2) | 0.0542 | 25.0 | (2.1, 47.9) | 0.0328 |
| NL vs Alberta | -7.4 | (-53.1, 38.3) | 0.7493 | -6.8 | (-31.1, 17.5) | 0.5835 |
| NL vs British Columbia | **78.7** | **(36.6, 120.8)** | **0.0003** | **36.4** | **(15.5, 57.4)** | **0.0007** |
| PEI vs Nova Scotia | -0.7 | (-39.0, 37.7) | 0.9733 | 4.3 | (-14.4, 22.9) | 0.6532 |
| PEI vs New Brunswick | -28.5 | (-70.6, 13.5) | 0.1828 | -12.3 | (-34.1, 9.5) | 0.2673 |
| PEI vs Quebec | 15.7 | (-16.2, 47.6) | 0.3339 | 15.3 | (-0.8, 31.5) | 0.0629 |
| PEI vs Ontario | -11.0 | (-44.7, 22.7) | 0.5211 | -1.2 | (-18.1, 15.7) | 0.8900 |
| PEI vs Manitoba | -30.8 | (-72.1, 10.4) | 0.1427 | -2.4 | (-22.6, 17.7) | 0.8118 |
| PEI vs Saskatchewan | 18.4 | (-24.2, 60.9) | 0.3968 | 16.4 | (-3.6, 36.4) | 0.1086 |
| PEI vs Alberta | -37.7 | (-76.7, 1.2) | 0.0572 | -15.4 | (-36.1, 5.3) | 0.1455 |
| PEI vs British Columbia | **48.4** | **(17.4, 79.3)** | **0.0022** | **27.8** | **(12.2, 43.5)** | **0.0005** |
| Nova Scotia vs New Brunswick | -27.9 | (-73.5, 17.8) | 0.2306 | -16.6 | (-39.3, 6.2) | 0.1524 |
| Nova Scotia vs Quebec | 16.3 | (-19.7, 52.4) | 0.3738 | 11.0 | (-6.2, 28.3) | 0.2078 |
| Nova Scotia vs Ontario | -10.3 | (-47.2, 26.5) | 0.5815 | -5.5 | (-23.1, 12.2) | 0.5435 |
| Nova Scotia vs Manitoba | -30.2 | (-71.5, 11.2) | 0.1521 | -6.7 | (-26.5, 13.1) | 0.5061 |
| Nova Scotia vs Saskatchewan | 19.0 | (-27.9, 65.9) | 0.4260 | 12.1 | (-9.0, 33.3) | 0.2603 |
| Nova Scotia vs Alberta | -37.1 | (-77.7, 3.5) | 0.0731 | -19.6 | (-40.9, 1.6) | 0.0703 |
| Nova Scotia vs British Columbia | **49.0** | **(14.9, 83.1)** | **0.0049** | **23.6** | **(7.1, 40.1)** | **0.0053** |
| New Brunswick vs Quebec | 44.2 | (4.1, 84.4) | 0.0309 | **27.6** | **(7.4, 47.9)** | **0.0077** |
| New Brunswick vs Ontario | 17.5 | (-21.9, 56.9) | 0.3822 | 11.1 | (-8.9, 31.2) | 0.2752 |
| New Brunswick vs Manitoba | -2.3 | (-48.8, 44.2) | 0.9226 | 9.9 | (-13.5, 33.3) | 0.4067 |
| New Brunswick vs Saskatchewan | 46.9 | (-0.4, 94.2) | 0.0520 | **28.7** | **(5.6, 51.8)** | **0.0148** |
| New Brunswick vs Alberta | -9.2 | (-53.7, 35.3) | 0.6842 | -3.1 | (-27.8, 21.7) | 0.8085 |
| New Brunswick vs British Columbia | **76.9** | **(38.4, 115.4)** | **<.0001** | **40.2** | **(20.0, 60.3)** | **0.0001** |
| Quebec vs Ontario | -26.7 | (-54.2, 0.8) | 0.0571 | -16.5 | (-30.4, -2.6) | 0.0204 |
| Quebec vs Manitoba | **-46.5** | **(-85.2, -7.8)** | **0.0186** | -17.8 | (-36.2, 0.7) | 0.0590 |
| Quebec vs Saskatchewan | 2.7 | (-36.3, 41.6) | 0.8928 | 1.1 | (-16.9, 19.1) | 0.9061 |
| Quebec vs Alberta | **-53.4** | **(-87.2, -19.6)** | **0.0020** | **-30.7** | **(-49.8, -11.6)** | **0.0017** |
| Quebec vs British Columbia | **32.7** | **(7.2, 58.1)** | **0.0119** | 12.5 | (-0.3, 25.4) | 0.0559 |
| Ontario vs Manitoba | -19.8 | (-57.4, 17.8) | 0.3005 | -1.2 | (-19.8, 17.3) | 0.8952 |
| Ontario vs Saskatchewan | 29.4 | (-10.4, 69.1) | 0.1473 | 17.6 | (-1.2, 36.3) | 0.0660 |
| Ontario vs Alberta | -26.7 | (-59.7, 6.2) | 0.1117 | -14.2 | (-33.9, 5.6) | 0.1588 |
| Ontario vs British Columbia | **59.4** | **(33.5, 85.2)** | **<.0001** | **29.0** | **(14.9, 43.1)** | **<.0001** |
| Manitoba vs Saskatchewan | 49.2 | (2.9, 95.5) | 0.0372 | 18.8 | (-2.5, 40.2) | 0.0839 |
| Manitoba vs Alberta | -6.9 | (-48.6, 34.7) | 0.7446 | -12.9 | (-34.6, 8.7) | 0.2405 |
| Manitoba vs British Columbia | **79.2** | **(44.3, 114.1)** | **<.0001** | **30.3** | **(13.3, 47.3)** | **0.0005** |
| Saskatchewan vs Alberta | **-56.1** | **(-99.1, -13.1)** | **0.0107** | **-31.8** | **(-53.9, -9.7)** | **0.0050** |
| Saskatchewan vs British Columbia | 30.0 | (-7.9, 67.9) | 0.1204 | 11.4 | (-6.0, 28.9) | 0.1977 |
| Alberta vs British Columbia | **86.1** | **(54.5, 117.7)** | **<.0001** | **43.2** | **(24.5, 61.9)** | **<.0001** |
| **BMI category** | p=0.6788 | | | p=0.2394 | | |

Model statistics are for separate linear models using generalized least squares regression with F-Wald test of beverage volume and energy, with covariates sex, age, ethnicity, income, province, and BMI category, followed by Student t-test for pairwise comparisons. Pairwise contrasts not shown for covariates that did not have a significant overall effect. Values in bold font represent findings significant after post hoc adjustment using Benjamini-Hochberg procedure.

Abbreviations: 95% CI, 95% confidence interval; BMI, body-mass index; DK, don’t know; kcal, kilocalorie; NF, Newfoundland and Labrador; NS, not stated; PEI, Prince Edward Island

## Supplementary File Table 5. Pairwise comparisons of socio-demographic variables for daily per capita plain milk consumption in 2015 (N=20176)

|  | **Plain milk** | | | | | |
| --- | --- | --- | --- | --- | --- | --- |
|  | **Mean^a^ volume in ml** | | | **Mean^a^ energy in kcal** | | |
|  | **Estimate** | **(95% CI)** | **Adjusted  p-value** | **Estimate** | **(95% CI)** | **Adjusted  p-value** |
| **Model statistics** | F_(26,20176)_=73.54 | | | F_(26,20176)_=85.49 | | |
| **Sex** | **p<.0001** | | | **p<.0001** | | |
| Male vs female | **23.5** | **(13.0, 34.0)** | **<.0001** | **12.4** | **(7.0, 17.8)** | **<.0001** |
| **Age (years)** | **p<.0001** | | | **p<.0001** | | |
| 1-8 vs 9-18 | **79.8** | **(57.4, 102.2)** | **<.0001** | **50.9** | **(39.4, 62.3)** | **<.0001** |
| 1-8 vs 19-30 | **171.9** | **(139.9, 203.8)** | **<.0001** | **91.2** | **(73.7, 108.7)** | **<.0001** |
| 1-8 vs 31-50 | **191.9** | **(172.3, 211.5)** | **<.0001** | **104.6** | **(94.5, 114.7)** | **<.0001** |
| 1-8 vs 51+ | **186.6** | **(167.7, 205.5)** | **<.0001** | **103.1** | **(93.2, 113.0)** | **<.0001** |
| 9-18 vs 19-30 | **92.1** | **(63.3, 120.8)** | **<.0001** | **40.3** | **(24.5, 56.2)** | **<.0001** |
| 9-18 vs 31-50 | **112.1** | **(96.7, 127.5)** | **<.0001** | **53.7** | **(46.4, 61.0)** | **<.0001** |
| 9-18 vs 51+ | **106.8** | **(90.9, 122.6)** | **<.0001** | **52.2** | **(44.5, 60.0)** | **<.0001** |
| 19-30 vs 31-50 | 20.0 | (-6.7, 46.8) | 0.1417 | 13.4 | (-1.7, 28.5) | 0.0825 |
| 19-30 vs 51+ | 14.7 | (-13.0, 42.4) | 0.2964 | 11.9 | (-3.8, 27.6) | 0.1367 |
| 31-50 vs 51+ | -5.3 | (-17.1, 6.5) | 0.3769 | -1.5 | (-7.2, 4.2) | 0.6129 |
| **Ethnicity** | **p<.0001** | | | **p<.0001** | | |
| White only vs Chinese only | **35.8** | **(17.6, 54.1)** | **0.0001** | **14.4** | **(5.1, 23.7)** | **0.0025** |
| White only vs South Asian only | -43.8 | (-82.6, -5.0) | 0.0268 | **-29.1** | **(-51.1, -7.2)** | **0.0095** |
| White only vs Black only | **54.3** | **(25.2, 83.4)** | **0.0003** | **23.3** | **(7.7, 38.9)** | **0.0035** |
| White only vs Indigenous inclusive | 22.1 | (-4.3, 48.6) | 0.1000 | 7.8 | (-6.0, 21.5) | 0.2670 |
| White only vs Mixed/other/NS/missing | **44.4** | **(31.9, 56.9)** | **<.0001** | **18.2** | **(11.6, 24.8)** | **<.0001** |
| Chinese only vs South Asian only | **-79.6** | **(-120.6, -38.6)** | **0.0002** | **-43.5** | **(-66.3, -20.7)** | **0.0002** |
| Chinese only vs Black only | 18.5 | (-14.8, 51.7) | 0.2755 | 8.9 | (-8.6, 26.4) | 0.3178 |
| Chinese only vs Indigenous inclusive | -13.7 | (-44.4, 17.1) | 0.3836 | -6.6 | (-22.2, 9.0) | 0.4060 |
| Chinese only vs Mixed/other/NS/missing | 8.6 | (-11.4, 28.6) | 0.3979 | 3.8 | (-6.4, 14.1) | 0.4629 |
| South Asian only vs Black only | **98.1** | **(58.6, 137.6)** | **<.0001** | **52.4** | **(29.1, 75.7)** | **<.0001** |
| South Asian only vs Indigenous inclusive | **66.0** | **(17.0, 114.9)** | **0.0084** | **36.9** | **(9.9, 63.9)** | **0.0075** |
| South Asian only vs Mixed/other/NS/missing | **88.2** | **(49.7, 126.8)** | **<.0001** | **47.3** | **(25.6, 69.1)** | **<.0001** |
| Black only vs Indigenous inclusive | -32.1 | (-70.5, 6.2) | 0.1003 | -15.5 | (-35.7, 4.6) | 0.1308 |
| Black only vs Mixed/other/NS/missing | -9.9 | (-40.2, 20.5) | 0.5238 | -5.1 | (-21.4, 11.3) | 0.5425 |
| Indigenous inclusive vs Mixed/other/NS/missing | 22.3 | (-4.7, 49.3) | 0.1055 | 10.4 | (-3.6, 24.5) | 0.1454 |
| **Income (1=lowest income; 4=highest income)** | p=0.5967 | | | p=0.3396 | | |
| **Province** | **p=0.0002** | | | **p<.0001** | | |
| NL vs PEI | **-39.5** | **(-63.4, -15.7)** | **0.0012** | **-17.3** | **(-29.0, -5.6)** | **0.0039** |
| NL vs Nova Scotia | **-27.9** | **(-50.3, -5.6)** | **0.0142** | -11.5 | (-22.0, -1.1) | 0.0297 |
| NL vs New Brunswick | **-32.1** | **(-56.4, -7.7)** | **0.0099** | **-14.0** | **(-25.4, -2.6)** | **0.0166** |
| NL vs Quebec | **-37.4** | **(-58.6, -16.2)** | **0.0006** | **-22.0** | **(-33.1, -11.0)** | **0.0001** |
| NL vs Ontario | -18.0 | (-35.9, -0.1) | 0.0490 | -7.2 | (-15.9, 1.6) | 0.1082 |
| NL vs Manitoba | **-34.5** | **(-56.0, -13.0)** | **0.0017** | **-15.2** | **(-25.6, -4.9)** | **0.0041** |
| NL vs Saskatchewan | **-45.3** | **(-69.5, -21.0)** | **0.0003** | **-20.7** | **(-32.5, -9.0)** | **0.0006** |
| NL vs Alberta | -22.1 | (-41.5, -2.6) | 0.0264 | -10.0 | (-19.5, -0.4) | 0.0402 |
| NL vs British Columbia | -10.2 | (-29.4, 9.0) | 0.2978 | -4.0 | (-13.3, 5.3) | 0.4002 |
| PEI vs Nova Scotia | 11.6 | (-11.4, 34.5) | 0.3220 | 5.8 | (-5.0, 16.5) | 0.2930 |
| PEI vs New Brunswick | 7.4 | (-18.9, 33.8) | 0.5791 | 3.3 | (-9.0, 15.7) | 0.5955 |
| PEI vs Quebec | 2.1 | (-20.6, 24.9) | 0.8552 | -4.7 | (-16.3, 6.9) | 0.4258 |
| PEI vs Ontario | 21.5 | (0.3, 42.7) | 0.0465 | 10.2 | (-0.2, 20.5) | 0.0550 |
| PEI vs Manitoba | 5.0 | (-18.5, 28.5) | 0.6759 | 2.1 | (-9.3, 13.5) | 0.7174 |
| PEI vs Saskatchewan | -5.8 | (-34.3, 22.8) | 0.6924 | -3.4 | (-17.4, 10.5) | 0.6300 |
| PEI vs Alberta | 17.5 | (-4.9, 39.9) | 0.1265 | 7.3 | (-3.8, 18.5) | 0.1955 |
| PEI vs British Columbia | **29.3** | **(8.8, 49.9)** | **0.0052** | **13.3** | **(3.2, 23.4)** | **0.0098** |
| Nova Scotia vs New Brunswick | -4.1 | (-29.9, 21.7) | 0.7534 | -2.4 | (-14.2, 9.3) | 0.6851 |
| Nova Scotia vs Quebec | -9.5 | (-31.3, 12.4) | 0.3950 | -10.5 | (-21.3, 0.4) | 0.0583 |
| Nova Scotia vs Ontario | 9.9 | (-10.5, 30.3) | 0.3392 | 4.4 | (-5.0, 13.8) | 0.3594 |
| Nova Scotia vs Manitoba | -6.6 | (-28.7, 15.6) | 0.5602 | -3.7 | (-13.9, 6.6) | 0.4838 |
| Nova Scotia vs Saskatchewan | -17.3 | (-44.5, 9.9) | 0.2112 | -9.2 | (-22.0, 3.6) | 0.1582 |
| Nova Scotia vs Alberta | 5.9 | (-15.6, 27.3) | 0.5901 | 1.6 | (-8.4, 11.5) | 0.7560 |
| Nova Scotia vs British Columbia | 17.8 | (-2.1, 37.6) | 0.0796 | 7.6 | (-1.6, 16.8) | 0.1069 |
| New Brunswick vs Quebec | -5.3 | (-30.2, 19.5) | 0.6737 | -8.0 | (-20.3, 4.3) | 0.1995 |
| New Brunswick vs Ontario | 14.1 | (-8.8, 36.9) | 0.2271 | 6.8 | (-3.9, 17.5) | 0.2114 |
| New Brunswick vs Manitoba | -2.4 | (-27.1, 22.2) | 0.8461 | -1.2 | (-12.7, 10.2) | 0.8330 |
| New Brunswick vs Saskatchewan | -13.2 | (-43.0, 16.6) | 0.3847 | -6.8 | (-20.8, 7.3) | 0.3444 |
| New Brunswick vs Alberta | 10.0 | (-13.9, 33.9) | 0.4116 | 4.0 | (-7.3, 15.3) | 0.4859 |
| New Brunswick vs British Columbia | 21.9 | (-0.7, 44.5) | 0.0570 | 10.0 | (-0.5, 20.5) | 0.0630 |
| Quebec vs Ontario | 19.4 | (1.0, 37.8) | 0.0393 | **14.9** | **(5.0, 24.7)** | **0.0031** |
| Quebec vs Manitoba | 2.9 | (-19.9, 25.6) | 0.8029 | 6.8 | (-5.0, 18.7) | 0.2593 |
| Quebec vs Saskatchewan | -7.9 | (-34.2, 18.5) | 0.5578 | 1.3 | (-12.1, 14.7) | 0.8514 |
| Quebec vs Alberta | 15.3 | (-4.8, 35.5) | 0.1350 | 12.0 | (1.4, 22.7) | 0.0261 |
| Quebec vs British Columbia | **27.2** | **(8.4, 46.0)** | **0.0046** | **18.0** | **(8.0, 28.0)** | **0.0004** |
| Ontario vs Manitoba | -16.5 | (-35.1, 2.1) | 0.0813 | -8.0 | (-16.9, 0.9) | 0.0764 |
| Ontario vs Saskatchewan | -27.3 | (-50.4, -4.1) | 0.0209 | **-13.6** | **(-24.7, -2.4)** | **0.0172** |
| Ontario vs Alberta | -4.1 | (-20.8, 12.7) | 0.6350 | -2.8 | (-10.9, 5.2) | 0.4934 |
| Ontario vs British Columbia | 7.8 | (-6.3, 22.0) | 0.2781 | 3.2 | (-3.7, 10.1) | 0.3660 |
| Manitoba vs Saskatchewan | -10.8 | (-37.7, 16.2) | 0.4325 | -5.5 | (-18.4, 7.3) | 0.3989 |
| Manitoba vs Alberta | 12.5 | (-7.8, 32.7) | 0.2265 | 5.2 | (-4.5, 15.0) | 0.2918 |
| Manitoba vs British Columbia | **24.3** | **(4.7, 43.9)** | **0.0151** | **11.2** | **(2.0, 20.4)** | **0.0173** |
| Saskatchewan vs Alberta | 23.2 | (-2.0, 48.4) | 0.0707 | 10.8 | (-1.4, 22.9) | 0.0816 |
| Saskatchewan vs British Columbia | **35.1** | **(11.1, 59.1)** | **0.0042** | **16.7** | **(5.3, 28.2)** | **0.0043** |
| Alberta vs British Columbia | 11.9 | (-5.4, 29.1) | 0.1761 | 6.0 | (-2.4, 14.3) | 0.1595 |
| **BMI category** | p=0.4117 | | | p=0.5178 | | |

Model statistics are for separate linear models using generalized least squares regression with F-Wald test of beverage volume and energy, with covariates sex, age, ethnicity, income, province, and BMI category, followed by Student t-test for pairwise comparisons. Pairwise contrasts not shown for covariates that did not have a significant overall effect. Values in bold font represent findings significant after post hoc adjustment using Benjamini-Hochberg procedure.

Abbreviations: 95% CI, 95% confidence interval; BMI, body-mass index; DK, don’t know; kcal, kilocalorie; NF, Newfoundland and Labrador; NS, not stated; PEI, Prince Edward Island

## Supplementary File Table 6. Pairwise comparisons of socio-demographic variables for daily per capita alcoholic beverage consumption in 2015 (N=20176)

|  | **Alcoholic beverages** | | | | | |
| --- | --- | --- | --- | --- | --- | --- |
|  | **Mean^a^ volume in ml** | | | **Mean^a^ energy in kcal** | | |
|  | **Estimate** | **(95% CI)** | **Adjusted  p-value** | **Estimate** | **(95% CI)** | **Adjusted  p-value** |
| **Model statistics** | F_(26,20176)_=62.69 | | | F_(26,20176)_=51.42 | | |
| **Sex** | **p<.0001** | | | **p<.0001** | | |
| Male vs female | **113.4** | **(92.7, 134)** | **<.0001** | **50.5** | **(38.7, 62.2)** | **<.0001** |
| **Age (years)** | **p<.0001** | | | **p<.0001** | | |
| 1-8 vs 9-18 | -4.7 | (-13.1, 3.8) | 0.2812 | -2.9 | (-7.6, 1.9) | 0.2351 |
| 1-8 vs 19-30 | **-161.1** | **(-209.3, -112.9)** | **<.0001** | **-86.1** | **(-110.5, -61.8)** | **<.0001** |
| 1-8 vs 31-50 | **-149.8** | **(-173.9, -125.7)** | **<.0001** | **-84.4** | **(-98.3, -70.4)** | **<.0001** |
| 1-8 vs 51+ | **-124.0** | **(-141.0, -107.0)** | **<.0001** | **-83.1** | **(-94.2, -72.0)** | **<.0001** |
| 9-18 vs 19-30 | **-156.5** | **(-204.7, -108.2)** | **<.0001** | **-83.3** | **(-107.5, -59.1)** | **<.0001** |
| 9-18 vs 31-50 | **-145.2** | **(-170.0, -120.3)** | **<.0001** | **-81.5** | **(-95.8, -67.1)** | **<.0001** |
| 9-18 vs 51+ | **-119.4** | **(-136.5, -102.2)** | **<.0001** | **-80.2** | **(-91.6, -68.7)** | **<.0001** |
| 19-30 vs 31-50 | 11.3 | (-42.3, 64.9) | 0.6786 | 1.8 | (-26.0, 29.6) | 0.9000 |
| 19-30 vs 51+ | 37.1 | (-14.6, 88.8) | 0.1588 | 3.1 | (-23.3, 29.4) | 0.8185 |
| 31-50 vs 51+ | 25.8 | (-2.4, 54.0) | 0.0728 | 1.3 | (-15.6, 18.2) | 0.8802 |
| **Ethnicity** | **p<.0001** | | | **p<.0001** | | |
| White only vs Chinese only | **127.1** | **(106.7, 147.5)** | **<.0001** | **70.6** | **(59.8, 81.4)** | **<.0001** |
| White only vs South Asian only | **107.6** | **(86.9, 128.2)** | **<.0001** | **52.3** | **(36.3, 68.2)** | **<.0001** |
| White only vs Black only | **99.6** | **(74.3, 124.9)** | **<.0001** | **47.5** | **(30.9, 64.1)** | **<.0001** |
| White only vs Indigenous inclusive | **62.5** | **(30.8, 94.3)** | **0.0001** | **30.2** | **(10.6, 49.8)** | **0.0026** |
| White only vs Mixed/other/NS/missing | **72.1** | **(36.7, 107.5)** | **<.0001** | **34.1** | **(12.5, 55.6)** | **0.0020** |
| Chinese only vs South Asian only | -19.5 | (-39.5, 0.4) | 0.0547 | -18.3 | (-35.0, -1.7) | 0.0313 |
| Chinese only vs Black only | -27.5 | (-54.6, -0.4) | 0.0467 | **-23.1** | **(-40.5, -5.7)** | **0.0095** |
| Chinese only vs Indigenous inclusive | **-64.6** | **(-97.3, -31.8)** | **0.0001** | **-40.4** | **(-61.0, -19.8)** | **0.0001** |
| Chinese only vs Mixed/other/NS/missing | **-55.0** | **(-88.9, -21.1)** | **0.0015** | **-36.5** | **(-57.2, -15.8)** | **0.0006** |
| South Asian only vs Black only | -8.0 | (-32.9, 17.0) | 0.5307 | -4.7 | (-24.8, 15.3) | 0.6417 |
| South Asian only vs Indigenous inclusive | **-45.0** | **(-77.6, -12.5)** | **0.0068** | -22.0 | (-45.8, 1.7) | 0.0688 |
| South Asian only vs Mixed/other/NS/missing | -35.4 | (-70.4, -0.5) | 0.0470 | -18.2 | (-43.1, 6.7) | 0.1525 |
| Black only vs Indigenous inclusive | -37.0 | (-73.1, -1.0) | 0.0440 | -17.3 | (-41.0, 6.4) | 0.1524 |
| Black only vs Mixed/other/NS/missing | -27.5 | (-69.8, 14.8) | 0.2026 | -13.4 | (-39.1, 12.2) | 0.3037 |
| Indigenous inclusive vs Mixed/other/NS/missing | 9.6 | (-32.4, 51.5) | 0.6542 | 3.9 | (-22.6, 30.3) | 0.7740 |
| **Income (1=lowest income; 4=highest income)** | **p=0.0003** | | | **p<.0001** | | |
| Quartile 1 vs Quartile 2 | -30.0 | (-54.3, -5.6) | 0.0160 | **-24.9** | **(-39.3, -10.5)** | **0.0007** |
| Quartile 1 vs Quartile 3 | -30.4 | (-54.8, -6.1) | 0.0144 | **-25.1** | **(-38.6, -11.7)** | **0.0003** |
| Quartile 1 vs Quartile 4 | **-63.8** | **(-96.4, -31.1)** | **0.0001** | **-40.2** | **(-57.2, -23.2)** | **<.0001** |
| Quartile 1 vs Not reported | **-51.2** | **(-81.5, -20.9)** | **0.0010** | **-32.9** | **(-50.3, -15.5)** | **0.0002** |
| Quartile 2 vs Quartile 3 | -0.5 | (-27.1, 26.2) | 0.9731 | -0.2 | (-16.4, 16.0) | 0.9786 |
| Quartile 2 vs Quartile 4 | -33.8 | (-68.2, 0.6) | 0.0545 | -15.3 | (-34.2, 3.5) | 0.1109 |
| Quartile 2 vs Not reported | -21.2 | (-53.5, 11.2) | 0.1989 | -8.0 | (-27.5, 11.5) | 0.4197 |
| Quartile 3 vs Quartile 4 | -33.3 | (-68.0, 1.3) | 0.0594 | -15.1 | (-33.7, 3.5) | 0.1119 |
| Quartile 3 vs Not reported | -20.7 | (-53.4, 12.0) | 0.2139 | -7.8 | (-27.2, 11.6) | 0.4290 |
| Quartile 4 vs Not reported | 12.6 | (-26.5, 51.6) | 0.5265 | 7.3 | (-14.7, 29.2) | 0.5146 |
| **Province** | **p=0.0135** | | | **p=0.0029** | | |
| NL vs PEI | -6.4 | (-62.5, 49.8) | 0.8235 | 1.2 | (-25.3, 27.6) | 0.9314 |
| NL vs Nova Scotia | -34.3 | (-96.0, 27.4) | 0.2755 | -13.6 | (-40.6, 13.4) | 0.3223 |
| NL vs New Brunswick | -22.9 | (-77.2, 31.5) | 0.4088 | -14.8 | (-42.7, 13.1) | 0.2986 |
| NL vs Quebec | -50.6 | (-97.2, -3.9) | 0.0336 | **-33.5** | **(-58.7, -8.3)** | **0.0093** |
| NL vs Ontario | -23.6 | (-66.3, 19.1) | 0.2780 | -10.3 | (-31.6, 11.0) | 0.3420 |
| NL vs Manitoba | 3.9 | (-39.9, 47.7) | 0.8613 | -6.9 | (-31.1, 17.3) | 0.5777 |
| NL vs Saskatchewan | -8.2 | (-70.4, 54.0) | 0.7954 | -5.9 | (-36.1, 24.3) | 0.7016 |
| NL vs Alberta | -47.7 | (-99.7, 4.4) | 0.0725 | -31.0 | (-57.5, -4.5) | 0.0220 |
| NL vs British Columbia | -48.5 | (-93.5, -3.5) | 0.0348 | **-31.8** | **(-54.8, -8.9)** | **0.0066** |
| PEI vs Nova Scotia | -27.9 | (-87.5, 31.7) | 0.3582 | -14.8 | (-40.5, 10.9) | 0.2586 |
| PEI vs New Brunswick | -16.5 | (-69.1, 36.1) | 0.5381 | -16.0 | (-42.9, 11.0) | 0.2450 |
| PEI vs Quebec | -44.2 | (-88.5, 0.2) | 0.0508 | **-34.7** | **(-57.5, -11.8)** | **0.0030** |
| PEI vs Ontario | -17.2 | (-60.1, 25.7) | 0.4307 | -11.5 | (-31.5, 8.6) | 0.2607 |
| PEI vs Manitoba | 10.3 | (-33.3, 53.8) | 0.6432 | -8.0 | (-31.2, 15.1) | 0.4961 |
| PEI vs Saskatchewan | -1.8 | (-63.1, 59.4) | 0.9531 | -7.1 | (-36.2, 22.1) | 0.6348 |
| PEI vs Alberta | -41.3 | (-95.0, 12.4) | 0.1313 | -32.2 | (-59.7, -4.6) | 0.0222 |
| PEI vs British Columbia | -42.1 | (-86.7, 2.5) | 0.0642 | **-33.0** | **(-55.1, -10.9)** | **0.0034** |
| Nova Scotia vs New Brunswick | 11.4 | (-47.0, 69.8) | 0.7009 | -1.2 | (-27.6, 25.3) | 0.9310 |
| Nova Scotia vs Quebec | -16.3 | (-69.8, 37.3) | 0.5508 | -19.9 | (-45.3, 5.5) | 0.1246 |
| Nova Scotia vs Ontario | 10.7 | (-38.0, 59.4) | 0.6668 | 3.3 | (-18.2, 24.8) | 0.7628 |
| Nova Scotia vs Manitoba | 38.2 | (-15.5, 91.9) | 0.1628 | 6.8 | (-19.2, 32.7) | 0.6094 |
| Nova Scotia vs Saskatchewan | 26.1 | (-44.0, 96.1) | 0.4650 | 7.7 | (-24.0, 39.4) | 0.6322 |
| Nova Scotia vs Alberta | -13.4 | (-70.4, 43.7) | 0.6452 | -17.4 | (-45.8, 11.1) | 0.2304 |
| Nova Scotia vs British Columbia | -14.2 | (-66.7, 38.3) | 0.5957 | -18.2 | (-41.6, 5.2) | 0.1273 |
| New Brunswick vs Quebec | -27.7 | (-71.0, 15.6) | 0.2098 | -18.7 | (-44.0, 6.5) | 0.1458 |
| New Brunswick vs Ontario | -0.7 | (-40.4, 39.0) | 0.9710 | 4.5 | (-18.1, 27.0) | 0.6975 |
| New Brunswick vs Manitoba | 26.8 | (-15.7, 69.2) | 0.2161 | 7.9 | (-17.9, 33.8) | 0.5473 |
| New Brunswick vs Saskatchewan | 14.7 | (-45.0, 74.3) | 0.6296 | 8.9 | (-22.4, 40.1) | 0.5764 |
| New Brunswick vs Alberta | -24.8 | (-76.5, 26.9) | 0.3465 | -16.2 | (-45.9, 13.5) | 0.2840 |
| New Brunswick vs British Columbia | -25.6 | (-69.0, 17.8) | 0.2475 | -17.0 | (-41.9, 7.8) | 0.1790 |
| Quebec vs Ontario | 27.0 | (-3.9, 57.8) | 0.0864 | 23.2 | (3.7, 42.7) | 0.0197 |
| Quebec vs Manitoba | **54.5** | **(21.1, 87.8)** | **0.0014** | 26.6 | (4.4, 48.8) | 0.0188 |
| Quebec vs Saskatchewan | 42.4 | (-12.2, 96.9) | 0.1279 | 27.6 | (-1.7, 56.9) | 0.0643 |
| Quebec vs Alberta | 2.9 | (-42.1, 47.9) | 0.8996 | 2.5 | (-25.0, 30.0) | 0.8581 |
| Quebec vs British Columbia | 2.1 | (-32.8, 36.9) | 0.9061 | 1.7 | (-19.6, 23.0) | 0.8777 |
| Ontario vs Manitoba | 27.5 | (1.5, 53.5) | 0.0380 | 3.5 | (-13.8, 20.7) | 0.6934 |
| Ontario vs Saskatchewan | 15.4 | (-33.4, 64.2) | 0.5356 | 4.4 | (-19.7, 28.6) | 0.7191 |
| Ontario vs Alberta | -24.1 | (-61.6, 13.5) | 0.2085 | -20.7 | (-43.0, 1.6) | 0.0688 |
| Ontario vs British Columbia | -24.9 | (-50.8, 1.1) | 0.0607 | **-21.5** | **(-36.0, -7.0)** | **0.0037** |
| Manitoba vs Saskatchewan | -12.1 | (-61.6, 37.4) | 0.6312 | 1.0 | (-25.7, 27.6) | 0.9435 |
| Manitoba vs Alberta | -51.6 | (-93.9, -9.2) | 0.0171 | -24.1 | (-50.3, 2.0) | 0.0706 |
| Manitoba vs British Columbia | **-52.4** | **(-81.8, -23.0)** | **0.0005** | **-25.0** | **(-44.5, -5.5)** | **0.0121** |
| Saskatchewan vs Alberta | -39.5 | (-97.9, 19.0) | 0.1855 | -25.1 | (-54.8, 4.6) | 0.0973 |
| Saskatchewan vs British Columbia | -40.3 | (-91.6, 11.1) | 0.1244 | -25.9 | (-52.0, 0.1) | 0.0512 |
| Alberta vs British Columbia | -0.8 | (-42.1, 40.5) | 0.9697 | -0.8 | (-25.1, 23.4) | 0.9462 |
| **BMI category** | p=0.2204 | | | p=0.1439 | | |

Model statistics are for separate linear models using generalized least squares regression with F-Wald test of beverage volume and energy, with covariates sex, age, ethnicity, income, province, and BMI category, followed by Student t-test for pairwise comparisons. Pairwise contrasts not shown for covariates that did not have a significant overall effect. Values in bold font represent findings significant after post hoc adjustment using Benjamini-Hochberg procedure.

Abbreviations: 95% CI, 95% confidence interval; BMI, body-mass index; DK, don’t know; kcal, kilocalorie; NF, Newfoundland and Labrador; NS, not stated; PEI, Prince Edward Island

## Supplementary File Table 7. Pairwise comparisons of socio-demographic variables for daily per capita 100% juice consumption in 2015 (N=20176)

|  | **100% juice** | | | | | |
| --- | --- | --- | --- | --- | --- | --- |
|  | **Mean^a^ volume in ml** | | | **Mean^a^ energy in kcal** | | |
|  | **Estimate** | **(95% CI)** | **Adjusted  p-value** | **Estimate** | **(95% CI)** | **Adjusted  p-value** |
| **Model statistics** | F_(26,20176)_=43.04 | | | F_(26,20176)_=43.51 | | |
| **Sex** | **p<.0001** | | | **p<.0001** | | |
| Male vs female | **23.6** | **(15.2, 32.0)** | **<.0001** | **10.1** | **(6.2, 13.9)** | **<.0001** |
| **Age (years)** | **p<.0001** | | | **p<.0001** | | |
| 1-8 vs 9-18 | 2.8 | (-11.2, 16.9) | 0.6924 | 1.7 | (-5.0, 8.4) | 0.6217 |
| 1-8 vs 19-30 | **29.6** | **(6.4, 52.8)** | **0.0126** | **15.1** | **(4.1, 26.1)** | **0.0071** |
| 1-8 vs 31-50 | **54.4** | **(40.6, 68.2)** | **<.0001** | **27.1** | **(20.6, 33.5)** | **<.0001** |
| 1-8 vs 51+ | **60.2** | **(48.0, 72.4)** | **<.0001** | **30.0** | **(24.3, 35.8)** | **<.0001** |
| 9-18 vs 19-30 | **26.8** | **(5.3, 48.3)** | **0.0147** | **13.4** | **(3.3, 23.5)** | **0.0092** |
| 9-18 vs 31-50 | **51.5** | **(38.7, 64.4)** | **<.0001** | **25.4** | **(19.4, 31.3)** | **<.0001** |
| 9-18 vs 51+ | **57.4** | **(45.9, 68.9)** | **<.0001** | **28.3** | **(23.1, 33.6)** | **<.0001** |
| 19-30 vs 31-50 | 24.8 | (3.9, 45.7) | 0.0202 | 12.0 | (2.1, 21.8) | 0.0178 |
| 19-30 vs 51+ | **30.6** | **(9.1, 52.2)** | **0.0054** | **14.9** | **(4.8, 25.0)** | **0.0039** |
| 31-50 vs 51+ | 5.9 | (-3.9, 15.6) | 0.2405 | 3.0 | (-1.5, 7.5) | 0.1945 |
| **Ethnicity** | p=0.1130 | | | p=0.1870 | | |
| **Income (1=lowest income; 4=highest income)** | p=0.8946 | | | p=0.8863 | | |
| **Province** | **p<.0001** | | | **p<.0001** | | |
| NL vs PEI | -4.0 | (-28.1, 20.0) | 0.7415 | -1.4 | (-12.6, 9.8) | 0.8060 |
| NL vs Nova Scotia | 7.2 | (-10.2, 24.6) | 0.4146 | 4.2 | (-4.0, 12.3) | 0.3157 |
| NL vs New Brunswick | 3.5 | (-13.6, 20.6) | 0.6879 | 2.5 | (-5.4, 10.5) | 0.5317 |
| NL vs Quebec | **-43.2** | **(-62.1, -24.3)** | **<.0001** | **-17.9** | **(-26.9, -8.9)** | **0.0001** |
| NL vs Ontario | 8.3 | (-6.0, 22.7) | 0.2557 | 4.9 | (-2.0, 11.8) | 0.1599 |
| NL vs Manitoba | 14.0 | (-4.1, 32.2) | 0.1297 | 7.5 | (-0.9, 15.8) | 0.0793 |
| NL vs Saskatchewan | 13.4 | (-4.5, 31.2) | 0.1412 | 6.6 | (-2.0, 15.2) | 0.1302 |
| NL vs Alberta | **19.9** | **(3.8, 36.0)** | **0.0153** | **10.6** | **(3.1, 18.1)** | **0.0056** |
| NL vs British Columbia | 14.2 | (-1.9, 30.4) | 0.0839 | 8.2 | (0.5, 15.9) | 0.0365 |
| PEI vs Nova Scotia | 11.3 | (-11.7, 34.2) | 0.3353 | 5.6 | (-5.1, 16.3) | 0.3058 |
| PEI vs New Brunswick | 7.5 | (-15.6, 30.7) | 0.5228 | 4.0 | (-6.8, 14.7) | 0.4700 |
| PEI vs Quebec | **-39.2** | **(-63.3, -15.1)** | **0.0015** | **-16.5** | **(-27.6, -5.3)** | **0.0039** |
| PEI vs Ontario | 12.4 | (-8.4, 33.2) | 0.2439 | 6.3 | (-3.4, 16.1) | 0.2006 |
| PEI vs Manitoba | 18.1 | (-4.7, 40.8) | 0.1199 | 8.9 | (-1.6, 19.4) | 0.0978 |
| PEI vs Saskatchewan | 17.4 | (-5.4, 40.2) | 0.1341 | 8.0 | (-2.7, 18.7) | 0.1408 |
| PEI vs Alberta | 24.0 | (1.7, 46.2) | 0.0347 | 12.0 | (1.7, 22.3) | 0.0222 |
| PEI vs British Columbia | 18.3 | (-2.4, 38.9) | 0.0826 | 9.6019 | (-0.0006, 19.2043) | 0.0500 |
| Nova Scotia vs New Brunswick | -3.7 | (-20.2, 12.7) | 0.6575 | -1.6 | (-9.2, 5.9) | 0.6724 |
| Nova Scotia vs Quebec | **-50.4** | **(-67.1, -33.7)** | **<.0001** | **-22.0** | **(-29.9, -14.2)** | **<.0001** |
| Nova Scotia vs Ontario | 1.1 | (-12.1, 14.3) | 0.8701 | 0.8 | (-5.3, 6.9) | 0.8051 |
| Nova Scotia vs Manitoba | 6.8 | (-10.7, 24.3) | 0.4454 | 3.3 | (-4.6, 11.2) | 0.4117 |
| Nova Scotia vs Saskatchewan | 6.2 | (-10.3, 22.6) | 0.4618 | 2.4 | (-5.3, 10.2) | 0.5356 |
| Nova Scotia vs Alberta | 12.7 | (-1.3, 26.7) | 0.0752 | 6.5 | (0.1, 12.8) | 0.0465 |
| Nova Scotia vs British Columbia | 7.0 | (-8.3, 22.4) | 0.3699 | 4.0 | (-3.1, 11.2) | 0.2676 |
| New Brunswick vs Quebec | **-46.7** | **(-65.8, -27.6)** | **<.0001** | **-20.4** | **(-29.3, -11.5)** | **<.0001** |
| New Brunswick vs Ontario | 4.8 | (-8.4, 18.0) | 0.4744 | 2.4 | (-3.7, 8.5) | 0.4412 |
| New Brunswick vs Manitoba | 10.5 | (-7.1, 28.1) | 0.2411 | 4.9 | (-3.0, 12.8) | 0.2208 |
| New Brunswick vs Saskatchewan | 9.9 | (-7.0, 26.8) | 0.2512 | 4.1 | (-3.9, 12.0) | 0.3133 |
| New Brunswick vs Alberta | 16.4 | (1.3, 31.6) | 0.0336 | 8.1 | (1.3, 14.9) | 0.0203 |
| New Brunswick vs British Columbia | 10.7 | (-4.1, 25.5) | 0.1555 | 5.6 | (-1.2, 12.5) | 0.1050 |
| Quebec vs Ontario | **51.5** | **(35.8, 67.2)** | **<.0001** | **22.8** | **(15.5, 30.2)** | **<.0001** |
| Quebec vs Manitoba | **57.2** | **(38.7, 75.7)** | **<.0001** | **25.3** | **(16.9, 33.8)** | **<.0001** |
| Quebec vs Saskatchewan | **56.6** | **(37.9, 75.2)** | **<.0001** | **24.5** | **(15.7, 33.3)** | **<.0001** |
| Quebec vs Alberta | **63.1** | **(46.8, 79.5)** | **<.0001** | **28.5** | **(20.9, 36.2)** | **<.0001** |
| Quebec vs British Columbia | **57.4** | **(40.9, 74.0)** | **<.0001** | **26.1** | **(18.3, 33.8)** | **<.0001** |
| Ontario vs Manitoba | 5.7 | (-8.0, 19.4) | 0.4133 | 2.5 | (-3.6, 8.6) | 0.4152 |
| Ontario vs Saskatchewan | 5.1 | (-9.1, 19.2) | 0.4807 | 1.7 | (-5.0, 8.4) | 0.6219 |
| Ontario vs Alberta | 11.6 | (-0.5, 23.7) | 0.0597 | 5.7 | (0.2, 11.2) | 0.0437 |
| Ontario vs British Columbia | 5.9 | (-4.7, 16.6) | 0.2762 | 3.3 | (-1.8, 8.3) | 0.2020 |
| Manitoba vs Saskatchewan | -0.6 | (-17.4, 16.1) | 0.9407 | -0.8 | (-8.5, 6.8) | 0.8286 |
| Manitoba vs Alberta | 5.9 | (-9.6, 21.4) | 0.4543 | 3.2 | (-3.7, 10) | 0.3659 |
| Manitoba vs British Columbia | 0.2 | (-15.5, 15.9) | 0.9792 | 0.7 | (-6.3, 7.8) | 0.8394 |
| Saskatchewan vs Alberta | 6.5 | (-9.1, 22.2) | 0.4134 | 4.0 | (-3.3, 11.3) | 0.2790 |
| Saskatchewan vs British Columbia | 0.8 | (-15.0, 16.7) | 0.9168 | 1.6 | (-5.9, 9.1) | 0.6796 |
| Alberta vs British Columbia | -5.7 | (-19.1, 7.8) | 0.4061 | -2.4 | (-8.6, 3.8) | 0.4402 |
| **BMI category** | **p=0.0142** | | | **p=0.0268** | | |
| Underweight/normal vs Overweight | **17.0** | **(5.4, 28.7)** | **0.0042** | 6.6 | (1.1, 12.1) | 0.0179 |
| Underweight/normal vs Obese | **18.4** | **(5.4, 31.4)** | **0.0057** | **8.2** | **(2.4, 14.0)** | **0.0058** |
| Underweight/normal vs DK/Refusal/NS | 10.6 | (-0.8, 22.0) | 0.0683 | 4.0 | (-1.2, 9.2) | 0.1346 |
| Overweight vs Obese | 1.3 | (-11.0, 13.6) | 0.8321 | 1.6 | (-4.1, 7.3) | 0.5904 |
| Overweight vs DK/Refusal/NS | -6.4 | (-17.6, 4.8) | 0.2600 | -2.6 | (-7.9, 2.7) | 0.3298 |
| Obese vs DK/Refusal/NS | -7.8 | (-19.1, 3.6) | 0.1817 | -4.2 | (-9.3, 1.0) | 0.1123 |

Model statistics are for separate linear models using generalized least squares regression with F-Wald test of beverage volume and energy, with covariates sex, age, ethnicity, income, province, and BMI category, followed by Student t-test for pairwise comparisons. Pairwise contrasts not shown for covariates that did not have a significant overall effect. Values in bold font represent findings significant after post hoc adjustment using Benjamini-Hochberg procedure.

Abbreviations: 95% CI, 95% confidence interval; BMI, body-mass index; DK, don’t know; kcal, kilocalorie; NF, Newfoundland and Labrador; NS, not stated; PEI, Prince Edward Island

## Supplementary File Table 8. Pairwise comparisons of socio-demographic variables for daily per capita diet or light beverage consumption in 2015 (N=20176)

|  | **Diet or light beverages** | | | | | |
| --- | --- | --- | --- | --- | --- | --- |
|  | **Mean^a^ volume in ml** | | | **Mean^a^ energy in kcal** | | |
|  | **Estimate** | **(95% CI)** | **Adjusted  p-value** | **Estimate** | **(95% CI)** | **Adjusted  p-value** |
| **Model statistics** | F_(1,20176)_=27.22 | | | F_(1,20176)_=16.30 | | |
| **Sex** | p=0.5069 | | | p=0.3181 | | |
| **Age (years)** | **p<.0001** | | | **p<.0001** | | |
| 1-8 vs 9-18 | **-12.2** | **(-18.1, -6.3)** | **<.0001** | -0.44 | (-0.89, 0.02) | 0.0597 |
| 1-8 vs 19-30 | **-12.9** | **(-21.9, -3.9)** | **0.0053** | -0.3 | (-0.8, 0.2) | 0.3176 |
| 1-8 vs 31-50 | **-41.7** | **(-55.2, -28.2)** | **<.0001** | -0.5 | (-1.3, 0.3) | 0.1930 |
| 1-8 vs 51+ | **-38.5** | **(-47.3, -29.7)** | **<.0001** | **-1.4** | **(-2.2, -0.7)** | **0.0003** |
| 9-18 vs 19-30 | -0.7 | (-9.3, 7.9) | 0.8721 | 0.2 | (-0.3, 0.7) | 0.4656 |
| 9-18 vs 31-50 | **-29.5** | **(-43.6, -15.3)** | **<.0001** | -0.1 | (-0.8, 0.6) | 0.8086 |
| 9-18 vs 51+ | **-26.3** | **(-35.7, -17.0)** | **<.0001** | -1.01 | (-2.0, -0.02) | 0.0464 |
| 19-30 vs 31-50 | **-28.8** | **(-44.9, -12.7)** | **0.0005** | -0.3 | (-0.9, 0.4) | 0.4464 |
| 19-30 vs 51+ | **-25.6** | **(-37.2, -14.0)** | **<.0001** | -1.2 | (-2.2, -0.1) | 0.0280 |
| 31-50 vs 51+ | 3.2 | (-14.4, 20.8) | 0.7240 | -0.9 | (-2.3, 0.5) | 0.1969 |
| **Ethnicity** | **p<.0001** | | | **p=0.0015** | | |
| White only vs Chinese only | **29.0** | **(20.8, 37.2)** | **<.0001** | **0.9** | **(0.5, 1.4)** | **0.0001** |
| White only vs South Asian only | 13.1 | (-1.3, 27.6) | 0.0748 | 0.5 | (-0.1, 1.1) | 0.0983 |
| White only vs Black only | **32.1** | **(13.3, 51.0)** | **0.0009** | 0.7 | (-0.3, 1.7) | 0.1859 |
| White only vs Indigenous inclusive | -10.9 | (-38.7, 16.8) | 0.4395 | -0.8 | (-2.4, 0.9) | 0.3585 |
| White only vs Mixed/other/NS/missing | **18.3** | **(5.7, 30.9)** | **0.0044** | **0.6** | **(0.3, 1.0)** | **0.0008** |
| Chinese only vs South Asian only | -15.9 | (-29.4, -2.4) | 0.0212 | -0.4 | (-1.0, 0.2) | 0.1791 |
| Chinese only vs Black only | 3.1 | (-15.4, 21.6) | 0.7418 | -0.3 | (-1.2, 0.7) | 0.5807 |
| Chinese only vs Indigenous inclusive | **-39.9** | **(-66.6, -13.3)** | **0.0034** | -1.7 | (-3.3, -0.1) | 0.0420 |
| Chinese only vs Mixed/other/NS/missing | -10.7 | (-21.3, -0.1) | 0.0486 | -0.3 | (-0.7, 0.1) | 0.1854 |
| South Asian only vs Black only | 19.0 | (-2.4, 40.4) | 0.0824 | 0.1 | (-0.9, 1.1) | 0.7786 |
| South Asian only vs Indigenous inclusive | -24.1 | (-53.5, 5.4) | 0.1088 | -1.3 | (-3.0, 0.4) | 0.1320 |
| South Asian only vs Mixed/other/NS/missing | 5.2 | (-10.2, 20.5) | 0.5079 | 0.1 | (-0.5, 0.7) | 0.7070 |
| Black only vs Indigenous inclusive | **-43.0** | **(-75.9, -10.2)** | **0.0104** | -1.4 | (-3.3, 0.4) | 0.1284 |
| Black only vs Mixed/other/NS/missing | -13.8 | (-33.5, 5.9) | 0.1696 | -0.03 | (-0.99, 0.93) | 0.9504 |
| Indigenous inclusive vs Mixed/other/NS/missing | 29.2 | (0.8, 57.7) | 0.0438 | 1.4 | (-0.3, 3.1) | 0.0996 |
| **Income (1=lowest income; 4=highest income)** | **p=0.0288** | | | p=0.2099 | | |
| Quartile 1 vs Quartile 2 | 4.3 | (-7.1, 15.7) | 0.4589 | - | - | - |
| Quartile 1 vs Quartile 3 | -10.1 | (-24.8, 4.5) | 0.1752 | - | - | - |
| Quartile 1 vs Quartile 4 | -25.8 | (-47.1, -4.5) | 0.0177 | - | - | - |
| Quartile 1 vs Not reported | 0.7 | (-13.8, 15.2) | 0.9237 | - | - | - |
| Quartile 2 vs Quartile 3 | -14.4 | (-28.3, -0.6) | 0.0414 | - | - | - |
| Quartile 2 vs Quartile 4 | **-30.1** | **(-50.8, -9.4)** | **0.0045** | - | - | - |
| Quartile 2 vs Not reported | -3.6 | (-17.3, 10.1) | 0.6078 | - | - | - |
| Quartile 3 vs Quartile 4 | -15.7 | (-38.7, 7.3) | 0.1814 | - | - | - |
| Quartile 3 vs Not reported | 10.8 | (-7.0, 28.6) | 0.2320 | - | - | - |
| Quartile 4 vs Not reported | 26.5 | (2.8, 50.3) | 0.0287 | - | - | - |
| **Province** | **p<.0001** | | | **p<.0001** | | |
| NL vs PEI | 20.7 | (-11.8, 53.3) | 0.2113 | 0.3 | (-0.7, 1.4) | 0.5412 |
| NL vs Nova Scotia | 26.4 | (-1.5, 54.3) | 0.0634 | 0.2 | (-0.9, 1.3) | 0.7431 |
| NL vs New Brunswick | 23.2 | (-6.7, 53.1) | 0.1278 | -0.4 | (-1.7, 0.9) | 0.5273 |
| NL vs Quebec | 20.4 | (-10.5, 51.2) | 0.1952 | 0.4 | (-0.5, 1.4) | 0.3834 |
| NL vs Ontario | 28.3 | (0.8, 55.8) | 0.0441 | -0.4 | (-2.3, 1.6) | 0.7106 |
| NL vs Manitoba | 4.0 | (-35.4, 43.4) | 0.8409 | -1.0 | (-2.9, 0.9) | 0.2988 |
| NL vs Saskatchewan | 34.6 | (5.9, 63.2) | 0.0180 | 0.8 | (-0.1, 1.7) | 0.0646 |
| NL vs Alberta | 15.7 | (-14.5, 46.0) | 0.3082 | 0.2 | (-1.1, 1.4) | 0.8039 |
| NL vs British Columbia | **48.4** | **(22.9, 73.9)** | **0.0002** | 1.0 | (0.1, 2.0) | 0.0286 |
| PEI vs Nova Scotia | 5.7 | (-19.1, 30.5) | 0.6524 | -0.1 | (-1.1, 0.8) | 0.7711 |
| PEI vs New Brunswick | 2.5 | (-24.4, 29.3) | 0.8570 | -0.7 | (-2.0, 0.5) | 0.2388 |
| PEI vs Quebec | -0.4 | (-25.4, 24.6) | 0.9766 | 0.1 | (-0.5, 0.8) | 0.7412 |
| PEI vs Ontario | 7.5 | (-16.4, 31.5) | 0.5364 | -0.7 | (-2.1, 0.7) | 0.3413 |
| PEI vs Manitoba | -16.7 | (-51.4, 18.0) | 0.3444 | -1.3 | (-2.9, 0.3) | 0.1042 |
| PEI vs Saskatchewan | 13.8 | (-14.5, 42.1) | 0.3377 | 0.5 | (-0.4, 1.5) | 0.2788 |
| PEI vs Alberta | -5.0 | (-31.6, 21.6) | 0.7102 | -0.2 | (-1.2, 0.9) | 0.7505 |
| PEI vs British Columbia | **27.6** | **(5.7, 49.6)** | **0.0138** | 0.7 | (0.1, 1.3) | 0.0154 |
| Nova Scotia vs New Brunswick | -3.2 | (-25.3, 18.8) | 0.7741 | -0.6 | (-1.9, 0.7) | 0.3794 |
| Nova Scotia vs Quebec | -6.1 | (-26.1, 13.9) | 0.5517 | 0.3 | (-0.6, 1.1) | 0.5730 |
| Nova Scotia vs Ontario | 1.9 | (-14.8, 18.5) | 0.8271 | -0.5 | (-2.3, 1.2) | 0.5293 |
| Nova Scotia vs Manitoba | -22.4 | (-53.3, 8.5) | 0.1554 | -1.2 | (-2.9, 0.5) | 0.1724 |
| Nova Scotia vs Saskatchewan | 8.1 | (-13.9, 30.2) | 0.4692 | 0.7 | (-0.3, 1.7) | 0.1911 |
| Nova Scotia vs Alberta | -10.7 | (-33.1, 11.7) | 0.3472 | -0.02 | (-1.22, 1.17) | 0.9679 |
| Nova Scotia vs British Columbia | **22.0** | **(6.5, 37.4)** | **0.0054** | 0.86 | (0.04, 1.69) | 0.0408 |
| New Brunswick vs Quebec | -2.8 | (-26.2, 20.6) | 0.8118 | 0.8 | (-0.4, 2.1) | 0.1665 |
| New Brunswick vs Ontario | 5.1 | (-14.9, 25.0) | 0.6173 | 0.05 | (-1.87, 1.97) | 0.9603 |
| New Brunswick vs Manitoba | -19.2 | (-52.7, 14.4) | 0.2618 | -0.6 | (-2.5, 1.4) | 0.5578 |
| New Brunswick vs Saskatchewan | 11.4 | (-13.2, 35.9) | 0.3637 | 1.3 | (0.1, 2.5) | 0.0404 |
| New Brunswick vs Alberta | -7.5 | (-32.3, 17.3) | 0.5523 | 0.6 | (-0.9, 2.0) | 0.4356 |
| New Brunswick vs British Columbia | **25.2** | **(6.5, 43.9)** | **0.0085** | 1.5 | (0.3, 2.6) | 0.0121 |
| Quebec vs Ontario | 7.9 | (-9.2, 25.0) | 0.3639 | -0.8 | (-2.2, 0.6) | 0.2462 |
| Quebec vs Manitoba | -16.3 | (-47.6, 15.0) | 0.3055 | -1.42 | (-2.90, 0.05) | 0.0578 |
| Quebec vs Saskatchewan | 14.2 | (-10.1, 38.5) | 0.2515 | 0.4 | (-0.5, 1.3) | 0.3523 |
| Quebec vs Alberta | -4.7 | (-27.3, 18.0) | 0.6859 | -0.3 | (-1.2, 0.7) | 0.5580 |
| Quebec vs British Columbia | **28.0** | **(11.7, 44.4)** | **0.0008** | 0.6 | (0.2, 1.0) | 0.0066 |
| Ontario vs Manitoba | -24.3 | (-54.2, 5.7) | 0.1122 | -0.6 | (-2.6, 1.3) | 0.5262 |
| Ontario vs Saskatchewan | 6.3 | (-15.1, 27.6) | 0.5636 | 1.2 | (-0.7, 3.1) | 0.2075 |
| Ontario vs Alberta | -12.6 | (-32.5, 7.3) | 0.2145 | 0.5 | (-1.3, 2.3) | 0.5701 |
| Ontario vs British Columbia | **20.1** | **(8.5, 31.7)** | **0.0007** | 1.4 | (0.1, 2.7) | 0.0335 |
| Manitoba vs Saskatchewan | 30.5 | (-4.8, 65.9) | 0.0906 | 1.83 | (0.02, 3.64) | 0.0474 |
| Manitoba vs Alberta | 11.7 | (-22.7, 46.0) | 0.5045 | 1.1 | (-0.5, 2.8) | 0.1801 |
| Manitoba vs British Columbia | **44.4** | **(14.8, 74.0)** | **0.0034** | 2.0 | (0.5, 3.6) | 0.0102 |
| Saskatchewan vs Alberta | -18.9 | (-43.0, 5.3) | 0.1258 | -0.7 | (-1.8, 0.4) | 0.2241 |
| Saskatchewan vs British Columbia | 13.8 | (-5.3, 32.9) | 0.1555 | 0.2 | (-0.6, 1.0) | 0.6228 |
| Alberta vs British Columbia | **32.7** | **(14.3, 51.1)** | **0.0005** | 0.9 | (-0.1, 1.9) | 0.0762 |
| **BMI category** | **p<.0001** | | | **p=0.0009** | | |
| Underweight/normal vs Overweight | **-22.4** | **(-37.1, -7.8)** | **0.0027** | -0.6 | (-1.0, -0.2) | 0.0062 |
| Underweight/normal vs Obese | **-54.2** | **(-75.0, -33.5)** | **<.0001** | -2.9 | (-5.7, -0.1) | 0.0434 |
| Underweight/normal vs DK/Refusal/NS | **-15.8** | **(-24.8, -6.9)** | **0.0006** | -0.3 | (-0.8, 0.2) | 0.2362 |
| Overweight vs Obese | **-31.8** | **(-57.2, -6.4)** | **0.0142** | -2.3 | (-5.1, 0.5) | 0.1079 |
| Overweight vs DK/Refusal/NS | 6.6 | (-10.5, 23.7) | 0.4469 | 0.3 | (-0.3, 0.9) | 0.3514 |
| Obese vs DK/Refusal/NS | **38.4** | **(16.0, 60.8)** | **0.0008** | 2.6 | (-0.5, 5.7) | 0.0952 |

Model statistics are for separate linear models using generalized least squares regression with F-Wald test of beverage volume and energy, with covariates sex, age, ethnicity, income, province, and BMI category, followed by Student t-test for pairwise comparisons. Pairwise contrasts not shown for covariates that did not have a significant overall effect. Values in bold font represent findings significant after post hoc adjustment using Benjamini-Hochberg procedure.

Abbreviations: 95% CI, 95% confidence interval; BMI, body-mass index; DK, don’t know; kcal, kilocalorie; NF, Newfoundland and Labrador; NS, not stated; PEI, Prince Edward Island

# Appendix C: 2004 Beverage consumption

## Supplementary File Table 9. Daily per capita beverage volume intake (ml) from beverage categories in 2004, by socio-demographic variable (N=33463)

|  |  | **Plain water** | | **Other unsweetened** | | **SSBs** | | **Plain milk** | | **Alcoholic beverages** | | **100% juice** | | **Diet or light beverages** | |
| --- | --- | --- | --- | --- | --- | --- | --- | --- | --- | --- | --- | --- | --- | --- | --- |
|  |  | **Mean volume in ml**  **(95% CI)**  **Adjusted p-value** | | **Mean volume in ml**  **(95% CI)**  **Adjusted p-value** | | **Mean volume in ml**  **(95% CI)**  **Adjusted p-value** | | **Mean volume in ml**  **(95% CI)**  **Adjusted p-value** | | **Mean volume in ml**  **(95% CI)**  **Adjusted p-value** | | **Mean volume in ml**  **(95% CI)**  **Adjusted p-value** | | **Mean volume in ml**  **(95% CI)**  **Adjusted p-value** | |
| **Model statistics** | n | F_(26,34,463)_=57.08 | | F_(26,34,463)_=347.03 | | F_(26,34,463)_=153.20 | | F_(26,34,463)_=82.73 | | F_(26,34,463)_=96.56 | | F_(26,34,463)_=46.99 | | F_(26,34,463)_=37.12 | |
| **Sex** |  | p=0.0001 | | p=0.0012 | | p<.0001 | | p<.0001 | | p<.0001 | | p<.0001 | | p=0.0652 | |
| Male | 17330 | 753.6 | (725.7, 781.5) | 418.9 | (404.8, 433.0) | 332.0 | (318.2, 345.7) | 223.5 | (214.6, 232.5) | 207.5 | (191.6, 223.4) | 139.8 | (131.1, 148.5) | 49.2 | (42.7, 55.7) |
| Female | 17133 | 826.2 | (795.9, 856.5) | 401.0 | (386.9, 415.1) | 215.0 | (204.7, 225.4) | 191.9 | (184.9, 198.8) | 68.5 | (61.2, 75.9) | 109.7 | (103.7, 115.8) | 55.2 | (49.5, 60.9) |
| **Age (years)** |  | p<.0001 | | p<.0001 | | p<.0001 | | p<.0001 | | p<.0001 | | p<.0001 | | p<.0001 | |
| 1-8 | 3171 | 301.7 | (285.4, 318.1) | 12.8 | (9.2, 16.3) | 233.9 | (220.7, 247.1) | 367.3 | (352.9, 381.8) | 0.6 | (0.0, 1.6)* | 178.9 | (168.5, 189.4) | 7.1 | (5.1, 9.0) |
| 9-18 | 4668 | 617.6 | (590.6, 644.6) | 60.4 | (54.0, 66.8) | 492.4 | (473.8, 510.9) | 305.8 | (293.8, 317.8) | 23.2 | (16.0, 30.3) | 168.1 | (158.2, 178.0) | 31.0 | (26.7, 35.3) |
| 19-30 | 5538 | 1,012.8 | (965.3, 1060.2) | 301.1 | (279.7, 322.6) | 420.5 | (391.4, 449.6) | 195.5 | (178.2, 212.9) | 223.1 | (192.6, 253.6) | 157.9 | (139.5, 176.4) | 59.9 | (47.8, 72.1) |
| 31-50 | 10882 | 955.6 | (901.1, 1010.1) | 555.4 | (531.2, 579.7) | 254.0 | (233.9, 274.2) | 169.0 | (157.0, 181.0) | 192.5 | (172.3, 212.7) | 100.2 | (90.2, 110.3) | 71.8 | (61.0, 82.5) |
| 51+ | 10145 | 721.7 | (693.1, 750.2) | 598.4 | (580.4, 616.4) | 126.9 | (117.3, 136.5) | 161.1 | (152.9, 169.3) | 130.3 | (117.0, 143.6) | 96.5 | (89.2, 103.7) | 50.8 | (44.5, 57.1) |
| **Ethnicity** |  | p=0.0413 | | p<.0001 | | p<.0001 | | p<.0001 | | p<.0001 | | p=0.0011 | | p<.0001 | |
| White only | 28383 | 786.0 | (763.4, 808.6) | 435.2 | (423.7, 446.6) | 274.4 | (264.7, 284.1) | 211.3 | (205.0, 217.6) | 152.4 | (142.9, 161.9) | 124.1 | (118.4, 129.9) | 57.7 | (53.1, 62.4) |
| Chinese only | 1085 | 761.1 | (657.2, 864.9) | 283.2 | (238.3, 328.0) | 218.5 | (173.5, 263.4) | 196.1 | (166.1, 226.1) | 38.1 | (12.7, 63.4) | 96.7 | (73.8, 119.7) | 10.6 | (3.3, 18.0) |
| South Asian only | 1263 | 957.4 | (830.6, 1084.1) | 292.5 | (229.0, 355.9) | 236.4 | (170.5, 302.3) | 241.7 | (199.7, 283.8) | 55.5 | (13.1, 98.0) | 101.7 | (73.2, 130.2) | 13.9 | (5.1, 22.7) |
| Black only | 710 | 673.4 | (544.9, 801.9) | 194.7 | (144.4, 245.0) | 339.1 | (286.6, 391.6) | 156.5 | (122.0, 191.0) | 107.7 | (53.2, 162.2) | 217.3 | (172.6, 262.0) | 16.9 | (3.1, 30.7) |
| Indigenous inclusive | 628 | 839.0 | (717.2, 960.8) | 437.7 | (368.8, 506.6) | 397.1 | (347.7, 446.5) | 212.0 | (174.9, 249.1) | 143.2 | (74.4, 212.0) | 124.6 | (92.8, 156.4) | 47.2 | (28.6, 65.8) |
| Mixed/other/not stated/missing | 2335 | 779.7 | (693.2, 866.2) | 284.4 | (253.2, 315.6) | 259.9 | (231.5, 288.3) | 166.6 | (144.4, 188.9) | 67.9 | (42.9, 93.0) | 131.1 | (108.8, 153.4) | 37.0 | (11.7, 62.3) |
| **Income quartile** |  | p=0.0002 | | p=0.4051 | | p=0.0391 | | p=0.3275 | | p=0.0013 | | p=0.0038 | | p=0.0004 | |
| 1 (lowest income) | 6427 | 739.5 | (688.8, 790.2) | 394.3 | (372.2, 416.4) | 273.8 | (252.4, 295.2) | 202.1 | (189.7, 214.6) | 102.0 | (81.8, 122.2) | 111.2 | (100.2, 122.2) | 42.5 | (34.5, 50.5) |
| 2 | 6750 | 751.2 | (694.4, 808.0) | 412.4 | (383.6, 441.3) | 285.7 | (265.0, 306.3) | 216.9 | (201.1, 232.7) | 124.2 | (103.0, 145.5) | 120.6 | (109.7, 131.5) | 55.5 | (42.4, 68.6) |
| 3 | 6541 | 739.0 | (696.5, 781.6) | 421.2 | (399.0, 443.5) | 290.0 | (270.3, 309.8) | 218.6 | (204.9, 232.2) | 139.2 | (120.2, 158.1) | 125.6 | (115.5, 135.8) | 46.3 | (37.7, 55.0) |
| 4 (highest income) | 6550 | 881.9 | (835.1, 928.7) | 444.8 | (423.4, 466.2) | 239.9 | (218.1, 261.6) | 201.4 | (187.7, 215.0) | 202.1 | (177.8, 226.5) | 136.1 | (122.3, 149.9) | 78.9 | (66.4, 91.4) |
| Not reported | 8195 | 827.5 | (788.5, 866.6) | 383.5 | (361.9, 405.2) | 278.3 | (262.4, 294.2) | 201.2 | (189.3, 213.1) | 127.1 | (110.7, 143.5) | 129.5 | (118.0, 141.0) | 40.4 | (33.9, 47.0) |
| **Province** |  | p<.0001 | | p<.0001 | | p=0.1764 | | p<.0001 | | p<.0001 | | p<.0001 | | p=0.0005 | |
| NL | 571 | 540.3 | (480.9, 599.8) | 414.0 | (375.2, 452.8) | 304.4 | (278.2, 330.7) | 174.6 | (157.6, 191.5) | 119.5 | (85.1, 154.0) | 130.9 | (115.0, 146.9) | 96.8 | (74.6, 118.9) |
| PEI | 152 | 776.5 | (701.3, 851.6) | 399.3 | (358.9, 439.6) | 260.7 | (231.1, 290.3) | 295.7 | (255.2, 336.2) | 73.4 | (53.6, 93.2) | 110.6 | (93.9, 127.2) | 51.5 | (36.4, 66.6) |
| Nova Scotia | 1021 | 792.5 | (706.9, 878.1) | 470.7 | (422.0, 519.3) | 287.3 | (250.1, 324.6) | 250.4 | (225.8, 274.9) | 145.0 | (101.2, 188.7) | 101.2 | (85.7, 116.7) | 48.5 | (37.1, 59.9) |
| New Brunswick | 813 | 850.6 | (768.6, 932.5) | 447.9 | (403.9, 491.8) | 287.8 | (243.1, 332.4) | 237.5 | (215.6, 259.5) | 132.4 | (98.5, 166.3) | 107.1 | (89.4, 124.7) | 61.2 | (46.7, 75.6) |
| Quebec | 8192 | 753.6 | (712.8, 794.5) | 372.6 | (348.6, 396.5) | 268.6 | (247.0, 290.2) | 200.5 | (186.4, 214.7) | 163.1 | (140.9, 185.3) | 154.9 | (140.5, 169.2) | 51.5 | (41.2, 61.7) |
| Ontario | 13531 | 765.7 | (729.8, 801.6) | 402.2 | (386.4, 417.9) | 265.4 | (251.6, 279.1) | 201.4 | (192.8, 210.1) | 125.0 | (112.8, 137.3) | 124.9 | (117.1, 132.7) | 55.9 | (47.8, 64.1) |
| Manitoba | 1208 | 712.3 | (657.6, 766.9) | 435.1 | (406.0, 464.3) | 297.0 | (270.4, 323.5) | 246.6 | (219.5, 273.8) | 120.6 | (95.8, 145.4) | 100.7 | (89.8, 111.7) | 58.5 | (45.8, 71.2) |
| Saskatchewan | 1020 | 871.3 | (792.7, 949.9) | 471.0 | (427.3, 514.7) | 321.6 | (278.7, 364.5) | 237.6 | (212.4, 262.7) | 98.7 | (73.8, 123.5) | 103.1 | (86.4, 119.8) | 34.3 | (22.3, 46.4) |
| Alberta | 3400 | 773.7 | (718.0, 829.3) | 414.6 | (383.3, 445.9) | 314.9 | (282.9, 346.9) | 219.7 | (199.0, 240.3) | 125.8 | (93.4, 158.1) | 101.9 | (89.0, 114.8) | 50.1 | (36.9, 63.3) |
| British Columbia | 4497 | 962.6 | (894.3, 1030.8) | 457.0 | (424.9, 489.0) | 251.7 | (230.1, 273.3) | 200.2 | (184.7, 215.7) | 161.3 | (136.4, 186.2) | 107.1 | (95.8, 118.4) | 39.7 | (30.9, 48.5) |
| **BMI category** |  | p=0.3221 | | p=0.8116 | | p=0.0130 | | p=0.6369 | | p=0.5261 | | p=0.5079 | | p<.0001 | |
| Underweight/normal | 9319 | 750.3 | (712.6, 788.1) | 339.2 | (318.1, 360.3) | 319.8 | (303.6, 335.9) | 227.7 | (215.8, 239.6) | 116.6 | (99.6, 133.5) | 133.4 | (123.6, 143.1) | 25.8 | (21.3, 30.3) |
| Overweight | 6571 | 837.2 | (778.4, 896.0) | 452.8 | (429.3, 476.3) | 265.7 | (244.5, 286.9) | 202.1 | (189.5, 214.7) | 153.5 | (131.0, 176.0) | 120.4 | (109.6, 131.1) | 61.3 | (47.5, 75.1) |
| Obese | 4204 | 822.1 | (762.6, 881.6) | 483.0 | (454.6, 511.4) | 258.0 | (234.3, 281.6) | 195.0 | (178.0, 212.1) | 132.2 | (109.2, 155.2) | 103.8 | (89.7, 117.9) | 96.4 | (78.8, 113.9) |
| Don’t know/refusal/not stated | 14309 | 784.0 | (752.8, 815.2) | 415.0 | (399.3, 430.8) | 252.3 | (238.6, 266.0) | 201.2 | (192.2, 210.1) | 147.5 | (133.4, 161.7) | 127.6 | (118.5, 136.7) | 52.2 | (45.8, 58.6) |

All means are weighted ‘arithmetic means’ (also referred to as ‘observed means’), and not linear regression adjusted least squares means. Models statistics are for separate linear models using generalized least squares regression for each beverage category, with covariates sex, age, ethnicity, income, province, and BMI category; α = 0.05.

Abbreviations: 95% CI, 95% confidence interval; BMI, body-mass index; ml, millilitre; NF, Newfoundland and Labrador; PEI, Prince Edward Island; SSBs, sugar-sweetened beverages

*Estimates for these 95% confidence intervals contained values less than zero. For reporting, these values were replaced with ‘0’ as they were a result of the bootstrap resampling method and not an indication of negative consumption.

## Supplementary File Table 10. Daily per capita energy intake (kcal) from beverage categories in 2004, by socio-demographic variable (N=34,463)

|  |  | **Other unsweetened** | | **SSBs** | | **Plain milk** | | **Alcoholic beverages** | | **100% juice** | | **Diet or light beverages** | |
| --- | --- | --- | --- | --- | --- | --- | --- | --- | --- | --- | --- | --- | --- |
|  |  | **Mean energy in kcal**  **(95% CI)**  **Adjusted p-value** | | **Mean energy in kcal**  **(95% CI)**  **Adjusted p-value** | | **Mean energy in kcal**  **(95% CI)**  **Adjusted p-value** | | **Mean energy in kcal**  **(95% CI)**  **Adjusted p-value** | | **Mean energy in kcal**  **(95% CI)**  **Adjusted p-value** | | **Mean energy in kcal**  **(95% CI)**  **Adjusted p-value** | |
| **Model statistics** | n | F_(26,34,463)_=57.47 | | F_(26,34,463)_=153.55 | | F_(26,34,463)_=95.98 | | F_(26,34,463)_=86.60 | | F_(26,34,463)_=49.02 | | F_(26,34,463)_=5.86 | |
| **Sex** |  | p=0.2783 | | p<.0001 | | p<.0001 | | p<.0001 | | p<.0001 | | p=0.5814 | |
| Male | 17330 | 9.8 | (8.9, 10.7) | 148.8 | (142.7, 154.9) | 107.2 | (103.0, 111.4) | 102.1 | (94.6, 109.5) | 60.6 | (56.7, 64.4) | 3.1 | (2.3, 3.9) |
| Female | 17133 | 9.4 | (8.7, 10.1) | 97.8 | (93.5, 102.1) | 91.4 | (87.9, 94.9) | 43.2 | (38.1, 48.3) | 47.7 | (45.0, 50.4) | 2.7 | (2.1, 3.3) |
| **Age (years)** |  | p<.0001 | | p<.0001 | | p<.0001 | | p<.0001 | | p<.0001 | | p<.0001 | |
| 1-8 | 3171 | 0.1 | (0.1, 0.2) | 122.2 | (115.1, 129.3) | 189.9 | (182.0, 197.8) | 0.3 | (0.0, 0.9)* | 81.8 | (77.0, 86.6) | 0.5 | (0.1, 0.9) |
| 9-18 | 4668 | 1.6 | (1.3, 2.0) | 230.7 | (221.9, 239.6) | 146.4 | (140.5, 152.4) | 13.5 | (7.9, 19.2) | 74.9 | (70.5, 79.3) | 2.1 | (0.9, 3.2) |
| 19-30 | 5538 | 8.9 | (7.3, 10.5) | 185.3 | (171.6, 199.0) | 91.0 | (82.8, 99.1) | 113.8 | (98.2, 129.5) | 69.3 | (60.9, 77.7) | 5.5 | (3.7, 7.3) |
| 31-50 | 10882 | 13.2 | (12.0, 14.4) | 109.2 | (101.1, 117.3) | 79.7 | (74.0, 85.5) | 98.9 | (88.7, 109.1) | 42.3 | (37.9, 46.6) | 3.1 | (2.1, 4.0) |
| 51+ | 10145 | 12.8 | (11.7, 13.8) | 56.0 | (51.6, 60.4) | 75.1 | (71.3, 78.9) | 72.3 | (65.6, 79.0) | 40.5 | (37.4, 43.6) | 2.4 | (1.6, 3.2) |
| **Ethnicity** |  | p<.0001 | | p<.0001 | | p=0.0002 | | p<.0001 | | p=0.0011 | | p=0.1454 | |
| White only | 28383 | 10.4 | (9.8, 11.0) | 123.9 | (119.7, 128.1) | 100.0 | (97.0, 103.0) | 80.3 | (75.4, 85.2) | 53.6 | (51.0, 56.2) | 3.1 | (2.5, 3.7) |
| Chinese only | 1085 | 5.7 | (3.8, 7.7) | 95.2 | (74.4, 116.0) | 96.2 | (81.3, 111.2) | 17.8 | (7.4, 28.3) | 42.1 | (31.9, 52.2) | 2.7 | (0.0, 5.9)* |
| South Asian only | 1263 | 4.4 | (3.0, 5.8) | 97.4 | (74.2, 120.5) | 122.4 | (102.5, 142.4) | 27.5 | (9.7, 45.2) | 45.4 | (32.2, 58.7) | 1.8 | (0.2, 3.5) |
| Black only | 710 | 3.1 | (2.3, 3.9) | 172.9 | (143.8, 201.9) | 82.2 | (63.2, 101.3) | 57.0 | (26.3, 87.8) | 97.3 | (77.0, 117.6) | 1.2 | (0.0, 2.9)* |
| Indigenous inclusive | 628 | 8.1 | (6.7, 9.4) | 171.4 | (149.5, 193.2) | 105.8 | (85.8, 125.9) | 68.7 | (36.9, 100.5) | 57.0 | (42.2, 71.7) | 2.1 | (0.0, 4.4)* |
| Mixed/other/not stated/missing | 2335 | 7.3 | (4.7, 10.0) | 117.5 | (104.4, 130.5) | 84.2 | (72.6, 95.8) | 37.4 | (24.9, 49.8) | 57.5 | (47.6, 67.4) | 1.8 | (0.3, 3.3) |
| **Income quartile** |  | p=0.2488 | | p=0.0289 | | p=0.4732 | | p<.0001 | | p=0.0065 | | p=0.2298 | |
| 1 (lowest income) | 6427 | 9.2 | (7.9, 10.4) | 124.2 | (113.8, 134.6) | 101.3 | (95.2, 107.5) | 51.6 | (41.5, 61.8) | 48.4 | (43.8, 53.1) | 2.0 | (0.9, 3.1) |
| 2 | 6750 | 9.0 | (7.8, 10.1) | 128.2 | (119.0, 137.4) | 105.1 | (97.4, 112.8) | 62.5 | (52.5, 72.6) | 52.1 | (47.3, 56.9) | 2.3 | (1.3, 3.3) |
| 3 | 6541 | 9.0 | (8.1, 9.9) | 130.4 | (121.5, 139.3) | 103.6 | (97.1, 110.0) | 73.4 | (62.9, 83.9) | 54.5 | (50.0, 59.0) | 2.8 | (1.7, 4.0) |
| 4 (highest income) | 6550 | 12.0 | (10.3, 13.8) | 107.5 | (98.9, 116.0) | 92.0 | (85.8, 98.2) | 111.8 | (98.5, 125.1) | 57.7 | (52.0, 63.4) | 4.6 | (3.0, 6.1) |
| Not reported | 8195 | 9.1 | (8.0, 10.1) | 126.1 | (118.7, 133.6) | 95.7 | (89.9, 101.4) | 66.2 | (58.1, 74.4) | 57.2 | (51.7, 62.7) | 2.7 | (1.8, 3.7) |
| **Province** |  | p<.0001 | | p=0.3398 | | p<.0001 | | p<.0001 | | p<.0001 | | p=0.0046 | |
| NL | 571 | 6.9 | (5.9, 8.0) | 134.2 | (122.3, 146.1) | 83.7 | (75.1, 92.3) | 69.4 | (36.8, 101.9) | 57.0 | (50.1, 63.9) | 3.6 | (0.7, 6.4) |
| PEI | 152 | 7.0 | (6.0, 8.0) | 128.3 | (111.8, 144.9) | 143.2 | (122.5, 164.0) | 39.3 | (28.2, 50.4) | 48.0 | (40.6, 55.3) | 2.1 | (0.8, 3.3) |
| Nova Scotia | 1021 | 8.3 | (7.2, 9.4) | 131.7 | (114.6, 148.7) | 118.8 | (106.6, 131.0) | 70.6 | (51.6, 89.7) | 43.3 | (37.2, 49.4) | 3.8 | (0.1, 7.4) |
| New Brunswick | 813 | 8.5 | (7.0, 10.0) | 136.6 | (109.4, 163.7) | 113.1 | (102.6, 123.6) | 58.5 | (44.4, 72.7) | 47.4 | (39.3, 55.5) | 4.6 | (1.1, 8.2) |
| Quebec | 8192 | 11.7 | (10.0, 13.3) | 120.5 | (110.8, 130.1) | 100.2 | (93.2, 107.2) | 82.6 | (71.7, 93.6) | 64.6 | (58.6, 70.6) | 1.3 | (0.8, 1.9) |
| Ontario | 13531 | 8.4 | (7.7, 9.1) | 120.2 | (114.5, 126.0) | 95.7 | (91.6, 99.9) | 65.0 | (58.9, 71.2) | 55.3 | (51.6, 58.9) | 3.2 | (2.2, 4.1) |
| Manitoba | 1208 | 9.1 | (7.8, 10.4) | 133.2 | (121.6, 144.8) | 112.6 | (101.1, 124.0) | 63.4 | (50.4, 76.5) | 44.2 | (39.3, 49.0) | 3.0 | (0.8, 5.2) |
| Saskatchewan | 1020 | 9.1 | (8.1, 10.0) | 146.0 | (129.7, 162.3) | 110.2 | (98.8, 121.7) | 51.7 | (38.9, 64.5) | 46.0 | (38.1, 54.0) | 2.0 | (0.8, 3.2) |
| Alberta | 3400 | 10.0 | (8.0, 11.9) | 138.9 | (126.2, 151.6) | 103.0 | (92.9, 113.0) | 73.1 | (53.3, 93.0) | 45.0 | (39.1, 50.8) | 2.8 | (1.9, 3.7) |
| British Columbia | 4497 | 10.5 | (9.0, 12.0) | 113.5 | (103.8, 123.1) | 93.6 | (86.7, 100.4) | 89.9 | (77.7, 102.2) | 46.9 | (41.7, 52.1) | 4.6 | (2.6, 6.5) |
| **BMI category** |  | p=0.4888 | | p=0.0306 | | p=0.8416 | | p=0.8404 | | p=0.2915 | | p=0.3347 | |
| Underweight/normal | 9319 | 8.4 | (7.4, 9.4) | 145.5 | (138.5, 152.5) | 110.1 | (104.2, 115.9) | 62.4 | (53.9, 71.0) | 57.9 | (53.8, 62.1) | 2.1 | (1.3, 3.0) |
| Overweight | 6571 | 10.6 | (9.1, 12.1) | 118.4 | (109.6, 127.2) | 94.8 | (89.0, 100.6) | 82.7 | (71.2, 94.3) | 51.8 | (47.2, 56.5) | 2.8 | (1.9, 3.8) |
| Obese | 4204 | 10.2 | (9.1, 11.3) | 113.1 | (103.0, 123.2) | 90.9 | (82.8, 99.1) | 74.9 | (60.1, 89.7) | 43.8 | (38.4, 49.1) | 3.9 | (2.0, 5.8) |
| Don't know/refusal/not stated | 14309 | 9.8 | (8.9, 10.6) | 114.4 | (107.9, 121.0) | 97.0 | (92.7, 101.2) | 74.4 | (67.6, 81.2) | 55.8 | (51.6, 60.0) | 3.1 | (2.2, 3.9) |

No model was constructed for plain water as this beverage category contained no energy. All means are weighted ‘arithmetic means’ (also referred to as ‘observed means’), and not linear regression adjusted least squares means. Models statistics are for separate linear models using generalized least squares regression for each beverage category, with covariates sex, age, ethnicity, income, province, and BMI category; α = 0.05.

Abbreviations: 95% CI, 95% confidence interval; BMI, body-mass index; kcal, kilocalorie; NF, Newfoundland and Labrador; PEI, Prince Edward Island; SSBs, sugar-sweetened beverages

*Estimates for these 95% confidence intervals contained values less than zero. For reporting, these values were replaced with ‘0’ as they were a result of the bootstrap resampling method and not an indication of negative consumption.

# Appendix D: 2004 Additional analyses of national and age-sex consumption

## Supplementary File Table 11. Daily per capita volume intake (ml) from beverage categories and sub-categories in 2004, by all and age-sex group

|  | **ALL** | | **CHILDREN AND YOUTH 1-18 yrs** | | | | **ADULTS 19+ yrs** | | | |
| --- | --- | --- | --- | --- | --- | --- | --- | --- | --- | --- |
|  |  | | **Males** | | **Females** | | **Males** | | **Females** | |
|  | **Mean volume in ml**  **(95% CI)** | | **Mean volume in ml**  **(95% CI)** | | **Mean volume in ml**  **(95% CI)** | | **Mean volume in ml**  **(95% CI)** | | **Mean volume in ml**  **(95% CI)** | |
| n | N=34,363 | | n=4,009 | | n=3,843 | | n=13,321 | | n=13,291 | |
| **Plain water** | 789.7 | (769.2, 810.2) | 508.8 | (484.7, 532.9) | 470.0 | (445.7, 494.3) | 827.3 | (791.3, 863.2) | 929.2 | (890.9, 967.5) |
| Other water (tap, well) | 505.4 | (487.5, 523.2) | 347.8 | (325.5, 370.0) | 300.7 | (279.8, 321.5) | 545.7 | (512.1, 579.3) | 571.7 | (542.0, 601.4) |
| Bottled water | 284.3 | (266.6, 302.1) | 161.0 | (144.4, 177.6) | 169.3 | (152.1, 186.6) | 281.5 | (255.5, 307.5) | 357.6 | (320.0, 395.2) |
| **Other unsweetened** | 410.0 | (399.7, 420.3) | 38.5 | (32.9, 44.2) | 43.8 | (38.1, 49.5) | 533.4 | (515.0, 551.7) | 504.3 | (486.3, 522.3) |
| Unsweetened coffee | 284.5 | (275.3, 293.7) | 12.3 | (9.2, 15.4) | 15.5 | (12.3, 18.6) | 401.7 | (384.8, 418.7) | 326.9 | (311.8, 342.0) |
| Unsweetened tea | 123.9 | (118.1, 129.6) | 26.1 | (21.3, 30.8) | 27.9 | (23.1, 32.8) | 130.0 | (120.2, 139.8) | 174.9 | (164.5, 185.3) |
| Club soda and other^a^ | 1.6 | (1.1, 2.2) | 0.2 | (0.0, 0.4)* | 0.5 | (0, 0.9) | 1.6 | (0.8, 2.4) | 2.4 | (1.2, 3.7) |
| **SSBs** | 273.8 | (265.1, 282.6) | 448.3 | (428.2, 468.4) | 324.7 | (309.9, 339.5) | 296.9 | (279.9, 314.0) | 183.3 | (170.4, 196.2) |
| Regular carbonated soft drinks | 132.3 | (126.0, 138.7) | 178.6 | (165.0, 192.1) | 98.7 | (90.5, 106.9) | 176.8 | (163.5, 190.0) | 83.6 | (75.4, 91.8) |
| Regular fruit drinks | 78.7 | (74.7, 82.7) | 165.9 | (154.6, 177.3) | 148.8 | (138.7, 158.9) | 60.5 | (53.3, 67.7) | 50.4 | (45.1, 55.7) |
| Sugar-sweetened milk | 21.8 | (19.9, 23.8) | 56.2 | (49.5, 62.9) | 42.9 | (37.7, 48.1) | 15.4 | (12.0, 18.8) | 11.9 | (9.2, 14.6) |
| Tea pre-sweetened with sugar | 18.8 | (16.2, 21.5) | 20.9 | (16.1, 25.6) | 14.4 | (11.4, 17.4) | 18.2 | (14.5, 21.9) | 20.2 | (14.5, 25.8) |
| Regular sports drinks | 8.2 | (6.0, 10.3) | 14.5 | (10.1, 18.9) | 5.7 | (3.9, 7.4) | 12.2 | (7.1, 17.2) | 3.0 | (1.0, 5.0) |
| Hot chocolate pre-sweetened with sugar | 5.4 | (4.3, 6.5) | 6.4 | (4.5, 8.2) | 7.4 | (5.2, 9.6) | 5.8 | (3.5, 8.2) | 4.0 | (2.9, 5.2) |
| Smoothies | 3.7 | (1.7, 5.7) | 3.1 | (1.6, 4.7) | 3.9 | (2.3, 5.5) | 4.4 | (0.0, 9.4)* | 3.1 | (1.9, 4.3) |
| Coffee pre-sweetened with sugar | 3.1 | (2.2, 4.1) | 1.0 | (0.5, 1.5) | 1.4 | (0.6, 2.2) | 2.3 | (1.3, 3.2) | 5.2 | (3.0, 7.4) |
| Regular meal replacement beverages | 1.0 | (0.7, 1.3) | 1.2 | (0.5, 2.0) | 0.8 | (0.3, 1.3) | 0.7 | (0.4, 1.1) | 1.3 | (0.8, 1.9) |
| Other SSBs^b^ | 0.5 | (0.2, 0.7) | 0.23 | (0.02, 0.45) | 0.72 | (0.01, 1.42) | 0.5 | (0.0, 1.0)* | 0.4 | (0.1, 0.8) |
| Regular energy drinks | 0.1 | (0.0, 0.3)* | - | - | 0.02 | (0.00, 0.06)* | 0.2 | (0.0, 0.5)* | 0.1 | (0.0, 0.3)* |
| Flavoured drinkable yogurt | 0.06 | (0.01, 0.12) | 0.2 | (0.0, 0.5)* | - | - | 0.1 | (0.0, 0.2)* | 0.03 | (0.00, 0.08)* |
| **Plain milk** | 207.8 | (202.2, 213.4) | 369.1 | (355.3, 383) | 290.6 | (277.9, 303.4) | 179.7 | (168.5, 191.0) | 163.3 | (155.2, 171.4) |
| **Alcoholic beverages** | 138.4 | (129.7, 147.1) | 20.9 | (12.7, 29.1) | 6.8 | (4.7, 8.9) | 263.7 | (243.0, 284.4) | 86.4 | (76.9, 95.9) |
| Beer | 102.9 | (94.9, 110.9) | 18.5 | (11.6, 25.5) | 3.6 | (2.3, 4.9) | 216.7 | (197.5, 235.9) | 43.0 | (35.1, 50.8) |
| Wine | 24.1 | (21.7, 26.5) | 0.4 | (0.0, 0.7)* | 0.3 | (0.1, 0.5) | 32.2 | (27.5, 36.9) | 30.1 | (26.0 34.1) |
| Cocktails | 7.6 | (5.6, 9.5) | 1.1 | (0.3, 1.8) | 1.8 | (0.8, 2.8) | 9.3 | (5.2, 13.4) | 9.5 | (6.6, 12.3) |
| Spirits | 3.1 | (2.3, 3.9) | 0.7 | (0.0, 1.6)* | 0.4 | (0.1, 0.7) | 5.1 | (3.9, 6.2) | 2.6 | (0.9, 4.2) |
| Coolers | 0.5 | (0.1, 0.9) | 0.001 | (0.000, 0.003)* | 0.6 | (0.0, 1.6)* | 0.1 | (0.0, 0.3)* | 0.9 | (0.0, 1.9)* |
| Liqueur | 0.3 | (0.2, 0.4) | 0.2 | (0.0, 0.7)* | 0.1 | (0.0, 0.3)* | 0.27 | (0.05, 0.50) | 0.4 | (0.2, 0.6) |
| **100% juice** | 124.9 | (119.6, 130.2) | 185.3 | (174.6, 196.1) | 159.0 | (149.4, 168.7) | 126.1 | (115.1, 137.1) | 95.5 | (88.4, 102.6) |
| **Diet or light beverages** | 52.2 | (48.0, 56.4) | 20.0 | (16.1, 23.8) | 22.7 | (19.0, 26.4) | 58.0 | (49.6, 66.5) | 64.6 | (57.2, 71.9) |
| Diet or light carbonated soft drinks | 43.3 | (39.4, 47.2) | 13.3 | (10.3, 16.4) | 17.6 | (14.2, 20.9) | 48.7 | (40.4, 57.0) | 54.3 | (48.0, 60.7) |
| Diet or light fruit drinks | 5.0 | (3.5, 6.4) | 4.2 | (2.8, 5.6) | 4.2 | (2.6, 5.7) | 4.4 | (2.1, 6.8) | 6.0 | (3.0, 9.0) |
| Diet or light meal replacement beverages | 3.3 | (2.5, 4.2) | 1.9 | (0.4, 3.5) | 0.4 | (0.0, 0.9)* | 4.3 | (2.7, 5.9) | 3.6 | (2.2, 5.1) |
| Diet or light hot chocolate | 0.3 | (0.1, 0.5) | 0.2 | (0.0, 0.4)* | 0.6 | (0.1, 1.1) | 0.160 | (0.001, 0.318) | 0.4 | (0.1, 0.8) |
| Diet or light coffee | 0.2 | (0.0, 0.4)* | - | - | 0.05 | (0.00, 0.16)* | 0.3 | (0.0, 0.9)* | 0.1 | (0.0, 0.4)* |
| Diet or light sports drinks | 0.1 | (0.0, 0.2)* | 0.2 | (0.0, 0.6)* | 0.01 | (0.00, 0.02)* | 0.1 | (0.0, 0.3)* | 0.01 | (0.00, 0.04)* |

All means are weighted ‘arithmetic means’ (also referred to as ‘observed means’). Hyphen ‘-‘ denotes no volume consumed. In the 2004 data, there was no reported consumption of: unsweetened flavoured milk (type of ‘Club soda and other’ in the ‘Other unsweetened’ category), regular flavoured water (SSBs category), regular protein drinks (SSBs category), tea sweetened at the table by the consumer (type of ‘Other SSBs’ in the SSBs category), diet or light energy drinks (diet or light beverages category), diet or light flavoured water (diet or light beverages category), diet or light tea (diet or light beverages category), or diet or light protein drinks (diet or light beverages category).

^a^The 2004 ‘Club soda and other’ sub-category consists of club soda and unsweetened hot chocolate.

^b^The 2004 ‘Other SSBs’ sub-category consists of coffee sweetened by the consumer (i.e. ‘at the table’) and homemade hot chocolate prepared from scratch.

Abbrevations: 95% CI, 95% confidence interval; ml, milliliter; SSBs, sugar-sweetened beverages

*Estimates for these 95% confidence intervals contained values less than zero. For reporting, these values were replaced with ‘0’ as they were a result of the bootstrap resampling method and not an indication of negative consumption.

## Supplementary File Table 12. Daily per capita energy intake (kcal) from beverage categories and sub-categories in 2004, by all and age-sex group

|  | **ALL** | | **CHILDREN AND YOUTH 1-18 yrs** | | | | **ADULTS 19+ yrs** | | | | |
| --- | --- | --- | --- | --- | --- | --- | --- | --- | --- | --- | --- |
|  |  | | **Males** | | **Females** | | **Males** | | **Females** | |  |
|  | **Mean energy in ml**  **(95% CI)** | | **Mean energy in ml**  **(95% CI)** | | **Mean energy in ml**  **(95% CI)** | | **Mean energy in ml**  **(95% CI)** | | **Mean energy in ml**  **(95% CI)** | |  |
| n | N=34363 | | n=4009 | | n=3843 | | n=13321 | | n=13291 | |  |
| **Plain water** | - | - | - | - | - | - | - | - | - | - |  |
| Other water (tap, well) | - | - | - | - | - | - | - | - | - | - |  |
| Bottled water | - | - | - | - | - | - | - | - | - | - |  |
| **Other unsweetened** | 9.6 | (9.1, 10.2) | 0.8 | (0.6, 1.0) | 1.3 | (0.9, 1.6) | 12.5 | (11.4, 13.6) | 11.8 | (10.9, 12.7) |  |
| Unsweetened coffee | 8.4 | (7.8, 8.9) | 0.5 | (0.3, 0.8) | 1.0 | (0.6, 1.3) | 11.2 | (10.0, 12.3) | 10.0 | (9.1, 10.9) |  |
| Unsweetened tea | 1.2 | (1.2, 1.3) | 0.3 | (0.2, 0.3) | 0.3 | (0.2, 0.3) | 1.3 | (1.2, 1.4) | 1.8 | (1.7, 1.9) |  |
| Club soda and other^a^ | 0.009 | (0.001, 0.017) | - | - | 0.01 | (0.00, 0.02)* | - | - | 0.02 | (0.00, 0.04)* |  |
| **SSBs** | 123.5 | (119.7, 127.2) | 214.1 | (204.5, 223.7) | 158.4 | (151.0, 165.8) | 129.1 | (121.5, 136.8) | 80.3 | (75.1, 85.5) |  |
| Regular carbonated soft drinks | 53.9 | (51.3, 56.5) | 73.5 | (68.0, 79.1) | 40.5 | (37.1, 43.8) | 72.0 | (66.6, 77.5) | 33.6 | (30.3, 36.9) |  |
| Regular fruit drinks | 37.5 | (35.6, 39.4) | 78.0 | (72.6, 83.3) | 70.2 | (65.5, 74.9) | 29.0 | (25.6, 32.4) | 24.4 | (21.9, 26.9) |  |
| Sugar-sweetened milk | 17.2 | (15.7, 18.7) | 44.8 | (39.5, 50.0) | 33.2 | (29.1, 37.3) | 12.2 | (9.5, 14.9) | 9.2 | (7.2, 11.1) |  |
| Tea pre-sweetened with sugar | 4.6 | (3.9, 5.2) | 5.7 | (4.1, 7.2) | 3.9 | (2.9, 4.8) | 4.3 | (3.3, 5.4) | 4.7 | (3.4, 6.0) |  |
| Regular sports drinks | 2.0 | (1.5, 2.6) | 3.6 | (2.5, 4.7) | 1.4 | (1.0, 1.9) | 3.0 | (1.8, 4.3) | 0.8 | (0.2, 1.3) |  |
| Hot chocolate pre-sweetened with sugar | 3.5 | (2.8, 4.2) | 4.5 | (3.1, 5.9) | 4.9 | (3.5, 6.2) | 3.8 | (2.2, 5.4) | 2.5 | (1.8, 3.2) |  |
| Smoothies | 2.5 | (1.2, 3.8) | 2.1 | (1.1, 3.2) | 2.6 | (1.6, 3.6) | 2.9 | (0.0, 6.0)* | 2.3 | (1.3, 3.2) |  |
| Coffee pre-sweetened with sugar | 1.0 | (0.7, 1.2) | 0.5 | (0.2, 0.8) | 0.5 | (0.2, 0.8) | 0.7 | (0.4, 1.0) | 1.5 | (1.0, 2.0) |  |
| Regular meal replacement beverages | 0.9 | (0.7, 1.2) | 1.1 | (0.5, 1.7) | 0.7 | (0.2, 1.3) | 0.7 | (0.3, 1.1) | 1.2 | (0.7, 1.7) |  |
| Other SSBs^b^ | 0.3 | (0.1, 0.5) | 0.17 | (0.02, 0.33) | 0.5 | (0.1, 1.0) | 0.3 | (0.0, 0.7)* | 0.3 | (0.1, 0.5) |  |
| Regular energy drinks | 0.05 | (0.00, 0.11)* | - | - | 0.01 | (0.00, 0.03)* | 0.1 | (0.0, 0.2)* | 0.04 | (0.00, 0.11)* |  |
| Flavoured drinkable yogurt | 0.04 | (0.01, 0.08) | 0.1 | (0.0, 0.3)* | - | - | 0.1 | (0.0, 0.1)* | 0.02 | (0.00, 0.05)* |  |
| **Plain milk** | 99.4 | (96.7, 102.1) | 183.0 | (175.9, 190.1) | 144.2 | (137.5, 150.9) | 84.4 | (79.2, 89.6) | 76.2 | (72.2, 80.2) |  |
| **Alcoholic beverages** | 72.8 | (68.4, 77.2) | 11.5 | (5.1, 18.0) | 4.7 | (3.2, 6.2) | 129.3 | (119.6, 138.9) | 54.4 | (47.8, 60.9) |  |
| Beer | 40.7 | (37.5, 44.0) | 7.6 | (4.8, 10.5) | 1.4 | (0.9, 1.9) | 86.0 | (78.3, 93.8) | 16.7 | (13.5, 19.9) |  |
| Wine | 17.3 | (15.6, 19.0) | 0.3 | (0.0, 0.5)* | 0.20 | (0.03, 0.36) | 23.3 | (19.9, 26.7) | 21.4 | (18.6, 24.3) |  |
| Cocktails | 6.4 | (4.9, 7.9) | 1.2 | (0.2, 2.2) | 1.5 | (0.7, 2.3) | 7.3 | (4.8, 9.8) | 8.6 | (5.7, 11.5) |  |
| Spirits | 7.1 | (5.2, 8.9) | 1.6 | (0.0, 3.7)* | 0.9 | (0.3, 1.5) | 11.7 | (8.9, 14.4) | 5.9 | (2.1, 9.7) |  |
| Coolers | 0.24 | (0.05, 0.44) | 0.001 | (0.000, 0.002)* | 0.3 | (0.0, 0.8)* | 0.1 | (0.0, 0.2)* | 0.5 | (0.0, 0.9)* |  |
| Liqueur | 1.0 | (0.5, 1.5) | 0.8 | (0.0, 2.2)* | 0.4 | (0.0, 0.8)* | 0.9 | (0.2, 1.7) | 1.3 | (0.6, 2.0) |  |
| **100% juice** | 54.2 | (51.8, 56.5) | 83.1 | (78.2, 87.9) | 72.0 | (67.6, 76.5) | 53.8 | (48.9, 58.6) | 40.7 | (37.6, 43.7) |  |
| **Diet or light beverages** | 2.9 | (2.4, 3.4) | 1.8 | (0.6, 3.1) | 1.0 | (0.5, 1.5) | 3.5 | (2.5, 4.5) | 3.2 | (2.4, 3.9) |  |
| Diet or light carbonated soft drinks | 0.4 | (0.3, 0.4) | 0.1 | (0.1, 0.1) | 0.2 | (0.1, 0.2) | 0.4 | (0.3, 0.5) | 0.5 | (0.4, 0.5) |  |
| Diet or light fruit drinks | 0.3 | (0.2, 0.4) | 0.2 | (0.1, 0.4) | 0.3 | (0.1, 0.4) | 0.2415 | (0.0003, 0.4826) | 0.3 | (0.1, 0.5) |  |
| Diet or light meal replacement beverages | 2.1 | (1.6, 2.6) | 1.4 | (0.2, 2.7) | 0.4 | (0.0, 0.8)* | 2.7 | (1.7, 3.7) | 2.2 | (1.5, 2.9) |  |
| Diet or light hot chocolate | 0.11 | (0.05, 0.17) | 0.070 | (0.002, 0.137) | 0.20 | (0.05, 0.35) | 0.1 | (0.0, 0.2)* | 0.14 | (0.02, 0.27) |  |
| Diet or light coffee | 0.03 | (0.00, 0.07)* | - | - | 0.01 | (0.00, 0.02)* | 0.1 | (0.0, 0.1)* | 0.02 | (0.00, 0.06)* |  |
| Diet or light sports drinks | 0.009 | (0.000, 0.019)* | 0.02 | (0.00, 0.06)* | 0.001 | (0.000, 0.002)* | 0.01 | (0.00, 0.04)* | 0.001 | (0.000, 0.005)* |  |

All means are weighted ‘arithmetic means’ (also referred to as ‘observed means’). Hyphen ‘-‘ denotes no energy consumed. In the 2004 data, there was no reported consumption of: unsweetened flavoured milk (type of ‘Club soda and other’ in the ‘Other unsweetened’ category), regular flavoured water (SSBs category), regular protein drinks (SSBs category), tea sweetened at the table by the consumer (type of ‘Other SSBs’ in the SSBs category), diet or light energy drinks (diet or light beverages category), diet or light flavoured water (diet or light beverages category), diet or light tea (diet or light beverages category), or diet or light protein drinks (diet or light beverages category).

^a^The 2004 ‘Club soda and other’ sub-category consists of club soda and unsweetened hot chocolate.

^b^The 2004 ‘Other SSBs’ sub-category consists of coffee sweetened by the consumer (i.e. ‘at the table’) and homemade hot chocolate prepared from scratch.

Abbreviations: 95% CI, 95% confidence interval; kcal, kilocalorie; SSBs, sugar-sweetened beverages

*Estimates for these 95% confidence intervals contained values less than zero. For reporting, these values were replaced with ‘0’ as they were a result of the bootstrap resampling method and not an indication of negative consumption.

# Appendix E: 2015 Additional analyses of national and age-sex consumption

## Supplementary File Table 13. Daily per capita volume intake (ml) from beverage categories and sub-categories in 2015, by all and age-sex group

|  | **ALL** | | **CHILDREN AND YOUTH 1-18 yrs** | | | | **ADULTS 19+ yrs** | | | |
| --- | --- | --- | --- | --- | --- | --- | --- | --- | --- | --- |
|  |  | | **Males** | | **Females** | | **Males** | | **Females** | |
|  | **Mean volume in ml**  **(95% CI)** | | **Mean volume in ml**  **(95% CI)** | | **Mean volume in ml**  **(95% CI)** | | **Mean volume in ml**  **(95% CI)** | | **Mean volume in ml**  **(95% CI)** | |
| n | N=20176 | | n=2208 | | n=2002 | | n=8069 | | n=8077 | |
| **Plain water** | 867.2 | (840.8, 893.7) | 680.6 | (647.1, 714.2) | 609.3 | (578.4, 640.3) | 928.8 | (877.3, 980.3) | 916.5 | (881.0, 952.0) |
| Other water (tap, well) | 613.4 | (588.1, 638.8) | 491.7 | (458.7, 524.8) | 460.1 | (431.0, 489.2) | 639.6 | (589.9, 689.2) | 655.9 | (622.3, 689.5) |
| Bottled water | 253.8 | (235.0, 272.6) | 188.9 | (165.8, 212.1) | 149.2 | (130.1, 168.4) | 289.3 | (254.3, 324.3) | 260.6 | (232.8, 288.4) |
| **Other unsweetened** | 364.4 | (352.9, 376.0) | 28.9 | (22.5, 35.3) | 27.7 | (22.8, 32.6) | 467.6 | (444.6, 490.6) | 429.1 | (411.1, 447.0) |
| Unsweetened coffee | 253.9 | (243.9, 263.8) | 12.0 | (8.4, 15.7) | 8.8 | (6.3, 11.4) | 357.4 | (336.8, 378.0) | 271.9 | (257.0, 286.7) |
| Unsweetened tea | 109.0 | (101.8, 116.1) | 16.7 | (11.4, 21.9) | 18.8 | (15.1, 22.4) | 108.6 | (95.9, 121.3) | 154.9 | (142.8, 167.0) |
| Club soda and other^a^ | 1.6 | (0.8, 2.4) | 0.2 | (0.0, 0.6)* | 0.1 | (0.0, 0.3)* | 1.64 | (0.04, 3.24) | 2.3 | (1.1, 3.5) |
| **SSBs** | 203.6 | (193.1, 214.0) | 260.1 | (237.8, 282.4) | 190.3 | (176.4, 204.1) | 243.5 | (223.0, 264.1) | 152.8 | (140.5, 165.2) |
| Regular carbonated soft drinks | 70.3 | (64.7, 76.0) | 77.1 | (65.6, 88.6) | 41.3 | (35.6, 46.9) | 99.8 | (88.2, 111.4) | 46.4 | (39.4, 53.5) |
| Regular fruit drinks | 29.5 | (25.9, 33.0) | 56.6 | (44.2, 69.0) | 49.4 | (42.1, 56.7) | 25.8 | (18.6, 33.0) | 21.4 | (17.8, 24.9) |
| Sugar-sweetened milk | 25.8 | (22.5, 29.0) | 46.2 | (38.0, 54.5) | 38.7 | (32.9, 44.6) | 26.8 | (20.3, 33.4) | 16.3 | (13.2, 19.5) |
| Tea pre-sweetened with sugar | 20.3 | (15.9, 24.8) | 20.7 | (15.2, 26.2) | 16.7 | (13.2, 20.2) | 25.4 | (15.5, 35.2) | 16.1 | (11.9, 20.3) |
| Regular sports drinks | 7.0 | (4.5, 9.4) | 14.2 | (9.2, 19.2) | 4.8 | (2.4, 7.2) | 10.4 | (4.6, 16.2) | 2.3 | (0.3, 4.2) |
| Hot chocolate pre-sweetened with sugar | 4.2 | (3.1, 5.3) | 7.8 | (5.2, 10.4) | 8.2 | (5.4, 11.0) | 2.0 | (1.2, 2.9) | 4.4 | (1.9, 6.9) |
| Smoothies | 16.6 | (13.2, 20.0) | 12.7 | (9.5, 15.8) | 10.8 | (8.0, 13.6) | 17.7 | (11.1, 24.4) | 17.8 | (13.3, 22.4) |
| Coffee pre-sweetened with sugar | 15.9 | (13.1, 18.7) | 5.8 | (3.0, 8.6) | 7.8 | (5.0, 10.5) | 19.0 | (14.2, 23.9) | 17.4 | (12.7, 22.0) |
| Regular meal replacement beverages | 1.7 | (1.2, 2.1) | 1.9 | (0.6, 3.1) | 2.2 | (0.7, 3.7) | 1.7 | (1.0, 2.5) | 1.5 | (0.9, 2.1) |
| Other SSBs^b^ | 0.7 | (0.3, 1.1) | 1.1 | (0.0, 2.9)* | 0.005 | (0.000, 0.016)* | 0.5 | (0.2, 0.9) | 0.9 | (0.2, 1.7) |
| Regular energy drinks | 1.7 | (1.0, 2.5) | 0.8 | (0.1, 1.5) | 0.3 | (0.0, 0.6)* | 3.0 | (1.3, 4.7) | 1.1 | (0.2, 1.9) |
| Flavoured drinkable yogurt | 2.4 | (1.9, 2.9) | 7.4 | (5.5, 9.2) | 8.3 | (5.7, 10.9) | 0.9 | (0.3, 1.6) | 1.2 | (0.6, 1.7) |
| Regular flavoured water | 1.7 | (0.9, 2.5) | 3.3 | (0.8, 5.7) | 1.3 | (0.2, 2.4) | 1.8 | (0.2, 3.4) | 1.3 | (0.4, 2.2) |
| Regular protein drinks | 5.8 | (4.3, 7.4) | 4.6 | (1.6, 7.6) | 0.5 | (0.0, 1.1)* | 8.5 | (4.9, 12.1) | 4.7 | (3.3, 6.2) |
| **Plain milk** | 131.8 | (126.0, 137.5) | 265.7 | (251.1, 280.3) | 217.6 | (203.5, 231.7) | 112.9 | (102.8, 123.0) | 95.7 | (87.7, 103.7) |
| **Alcoholic beverages** | 120.3 | (109.1, 131.4) | 6.9 | (3.4, 10.3) | 2.5 | (0.6, 4.5) | 220.7 | (194.9, 246.5) | 77.6 | (68.5, 86.7) |
| Beer | 82.7 | (72.6, 92.8) | 4.8 | (2.0, 7.6) | 0.4 | (0.0, 0.8)* | 175.5 | (151.4, 199.7) | 30.0 | (23.8, 36.1) |
| Wine | 27.6 | (24.5, 30.7) | 0.4 | (0.0, 1.1)* | 0.2 | (0.0, 0.5)* | 33.0 | (27.9, 38.2) | 35.8 | (30.3, 41.3) |
| Cocktails | 5.2 | (3.7, 6.8) | 1.2 | (0.0, 2.6)* | 0.9 | (0.0, 2.0)* | 5.3 | (3.0, 7.6) | 7.3 | (4.2, 10.3) |
| Spirits | 3.2 | (2.3, 4.2) | 0.4 | (0.0, 0.9)* | 0.08 | (0.02, 0.15) | 5.2 | (3.4, 7.1) | 2.7 | (1.3, 4.2) |
| Coolers | 1.1 | (0.5, 1.7) | 0.1 | (0.0, 0.2)* | 0.9 | (0.0, 2.4)* | 1.1 | (0.2, 2.1) | 1.4 | (0.3, 2.5) |
| Liqueur | 0.4 | (0.1, 0.6) | - | - | 0.01 | (0.00, 0.02)* | 0.51 | (0.01, 1.00) | 0.42 | (0.01, 0.83) |
| **100% juice** | 74.3 | (69.9, 78.7) | 131.4 | (120.7, 142.0) | 104.8 | (94.8, 114.7) | 74.4 | (65.9, 82.9) | 52.3 | (47.0, 57.6) |
| **Diet or light beverages** | 44.1 | (38.3, 50.0) | 12.9 | (8.6, 17.1) | 11.3 | (8.4, 14.3) | 56.5 | (45.6, 67.4) | 47.8 | (39.0, 56.6) |
| Diet or light carbonated soft drinks | 31.7 | (26.9, 36.6) | 5.3 | (3.4, 7.2) | 4.2 | (2.4, 5.9) | 42.4 | (32.8, 51.9) | 34.6 | (26.9, 42.2) |
| Diet or light fruit drinks | 2.8 | (1.7, 3.9) | 1.8 | (0.9, 2.7) | 3.0 | (1.4, 4.6) | 3.5 | (1.2, 5.7) | 2.3 | (0.7, 3.9) |
| Diet or light meal replacement beverages | 0.5 | (0.0, 1.1)* | 0.01 | (0.00, 0.05)* | - | - | 0.15 | (0.04, 0.26) | 1.2 | (0.0, 2.6)* |
| Diet or light hot chocolate | 0.035 | (0.005, 0.066) | 0.1 | (0.0, 0.2)* | 0.1 | (0.0, 0.4)* | 0.01 | (0.00, 0.02)* | 0.03 | (0.00, 0.07)* |
| Diet or light coffee | 0.01 | (0.00, 0.02)* | - | - | - | - | 0.01 | (0.00, 0.02)* | 0.01 | (0.00, 0.03)* |
| Diet or light sports drinks | 1.2 | (0.3, 2.1) | 0.44 | (0.03, 0.84) | 0.1 | (0.0, 0.3)* | 2.4 | (0.2, 4.5) | 0.5 | (0.0, 1.0)* |
| Diet or light energy drinks | 0.7 | (0.3, 1.2) | 1.8 | (0.0, 4.6)* | 0.02 | (0.00, 0.06)* | 0.8 | (0.1, 1.6) | 0.5 | (0.1, 1.0) |
| Diet or light flavoured water | 5.2 | (3.3, 7.2) | 2.9 | (0.9, 4.9) | 3.0 | (1.4, 4.7) | 5.3 | (1.7, 8.9) | 6.3 | (3.0, 9.7) |
| Diet or light tea | 1.8 | (1.2, 2.5) | 0.6 | (0.0, 1.4)* | 0.5 | (0.1, 0.9) | 1.9 | (0.7, 3.2) | 2.4 | (1.4, 3.3) |
| Diet or light protein drinks | 0.1 | (0.0, 0.1)* | - | - | 0.4 | (0.0, 1.2)* | 0.03 | (0.00, 0.08)* | 0.001 | (0.000, 0.004)* |

All means are weighted ‘arithmetic means’ (also referred to as ‘observed means’). Hyphen ‘-‘ denotes no volume consumed. In the 2015 data, there was no reported consumption of: unsweetened hot chocolate (type of ‘Club soda and other’ in the ‘Other unsweetened’ category).

^b^The 2015 ‘Club soda and other’ sub-category consists of club soda and unsweetened flavoured milk.

^c^The 2015 ‘Other SSBs’ sub-category consists of coffee sweetened by the consumer (i.e. ‘at the table’), tea sweetened by the consumer, and homemade hot chocolate prepared from scratch.

Abbreviations: 95% CI, 95% confidence interval; ml, milliliter; SSBs, sugar-sweetened beverages

*Estimates for these 95% confidence intervals contained values less than zero. For reporting, these values were replaced with ‘0’ as they were a result of the bootstrap resampling method and not an indication of negative consumption.

## Supplementary File Table 14. Daily per capita energy intake (kcal) from beverage categories and sub-categories in 2015, by all and age-sex group

|  | **ALL** | | **CHILDREN AND YOUTH 1-18 yrs** | | | | **ADULTS 19+ yrs** | | | | |
| --- | --- | --- | --- | --- | --- | --- | --- | --- | --- | --- | --- |
|  |  | | **Males** | | **Females** | | **Males** | | **Females** | |  |
|  | **Mean energy in ml**  **(95% CI)** | | **Mean energy in ml**  **(95% CI)** | | **Mean energy in ml**  **(95% CI)** | | **Mean energy in ml**  **(95% CI)** | | **Mean energy in ml**  **(95% CI)** | |  |
| n | N=20176 | | n=2208 | | n=2002 | | n=8069 | | n=8077 | |  |
| **Plain water** | - | - | - | - | - | - | - | - | - | - |  |
| Other water (tap, well) | - | - | - | - | - | - | - | - | - | - |  |
| Bottled water | - | - | - | - | - | - | - | - | - | - |  |
| **Other unsweetened** | 5.5 | (4.5, 6.6) | 0.7 | (0.0, 1.5)* | 0.6 | (0.2, 1.0) | 6.2 | (3.8, 8.6) | 7.3 | (6.2, 8.5) |  |
| Unsweetened coffee | 4.4 | (3.3, 5.5) | 0.5 | (0.0, 1.2)* | 0.41 | (0.02, 0.80) | 5.1 | (2.8, 7.5) | 5.6 | (4.5, 6.8) |  |
| Unsweetened tea | 1.1 | (1.0, 1.2) | 0.2 | (0.1, 0.4) | 0.2 | (0.2, 0.2) | 1.1 | (1.0, 1.2) | 1.7 | (1.5, 1.9) |  |
| Club soda and other^a^ | 0.004 | (0.000, 0.010)* | - | - | 0.02 | (0.00, 0.07)* | 0.004 | (0.000, 0.0120)* | 0.0002 | (0.0000, 0.0004)* |  |
| **SSBs** | 98.7 | (93.5, 103.9) | 133.6 | (121.8, 145.4) | 100.5 | (92.7, 108.3) | 116.4 | (105.9, 127.0) | 71.8 | (65.5, 78.1) |  |
| Regular carbonated soft drinks | 28.6 | (26.3, 30.9) | 31.4 | (26.7, 36.0) | 17.0 | (14.7, 19.4) | 40.8 | (36.1, 45.5) | 18.6 | (15.8, 21.5) |  |
| Regular fruit drinks | 13.7 | (12.0, 15.3) | 25.8 | (19.9, 31.7) | 21.9 | (18.6, 25.1) | 12.2 | (8.8, 15.6) | 10.1 | (8.4, 11.8) |  |
| Sugar-sweetened milk | 18.5 | (15.3, 21.8) | 33.7 | (27.7, 39.7) | 27.6 | (23.4, 31.7) | 20.7 | (13.5, 27.9) | 10.3 | (7.7, 12.9) |  |
| Tea pre-sweetened with sugar | 6.9 | (5.3, 8.5) | 7.2 | (5.3, 9.1) | 5.8 | (4.6, 7.1) | 8.7 | (5.2, 12.2) | 5.3 | (3.9, 6.7) |  |
| Regular sports drinks | 1.9 | (1.2, 2.5) | 3.9 | (2.5, 5.3) | 1.3 | (0.6, 1.9) | 2.8 | (1.3, 4.3) | 0.6 | (0.1, 1.1) |  |
| Hot chocolate pre-sweetened with sugar | 3.4 | (2.4, 4.5) | 6.6 | (4.3, 9.0) | 6.0 | (3.7, 8.2) | 1.6 | (0.8, 2.3) | 3.8 | (1.4, 6.2) |  |
| Smoothies | 10.2 | (8.0, 12.3) | 7.6 | (5.8, 9.5) | 7.0 | (5.2, 8.9) | 11.0 | (6.7, 15.2) | 10.7 | (8.0, 13.5) |  |
| Coffee pre-sweetened with sugar | 6.1 | (4.9, 7.4) | 3.9 | (1.9, 5.8) | 4.8 | (3.1, 6.6) | 7.4 | (4.8, 10.0) | 5.8 | (4.1, 7.4) |  |
| Regular meal replacement beverages | 1.7 | (1.2, 2.1) | 1.8 | (0.6, 3.0) | 2.1 | (0.7, 3.6) | 1.7 | (1.0, 2.4) | 1.5 | (0.8, 2.1) |  |
| Other SSBs^b^ | 0.4 | (0.1, 0.6) | 0.8 | (0.0, 2.1)* | 0.003 | (0.000, 0.009)* | 0.3 | (0.1, 0.5) | 0.4 | (0.1, 0.8) |  |
| Regular energy drinks | 0.8 | (0.4, 1.1) | 0.37 | (0.05, 0.69) | 0.1 | (0.0, 0.2)* | 1.4 | (0.6, 2.2) | 0.4 | (0.1, 0.8) |  |
| Flavoured drinkable yogurt | 1.8 | (1.4, 2.1) | 5.5 | (4.1, 6.8) | 6.1 | (4.2, 8.1) | 0.7 | (0.2, 1.2) | 0.8 | (0.4, 1.2) |  |
| Regular flavoured water | 0.4 | (0.2, 0.6) | 0.8 | (0.2, 1.4) | 0.30 | (0.04, 0.55) | 0.4 | (0.1, 0.8) | 0.3 | (0.1, 0.5) |  |
| Regular protein drinks | 4.4 | (2.8, 6.1) | 4.2 | (1.5, 6.9) | 0.5 | (0.0, 1.1)* | 6.8 | (2.9, 10.8) | 3.1 | (2.2, 4.0) |  |
| **Plain milk** | 64.1 | (61.1, 67.1) | 134.3 | (126.7, 141.8) | 108.8 | (101.6, 116.0) | 54.2 | (48.9, 59.5) | 45.3 | (41.2, 49.4) |  |
| **Alcoholic beverages** | 71.0 | (64.8, 77.2) | 4.2 | (1.8, 6.7) | 1.8 | (0.5, 3.1) | 119.6 | (106.0, 133.2) | 56.3 | (49.2, 63.3) |  |
| Beer | 33.8 | (29.5, 38.1) | 2.0 | (0.8, 3.2) | 0.2 | (0.0, 0.4)* | 72.2 | (62.0, 82.5) | 11.8 | (9.3, 14.2) |  |
| Wine | 23.2 | (20.6, 25.8) | 0.3 | (0.0, 0.9)* | 0.2 | (0.0, 0.5)* | 28.1 | (23.7, 32.5) | 29.8 | (25.2, 34.3) |  |
| Cocktails | 4.6 | (3.1, 6.1) | 1.0 | (0.0, 2.1)* | 0.7 | (0.0, 1.7)* | 4.9 | (2.5, 7.3) | 6.2 | (3.6, 8.8) |  |
| Spirits | 7.5 | (5.3, 9.7) | 0.8 | (0.0, 2.1)* | 0.19 | (0.04, 0.34) | 12.1 | (7.8, 16.4) | 6.3 | (3.0, 9.6) |  |
| Coolers | 0.6 | (0.3, 1.0) | 0.04 | (0.00, 0.13)* | 0.5 | (0.0, 1.4)* | 0.6 | (0.1, 1.2) | 0.8 | (0.2, 1.4) |  |
| Liqueur | 1.2 | (0.4, 2.1) | - | - | 0.02 | (0.00, 0.07)* | 1.70 | (0.05, 3.36) | 1.40 | (0.03, 2.78) |  |
| **100% juice** | 33.7 | (31.7, 35.7) | 61.4 | (56.3, 66.4) | 49.2 | (44.5, 53.9) | 32.9 | (29.0, 36.9) | 23.7 | (21.2, 26.2) |  |
| **Diet or light beverages** | 1.6 | (1.1, 2.1) | 0.5 | (0.3, 0.7) | 0.7 | (0.3, 1.0) | 1.6 | (1.3, 2.0) | 2.0 | (0.9, 3.1) |  |
| Diet or light carbonated soft drinks | 0.5 | (0.4, 0.6) | 0.08 | (0.05, 0.11) | 0.06 | (0.03, 0.09) | 0.7 | (0.5, 0.8) | 0.5 | (0.4, 0.6) |  |
| Diet or light fruit drinks | 0.3 | (0.2, 0.4) | 0.2 | (0.1, 0.3) | 0.3 | (0.1, 0.5) | 0.4 | (0.2, 0.6) | 0.3 | (0.1, 0.4) |  |
| Diet or light meal replacement beverages | 0.4 | (0.0, 0.9)* | 0.01 | (0.00, 0.04)* | - | - | 0.12 | (0.03, 0.20) | 0.9 | (0.0, 2.0)* |  |
| Diet or light hot chocolate | 0.012 | (0.002, 0.021) | 0.02 | (0.00, 0.05)* | 0.04 | (0.00, 0.11)* | 0.003 | (0.000, 0.010)* | 0.01 | (0.00 0.02)* |  |
| Diet or light coffee | 0.002 | (0.000, 0.005)* | - | - | - | - | 0.002 | (0.000, 0.006)* | 0.003 | (0.000, 0.010)* |  |
| Diet or light sports drinks | 0.13 | (0.04, 0.23) | 0.048 | (0.003, 0.093) | 0.01 | (0.00, 0.03)* | 0.26 | (0.03, 0.50) | 0.1 | (0.0, 0.1)* |  |
| Diet or light energy drinks | 0.04 | (0.02, 0.07) | 0.1 | (0.0, 0.3)* | 0.001 | (0.000, 0.004)* | 0.05 | (0.01, 0.10) | 0.03 | (0.01, 0.06) |  |
| Diet or light flavoured water | 0.10 | (0.03, 0.17) | 0.04 | (0.01, 0.08) | 0.04 | (0.01, 0.07) | 0.06 | (0.02, 0.10) | 0.2 | (0.0, 0.3)* |  |
| Diet or light tea | 0.04 | (0.03, 0.06) | 0.01 | (0.00, 0.03)* | 0.011 | (0.002, 0.019) | 0.05 | (0.02, 0.08) | 0.06 | (0.03, 0.08) |  |
| Diet or light protein drinks | 0.02 | (0.00, 0.05)* | - | - | 0.2 | (0.0, 0.4)* | 0.01 | (0.00, 0.03)* | 0.0004 | (0.0000, 0.0013)* |  |

All means are weighted ‘arithmetic means’ (also referred to as ‘observed means’). Hyphen ‘-‘ denotes no energy consumed. In the 2015 data, there was no reported consumption of: unsweetened hot chocolate (type of ‘Club soda and other’ in the ‘Other unsweetened’ category).

^a^The 2015 ‘Club soda and other’ sub-category consists of club soda and unsweetened flavoured milk.

^b^The 2015 ‘Other SSBs’ sub-category consists of coffee sweetened by the consumer (i.e. ‘at the table’), tea sweetened by the consumer, and homemade hot chocolate prepared from scratch.

Abbreviations: 95% CI, 95% confidence interval; kcal, kilocalorie; SSBs, sugar-sweetened beverages

*Estimates for these 95% confidence intervals contained values less than zero. For reporting, these values were replaced with ‘0’ as they were a result of the bootstrap resampling method and not an indication of negative consumption.
